# KRAB-type zinc-finger proteins PITA and PISA specifically regulate p53-dependent glycolysis and mitochondrial respiration

**DOI:** 10.1038/s41422-018-0008-8

**Published:** 2018-02-21

**Authors:** Shan Wang, Zhiqiang Peng, Siying Wang, Lihua Yang, Yuhan Chen, Xue Kong, Shanshan Song, Pei Pei, Chunyan Tian, Hui Yan, Peipei Ding, Weiguo Hu, Cui Hua Liu, Xin Zhang, Fuchu He, Lingqiang Zhang

**Affiliations:** 1 State Key Laboratory of Proteomics, Beijing Proteome Research Center, National Center of Protein Sciences (Beijing), Beijing Institute of Lifeomics, Beijing, China; 2 Department of Genomics and Proteomics, Beijing Institute of Radiation Medicine, Beijing, China; 3 Beijing Municipal Key Laboratory of Child Development and Nutriomics, Capital Institute of Pediatrics, Beijing, China; 4 Department of Biochemistry and Molecular Biology, Anhui Medical University, Anhui, China; 5 State Key Laboratory of Toxicology and Medical Countermeasures, Beijing Institute of Pharmacology and Toxicology, Beijing, China; 6 Fudan University Shanghai Cancer Center and Institutes of Biomedical Sciences, Collaborative Innovation Center of Cancer Medicine, Shanghai Medical College, Fudan University, Shanghai, China; 7 CAS Key Laboratory of Pathogenic Microbiology and Immunology, Institute of Microbiology, Chinese Academy of Sciences, Beijing, China; 8 State Key Laboratory of Cell Stress Biology, School of Life Sciences, Xiamen University, Fujian, China; 9 School of Life Sciences, Jiangsu Normal University, Jiangsu, China

## Abstract

Few p53 regulators participate in selective control of p53-mediated cellular metabolism. How p53-mediated aerobic and glycolytic pathways are negatively regulated remains largely unclear. Here, we identified two KRAB-type zinc-finger proteins, PITA (p53 inhibitor of TIGAR activation) and PISA (p53 inhibitor of SCO2 activation), as selective regulators of p53 in metabolic control. PITA and PISA interact with p53 and specifically suppress transcription of the glycolysis regulator TIGAR and the oxidation phosphorylation regulator SCO2, respectively. Importantly, PITA transgenic mice exhibited increased 6-phosphofructokinase 1 (PFK1) activity and an elevated glycolytic rate, whereas PISA transgenic mice had decreased cytochrome c oxidase activity and reduced mitochondrial respiration. In response to glucose starvation, PITA dissociates from p53, resulting in activation of p53 and induction of TIGAR, which inhibited aerobic glycolysis. Prolonged starvation leads to PISA dissociation from p53 and induction of SCO2 and p53-promoted mitochondrial respiration. The dynamic regulation of PITA and PISA upon metabolic stress is dependent on ATM kinase-mediated phosphorylation of PITA and PISA. Furthermore, in human colorectal cancers, the elevated expression of PITA and PISA correlates with cancer progression. Depletion of PITA or PISA in colorectal cancer cells reduced the cell proliferation, migration and invasion. These results identify PITA and PISA as selective regulators of p53-mediated glycolysis and mitochondrial respiration and provide novel insights into the role of p53 network in cell metabolic control.

## Introduction

The tumor suppressor p53 plays an important role in controlling of cell cycle arrest, DNA repair and apoptosis. Nevertheless, it remains unclear whether it is involved in the rate-limiting steps of tumor suppression. Emerging data are revealing that regulation of energy metabolism and the Warburg effect is a novel function of p53 in tumor suppression.^1^ Interestingly, tumor suppression can be mediated by a p53 polypeptide (e.g. p53^3KR^) that lacks the ability to induce p53-dependent cell cycle arrest, apoptosis and senescence. These results indicate that other p53 functions are sufficient to suppress tumor formation. The p53^3KR^ mutant retains the capacity to inhibit glycolysis and reduce reactive oxygen species (ROS) levels. These results suggest that unconventional activities of p53, such as metabolic regulation and antioxidant function, are crucial to the suppression of early onset spontaneous tumorigenesis.^1, 2^ p53 has a role in modulating metabolism, including glycolysis and oxidative phosphorylation (OXPHOS),^3^ and can prevent metabolic transformation by restraining the glycolytic pathway. A previous report showed that p53 induces TIGAR (TP53-induced glycolysis and apoptosis regulator) to decrease PFK1 (6-phosphofructokinase 1) activity and reduce the glycolytic rate.^4^ The restriction of glycolytic flux by p53 is paralleled by the ability of p53 to drive OXPHOS and maintain mitochondrial integrity. p53 transcriptionally activates SCO2 (synthesis of cytochrome c oxidase 2) to promote mitochondrial respiration. In the absence of p53, SCO2 levels decrease, shifting ATP generation from the oxidative phosphorylation pathway to glycolysis, a phenomenon widely observed in cancer cells and known as the Warburg effect.^5^

Selective regulation of target genes is generally achieved by post-translational modifications of p53 or through its interaction with various regulators. The Kruppel-associated box (KRAB) is a domain of about 75 amino acids that is found in the N-terminal region of approximately half of eukaryotic Kruppel-type C2H2 zinc-finger proteins (ZFPs).^6^ KRAB-ZFPs, also known as KZNF proteins, probably constitute the single-largest class of transcription factors within the human genome. Although the function of KZNFs is largely unknown, they appear to play important roles in cell differentiation and development.^7, 8^ Moreover, the fact that all KZNFs are tetrapod-specific suggests that they are involved in key aspects of vertebrate development.^8^ Emerging evidence links transcriptional repression mediated by KZNF proteins to cell proliferation, metabolism, apoptosis and cancer.^9^ However, despite their numerical abundance, little is currently known about the gene targets and the physiological functions of KZNF proteins. We previously showed that the KZNF protein Apak (ATM and p53-associated KZNF protein, also known as ZNF420) specifically inhibits p53-mediated apoptosis and has no significant effect on the transcription of cell cycle arrest-related genes.^10, 11^ The zinc-finger repeats of Apak contribute to determining the selective specificity of target gene recognition.^12^ Considering that the critical domains of Apak (e.g., the KRAB domain and the zinc-finger repeats) are also observed among other members of the KZNF superfamily, we then hypothesized that the specific family members might selectively regulate certain subset of p53 target genes and the corresponding downstream outputs, including cell metabolism. Currently, how p53-mediated metabolism is negatively regulated remains largely unclear. The purpose of this study was to screen KZNF family proteins for selective regulators of p53 in cell metabolic control.

Here, we identified ZNF475 (here named PITA) and ZNF568 (here named PISA) as specific regulators of p53 in controlling glycolytic rate and mitochondrial respiration, respectively. PITA and PISA bind directly to p53, and consequently the complex is preferentially recruited to the *TIGAR* or *SCO2* gene, respectively. PITA transgenic mice increased PFK1 activity and elevated glycolytic rates. PISA transgenic mice showed decreased cytochrome c oxidase activity and lower mitochondrial respiration. In response to metabolic stress, PITA and PISA successively dissociated from p53, activating p53, dampening aerobic glycolysis and promoting mitochondrial respiration. These findings provide the first evidence that KZNF proteins selectively regulate p53-mediated metabolism.

## Results

### Identification of PITA and PISA as selective regulators of p53 activity

**Fig. 1:**
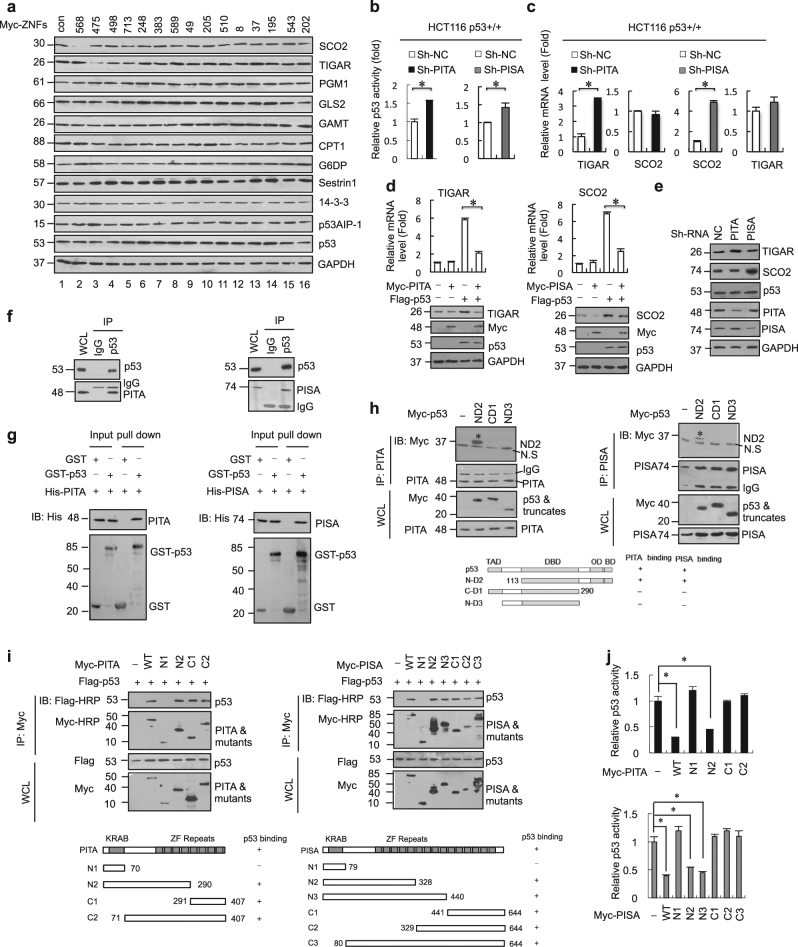
Identification of PITA and PISA as selective regulators of p53. **a** The KZNF members were each transfected into HCT116 cells and the expression level of metabolism-related p53 target genes were analyzed by immunoblotting with the indicated antibodies. **b** ShRNA-mediated knockdown of endogenous PITA or PISA augments p53 activity. p53 activity in HCT116 p53^+/+^ cells was measured by pG13L luciferase reporter gene assay Representative results of three independent reporter assay experiments are shown. **c** The mRNA levels of TIGAR or SCO2 in knock-down PITA or PISA HCT116 p53^+/+^ cells were measured by real-time PCR. **d** PITA or PISA selectively regulates expression of TIGAR or SCO2 dependent on p53. Total RNA from transfected HCT116 p53^+/+^ or HCT116 p53^−/−^ cells was subjected to real-time quantitative PCR (qPCR) analysis. **e** The expression levels of TIGAR or SCO2 in knockdown PITA or PISA HCT116 p53^+/+^ cells were measured byimmunoblotting with the indicated antibodies. **f** Coimmunoprecipitation of endogenous PITA or PISA and endogenous p53 from HCT116 cells. Western blot analysis of whole-cell lysate (WCL) and immunoprecipitation (IP) with preimmune serum, PITA or PISA-specific antibody, p53 antibody or control mouse IgG are shown. **g** Direct interaction between PITA and p53, PISA and p53 revealed by GST pull-down assays. Both input and pull-down samples were subjected to immunoblotting (IB) with anti-His antibody. Input represents 10% of that used for pull-down. The bottom part shows the gel stained by Coomassie brilliant blue. **h** Mapping the region of p53 that interacts with PITA or with PISA. Myc-tagged p53 mutants were transfected into HCT116 p53^−/−^ cells. Endogenous PITA or PISA proteins were immunoprecipitated with anti-PITA or PISA antibody. IgG HC, heavy chain of IgG; TAD, transactivation domain; DBD, DNA-binding domain; OD, oligomerization domain; BD, basic domain. **i** Interaction region mapping on PITA or PISA. Full-length or truncations of PITA or PISA and Flag-p53 were transfected into HCT116 p53^+/+^ cells. WCL was immunoprecipitated with anti-Flag antibody. Both the WCL and IP samples were analyzed. **j** Both KRAB domain and zinc-fingers are required for inhibition of p53 activity by PITA or PISA. The PITA or PISA deletion mutants were used in the reporter gene assays. Detection of the proteins’ expression is also shown. Error bars represent mean ± SD for three independent experiments. (**b–d**, **j**; mean and s.e.m., *n* = 3. *P < 0.05: two-tailed unpaired *t*-test). Unprocessed original scans of blots are shown in Supplementary information, Figure S9

We investigated whether specific members of the KZNF family negatively regulate p53 activity like Apak. We previously showed that Apak is a phosphorylation substrate of ATM kinase^10, 11^ and we have found that 48 members of the total 423 KZNF proteins contain potential ATM phosphorylation sites, the SQ/TQ motif. We cloned 43 of these members into expression vectors, and confirmed that they could drive ectopic expression. When we used reporter gene assays with an artificial pG13L luciferase construct, we identified 15 members (ZNF475, ZNF568, ZNF498, ZNF713, ZNF248, ZNF383, ZNF589, ZNF49, ZNF205, ZNF510, ZNF8, ZNF37, ZNF195, ZNF543, and ZNF202) that could significantly decrease the transcriptional activity of p53 (Supplementary information, Figure S1A). To determine which of these KZNF proteins specifically regulate metabolism-related target genes of p53, each of the fifteen *KZNF* genes was transfected into p53-wild type human colon cancer HCT116 cells, and endogenous protein levels of a series of p53 target genes were measured by western blot analysis. Notably, ZNF568 specifically downregulated the expression level of SCO2 and ZNF475 specifically downregulated the expression level of TIGAR (Fig. 1a and Supplementary information, Figure S1B). Increasing amounts of ZNF475 and ZNF568 gradually decreased the expression of TIGAR and SCO2, respectively, without significant effects on p53 levels in HCT116 cells (Supplementary information, Figure S1C). Similar results were observed in breast cancer MCF-7 cells and osteosarcoma U2OS cells (Supplementary information, Figure S1D). Based on these selective effects, we named ZNF475 as PITA (p53 inhibitor of TIGAR activation) and ZNF568 as PISA (p53 inhibitor of SCO2 activation) to clearly reflect their regulatory role.

PITA and PISA are highly conserved across species. Human PITA protein (407 amino acids) contains an N-terminal KRAB domain and 8 consecutive C2H2 zinc fingers. Similarly, human PISA protein (644 amino acids) contains a KRAB and 15 C2H2 zinc fingers. Immunofluorescence staining and cell fractionation analysis revealed that endogenous PITA and PISA were localized predominantly in the nucleus and secondarily in the cytoplasm in HCT116 cells (Supplementary information, Figure S1E and F). We also screened the expression spectrum of PITA and PISA in various tissues by immunohistochemical (IHC) staining. The results showed that PITA protein was highly expressed in adult brain, moderately in tongue, ileum, lung, skeletal muscle, artery and bladder. PISA protein was highly expressed in adult brain, heart and moderately in ileum, artery, lung and testis (Supplementary information, Figure S1G and H). Consistent with the IHC staining showing that PITA and PISA both express predominantly in the adult brain, GeneAtlas data also showed that PITA protein was highly expressed in adult brain, whereas PISA protein was highly expressed in many tissues including adult adrenal, brain, endometrium fat, kidney and ovary (Supplementary information, Figure S1I). To investigate the endogenous function of PITA and PISA, we carried out RNA interference using lentivirus to deliver short hairpin RNAs (shRNAs) into HCT116 cells. The knockdown efficiency of PITA or PISA was verified by both mRNA and protein expression analysis (Supplementary information, Figure S1J and K). Depletion of PITA or PISA significantly enhanced the transcription factor activity of p53 in HCT116 cells, whereas overexpression of PITA or PISA inhibited p53’s transcription factor activity (Fig. 1b and Supplementary information, Figure S1L). Similar results were obtained in MCF-7 and U2OS cells (Supplementary information, Figure S1M). Depletion of PITA caused a three-fold increase in mRNA expression of TIGAR without significant effects on other glycolysis-related genes (Fig. 1c and Supplementary information, Figure S1N). Similarly, depletion of PISA led to a five-fold increase in mRNA expression of SCO2 without significant effects on a subset of *FAO* genes, *OXPHOS* genes or nuclear mitochondrial genes (Fig. 1c and Supplementary information, Figure S1N). Consistent results were observed in MCF-7 and U2OS cells (Supplementary information, Figure S1O). The regulation of TIGAR by PITA and SCO2 by PISA was dependent on p53 because PITA or PISA expression were unable to regulate TIGAR and SCO2 in HCT116 p53^−/−^ cells, but it was rescued by the reintroduction of p53 (Fig. 1d). Furthermore, depletion of PITA or PISA disregulated the expression level of TIGAR or SCO2, respectively (Fig. 1e). Taken together, these results indicate that PITA and PISA are bona fide selective regulators of p53 activity.

To investigate the molecular mechanisms by which PITA and PISA regulate p53 activity, we assessed whether they could interact with p53 directly. Glutathione *S*-transferase (GST) pull-down assays *in vitro* and co-immunoprecipitation (Co-IP) assays from cell extracts showed that PITA and PISA interacted with p53 both *in vitro* and *in vivo* (Fig. 1f, g; Supplementary information, Figure S1P and Q). Deletion analysis showed that the C-terminal region (291–393 aa) of p53 mediated the binding to PITA and PISA (Fig. 1h). For PITA or PISA, the zinc-finger repeats rather than the KRAB domain are required for interaction with p53 (Fig. 1i). Both the KRAB domain and zinc fingers were required for PITA or PISA to repress p53 efficiently, although KRAB was not required for p53 binding (Fig. 1j). These results suggest that PITA and PISA regulate p53 activity through KRAB domain-associated mechanisms similar to that shown by Apak.^10, 11^

### PITA and PISA preferentially inhibit the binding of p53 to TIGAR and SCO2, respectively

**Fig. 2:**
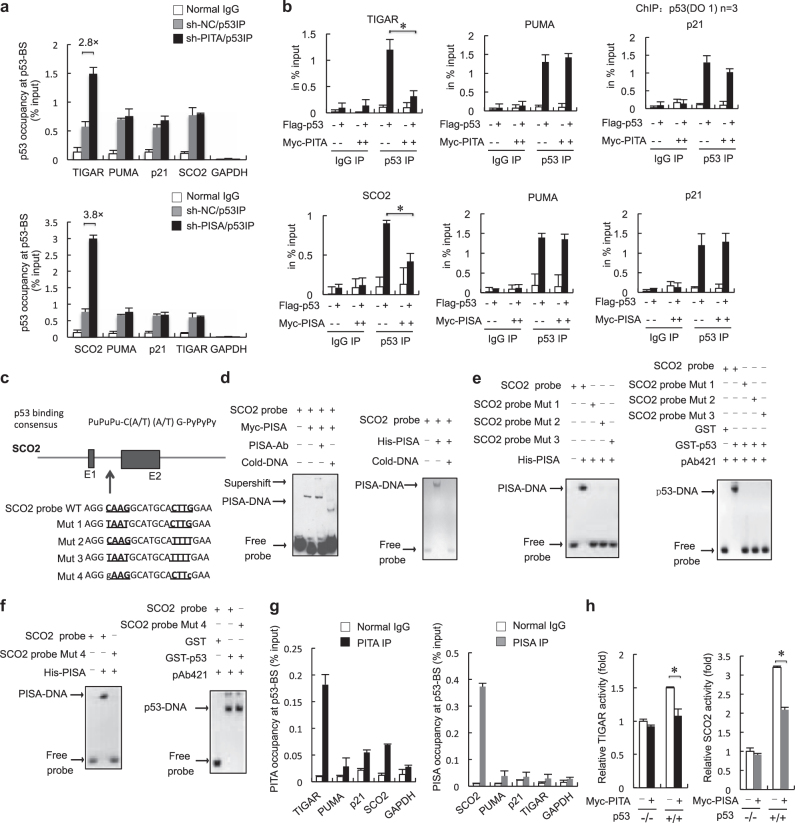
PITA and PISA preferentially inhibit the binding of p53 to the target genes TIGAR and SCO2, respectively. **a** ChIP assays in PITA-knocking down and PISA-knocking down or control p53^+/+^ HCT116 cells. IP immunoprecipitation. **b** Multiple ChIP analysis on the consensus binding sequences of p53 targets. HCT116p53^−/−^ cells were transfected with a mock vector, PITA, p53, or cotransfected with p53 and PITA as indicated (top), HCT116p53^−/−^ cells were transfected with a mock vector, PISA, p53, or cotransfected with p53 and PISA as indicated (bottom). After 24 h, ChIP was carried out using a control mouse IgG or an anti-p53 antibody, and RT-PCR was performed for the indicated promoters. **c** The p53-binding sequence in the promoter or intron of SCO2 is shown. For intron 1, the PISA-binding core sequence is underlined. PISA binds to the PISA/p53-BS of SCO2 *in vitro*. **d** Supershift EMSA using nuclear extracts derived from p53^−/−^ HCT116 cells harboring Myc-PISA. The PISA antibody and an unlabeled competitor probe were added as indicated. EMSA was performed with 100 ng of His-PISA and biotin-labeled PISA/p53-BS oligonucleotides. **e**, **f** EMSA of the p53 protein with PISA/p53-BS and mutants. The mutant PISA/p53-BS probes (for sequences, see **c**) were used in the EMSA with PISA protein. The mutant probes were used in the EMSA with the p53 protein and p53 antibody. EMSA was performed with 100 ng His-PISA and biotin-labeled mutant PISA/p53-BS. **g** ChIP assays in p53^+/+^ HCT116 cells. The promoter or intron of the indicated p53 target genes were amplified from immunoprecipitates of anti-PITA, anti-PISA, or IgG control. GAPDH was used as an internal control. **h** p53^−/−^ and p53^+/+^ HCT116 cells were co-transfected with TIGAR/p53-BS-Luc and PITA or SCO2/p53-BS-Luc and PISA, and the luciferase activity was measured. Error bars represent mean ± SD for three independent experiments. (**a**, **b**, **g**, **h**; mean and s.e.m., *n* = 3. *p < 0.05: two-tailed unpaired *t*-test). Unprocessed original scans of blots are shown in Supplementary information, Figure S9

To verify that PITA specifically regulates TIGAR and that PISA specifically regulates SCO2, we performed chromatin immunoprecipitation (ChIP) assays in HCT116 cells. These showed that PITA knockdown significantly augmented the binding of p53 to the intron of TIGAR (Fig. 2a, upper) and that PISA knockdown significantly augmented binding of p53 to the intron of SCO2 (Fig. 2a, lower), but not to pro-apoptosis genes or pro-arrest genes such as PUMA and p21. Western blot analysis confirmed that depletion of PITA or PISA had no significant effects on the protein levels of PUMA and p21 (Supplementary information, Figure S2A). Conversely, PITA or PISA overexpression decreased the binding of p53 to TIGAR or SCO2, respectively, in HCT116 p53^+/+^ cells (Supplementary information, Figure S2B). We confirmed by western blot analysis that overexpression of PITA or PISA also had no significant effects on the protein levels of PUMA and p21 (Supplementary information, Figure S2C). ChIP assays in MCF-7 and U2OS cells showed the same specificity for TIGAR or SCO2 as in HCT116 cells (Supplementary information, Figure S2D). Co-expression of PITA with p53 reduced the occupancy of p53 at the intron of TIGAR, and co-expression of PISA with p53 reduced the occupancy of p53 at the intron of SCO2 (Fig. 2b). These data suggest a model in which PITA or PISA binds to p53 and selectively interferes with its binding to aerobic glycolysis and mitochondrial respiration targets, that is to say TIGAR and SCO2, respectively.

To further investigate the mechanism of PITA and PISA, we performed electrophoresis mobility shift assays (EMSA) *in vitro* to measure the DNA-binding activities of p53 and PITA/PISA themselves to the *TIGAR* and *SCO2* genes. The p53-binding site in the SCO2 sequence is located within the first intron and consistent with the well-defined p53-binding consensus sequence, which comprises a PuPuPu-C(A/T)(A/T)G-PyPyPy element (Fig. 2c). This sequence was synthesized as a probe for EMSA assays in which we found that the SCO2 probe clearly bound to the nuclear extract expressing Myc-tagged PISA. Incubation with a PISA-specific antibody resulted in a supershift (Fig. 2d, left), confirming the binding specificity. This probe also supported the formation of a complex with purified His-PISA protein (Fig. 2d, right). Mutation of CAAG to TAAT (Mut1), CTTG to TTTT (Mut2) or both (Mut3) resulted in loss of the PISA/DNA complex (Fig. 2e, left). In addition, these mutations also abolished binding of the probe to p53 (Fig. 2e, right), suggesting that CxxG elements in SCO2 are required for both PISA and p53 binding. Furthermore, we identified another mutant of the SCO2 intron (Mut 4, in which CAAG and CTTG elements were mutated to GAAG and CTTC, respectively) that lost the binding ability to PISA, but retained binding to p53 (Fig. 2f). These results suggest that PISA and p53 bind to the SCO2 intron through overlapping, but not identical sequences. Therefore, PISA might compete with p53 in direct binding to the *SCO2* gene. ChIP assays revealed that PISA co-immunoprecipitated with the SCO2 intron 1 with a high affinity in HCT116 cells; in contrast, it showed very-weak affinity to PUMA, p21 or TIGAR (Fig. 2g, right).

We next analyzed the regulation of PITA on the *TIGAR* gene. The *TIGAR* gene contains six potential coding exons and two possible p53-binding sites (BS), one upstream of the first exon (BS1) and one within the first intron (BS2) (Supplementary information, Figure S2E). We found that PITA reduced the DNA-binding activity of p53 to its binding sites on TIGAR (Supplementary information, Figure S2F, left). However, TIGAR probes comprising p53-binding sites (TIGAR/p53-BS1 and BS2) did not bind to the nuclear extract expressing Myc-tagged PITA or purified-His-PITA protein (Supplementary information, Figure S2F, right and G, left). We also attempted to identify PITA-binding sequences in more probes (BS3 and BS4), but failed to find any sites (Supplementary information, Figure S2G, right). Interestingly, ChIP assays revealed that PITA co-immunoprecipitated with the TIGAR intron with a modest affinity in p53^+/+^ HCT116 cells. Compared to PISA binding to SCO2, PITA showed a weaker affinity for TIGAR (Supplementary information, Fig. 2G, left). We obtained similar results in MCF-7 and U2OS cells (Supplementary information, Figure S2H). These results suggest that the regulatory mechanism of PITA on TIGAR is not identical to that of PISA on SCO2. PITA regulates TIGAR possibly by binding to the p53 protein and inhibiting its binding to TIGAR sequences.

To evaluate the functional relevance of the DNA-binding activity of PITA or PISA, we performed reporter gene assays with the heterologous luciferase gene fused to one upstream copy of TIGAR/p53-BS or SCO2/p53-BS in the pGL3 vector. Overexpression of PITA repressed p53-mediated TIGAR/p53-BS-luc activity, and overexpression of PISA repressed p53-mediated SCO2/p53-BS-luc activity (Fig. 2h, and Supplementary information, Figure S2I). Using a reporter assay with TIGAR and SCO2 intron mutant-driven luciferase, we found that PITA and PISA overexpression had no significant effects on the mutant reporter activity (Supplementary information, Figure S2J). To further investigate PITA-binding sites on TIGAR, we performed PITA ChIP-seq assays in HCT116 p53^−/−^ cells. We displayed the resulting genomic PITA data, by applying Circos, a visualization tool, to the established use of circular maps. The distribution of all peaks across the genome is shown in Supplementary information, Figure S2K and Table S1. To analyze the genes immunoprecipitated with PITA and identify their biological themes, we used the DAVID functional annotation tool to annotate and extract gene groups that displayed significant (*P* < 0.05) enrichment of Gene Ontology (GO) and KEGG. We found that these DEGs were enriched for GO terms reflecting multiple biological processes, molecular functions and signaling pathways of PITA in HCT116 p53-deficient cells (Supplementary information, Table S2, Figure S2L and M). Gene annotation of PITA-binding sites, using a false discovery rate of 0.1, showed the PITA-potential-binding motifs in Supplementary information, Figure S2N. Together, these analyses indicate that PITA and PISA are novel selective regulators of p53 target genes with metabolism-related functions.

### PITA and PISA regulate aerobic glycolysis and mitochondrial respiration

**Fig. 3:**
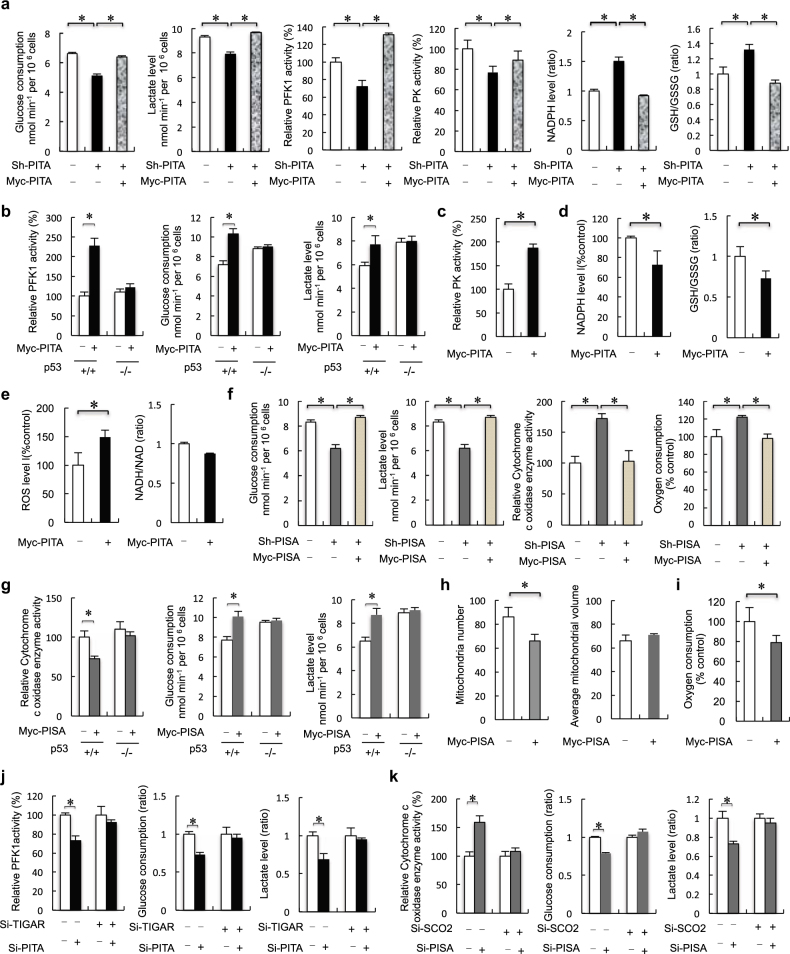
PITA and PISA regulate aerobic glycolysis and mitochondrial respiration. **a** Analysis of glucose consumption and lactate levels, PFK1 activity, F2,6BP activity, PK activity, NADPH levels and GSG/GSSH ratio in PITA-depleted or reintroduction of PITA HCT116 p53^+/+^ cells. **b** Analysis of PFK1 activity, glucose consumption and lactate levels in ectopic PITA-overexpressing HCT116 p53^+/+^ and HCT116 p53^−/−^ stable transfectants. **c** PK activity in HCT116 p53^+/+^ cells stably transfected with PITA. **d**, **e** NADPH levels, GSG/GSSH (ratio) levels, NADH/NAD (ratio) and ROS levels in overexpression of PITA HCT116 p53^+/+^ stable cell lines. **f** Analysis of glucose consumption and lactate levels, cytochrome c oxidase (COX) activity, in PISA-depleted or reintroduction of PISA HCT116 p53^+/+^ cells. Oxygen consumption (mean TSD, nmol min^−1^ mg^−1^ protein) was measured by a Clark type oxygen microelectrode in PISA-depleted HCT116 p53^+/+^ cell lines mitochondria preparations. **g** Cytochrome c oxidase (COX) activity, glucose consumption and lactate levels, in overexpression of PISA HCT116 p53^+/+^ or HCT116 p53^−/−^ cell lines. **h** Analysis of total and single mitochondrial volume and mitochondrial numbers as described in the Experimental Procedures section in overexpression of PITA HCT116 p53^+/+^ stable cell lines. **i** Oxygen consumption (mean TSD, nmol min^−1^ mg^−1^ protein) was measured by a Clark type oxygen microelectrode in overexpression of PISA HCT116 p53^+/+^ cell lines mitochondria preparations. **j** After knockdown of TIGAR, PFK1 activity, glucose consumption and lactate levels in PITA-depletion HCT116 p53^+/+^ and HCT116 p53^−/−^ cells. **k** After knockdown of SCO2, COX activity, glucose consumption and lactate levels in PISA-depletion HCT116 p53^+/+^ and HCT116 p53^−/−^ cells. Error bars represent mean ± SD for three independent experiments. (**a**–**k**; mean and s.e.m., *n* = 3. *P < 0.05: two-tailed unpaired *t*-test)

To investigate whether PITA modulates glycolytic rate, we compared glucose consumption and lactate production. This revealed that knockdown of PITA led to decreased glucose consumption and lactate production in HCT116 cells (Fig. 3a). TIGAR acts as a phosphatase upon fructose-2, 6-bisphosphate (F2, 6BP), and thereby decreases the activity of PFK1. PITA significantly inhibited the expression of TIGAR, suggesting that PITA might specifically enhance the activity of PFK1. As expected, we found that knockdown of PITA decreased the activity of PFK1 (Fig. 3a). The major control step in glycolysis is through PFK1, but hexokinase 2 (HK2) and pyruvate kinase (PK) are additional control sites. We found that knockdown of PITA decreased PK activity, but only had modest effects on HK2 activity in HCT116 cells (Fig. 3a; Supplementary information, Figure S3A). Suppressing glycolysis via inhibition of PFK1 could lead to accumulation of glycolytic intermediates, and might redirect metabolic flux down the oxidative pentose phosphate pathway (PPP). This shift would provide cells with pentose sugars for nucleotides and nucleic acid biosynthesis, as well as reducing equivalents from NADPH to combat oxidative stress.^13^ Knockdown of PITA was associated with increased NADPH levels (Fig. 3a). Flux through the PPP generates NADPH, which maintains a pool of reduced glutathione (GSH) and plays an important role in combating ROS-mediated cell death.^4, 14^ Consistent with increased NADPH, knockdown of PITA resulted in a 40% increase in the ratio of reduced oxidized glutathioine (GSH/GSSG ratio), a readout of redox status (Fig. 3a). Reintroduction of PITA rescued the effects on knockdown of PITA. Conversely, overexpression of PITA-enhanced PFK1 activity, glucose consumption and lactate production in HCT116 p53^+/+^ cells. However, these effects were not observed in HCT116 p53^−/−^ cells (Fig. 3b) suggesting that PITA regulates glucose metabolism according to p53 status. Enhancing PITA level also promoted PK activity (Fig. 3c). Overexpression of PITA was associated with decreased NADPH levels, resulting in a 30% reduction in the GSH/GSSG ratio and increasing ROS levels, but had no significant effect on the NADH/NAD ratio (Fig. 3d, e). Furthermore, knockdown of PITA led to decreased glucose consumption, lactate production and PFK1 activity in MCF-7 and U2OS cells (Supplementary information, Figure S3B), confirming the regulatory function of PITA in cell metabolism of multiple types of cells.

SCO2, a key component of OXPHOS required for assembly of the cytochrome c oxidase complex (complex IV in the mitochondrial electron transport chain (ETC) and a major site of oxygen consumption in the mitochondria), is transcriptionally activated by p53. PISA had significant inhibitory effects on SCO2, suggesting that PISA might affect the activity of COX (cytochrome c oxidase). Indeed, knockdown of PISA decreased glucose consumption, lowered lactate production and increased COX activity in HCT116 p53^+/+^ cells (Fig. 3f). Reintroduction of PISA rescued the effects on knockdown of PISA. Additionally, knockdown of PISA decreased glucose consumption, lowered lactate production and increased COX activity in MCF-7 and U2OS cells (Supplementary information, Figure S3C). We also found induction (25%) of cellular oxygen consumption after knockdown of PISA in HCT116 p53^+/+^ cells (Fig. 3f). Overexpression of PISA decreased COX activity and increased glucose consumption and lactate production in HCT116 p53^+/+^ cells, but not in p53-deficient cells (Fig. 3g). These results suggest that PISA inhibits p53-mediated mitochondrial respiration. Overexpression of PISA decreased levels of the electron transport chain protein cytochrome c and the protein subunits of complexes IV (Supplementary information, Figure S3D). PISA also decreased mitochondria number, but had no effect on the average mitochondrial volume (Fig. 3h). We also measured the activities of representative mitochondrial enzymes and PFK1 activity and found their activities similar in cells with or without overexpression of PISA (Supplementary information, Figure S3E). Furthermore, we confirmed a similar reduction (20%) of cellular oxygen consumption in PISA-overexpressing cells (Fig. 3i). To determine whether KZNF proteins specifically regulate the target genes of p53, each *KZNF* gene was transfected into HCT116 p53^+/+^ cells, and the endogenous expression levels of a series of reported p53 target genes were measured by Q-PCR analysis. The p53 target genes included the DNA damage-repair-related genes *p53R2* and *DDB2*, the drug resistance gene *MDR*, the angiogenesis-related gene *Tsp1* and the self-regulation-related gene *HDM2*. PITA and PISA had only weak effects on the expression levels of these target genes (Supplementary information, Figure S3F). Additionally, PITA promoted activity of PFK1 and modulated the glycolytic rate dependent on the presence of TIGAR (Fig. 3j and Supplementary information, Figure S3G). On the other hand, PISA decreased COX activity and modulated glycolytic activity dependent on expression of SCO2 (Fig. 3k and Supplementary information, Figure S3H). Among the cancer cell lines examined, PITA and PISA were highly expressed in HCT116 cells and poorly expressed in others (Supplementary information, Figure S3I, upper). Although we detected glycolytic activity in these cancer cell lines, we did not establish a link between expression of PITA or PISA and glycolysis/mitochondrial activity (Supplementary information, Figure S3I, lower).

### PITA promotes aerobic glycolysis in vivo

**Fig. 4:**
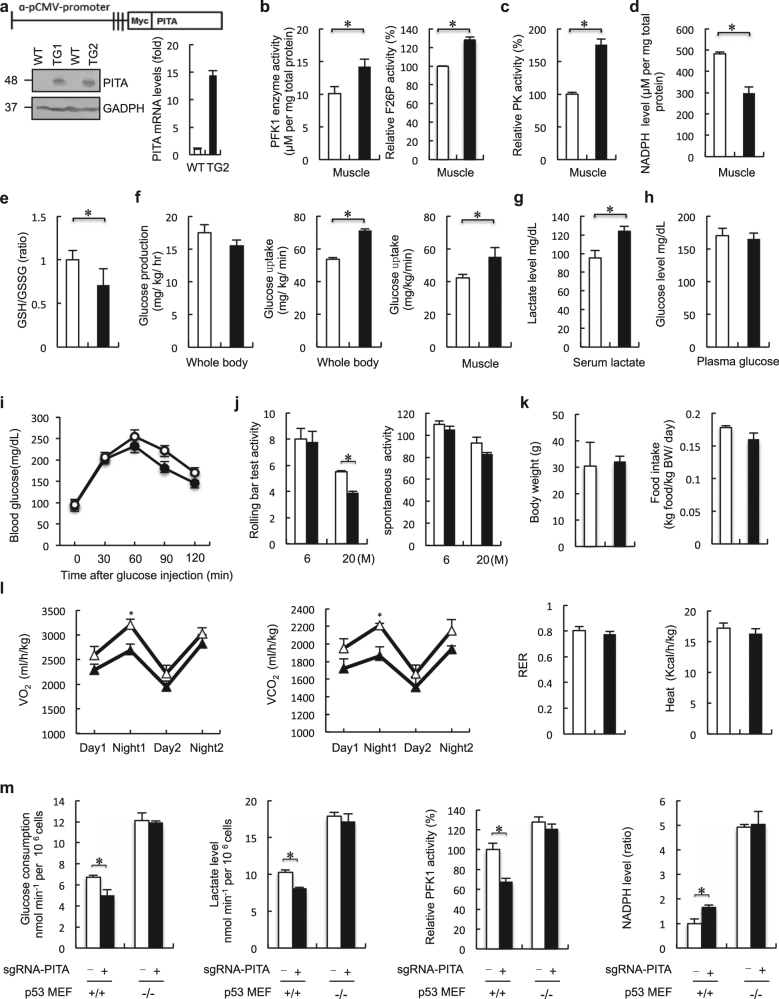
PITA regulates PFK1 activity and promotes aerobic glycolysis in vivo. **a** Schematic representation of the transgenic construct used to generate PITA transgenic (TG) mouse lines. PITA protein levels in WT and PITA TG mice at 8 weeks were assessed with both anti-Myc and anti-GAPDH antibodies. **b**, **c** Analysis of PFK1, F2,6BP and PK activity in muscle tissues from WT (*n* = 8) and PITA TG mice (*n* = 6) maintained on a normal diet. **d** NADPH levels in muscle of WT (*n* = 8) and PITA TG mice (*n* = 6) maintained on a normal diet. **e** GSG/GSSH (ratio) levels in plasma from WT (*n* = 8) and PITA TG mice (*n* = 6) maintained on a normal diet. **f** Following an overnight fast, WT (*n* = 8) and PITA TG mice (*n* = 6) were subjected to a hypereuglycemic clamp. Basal glucose production, whole-body and muscle glucose uptake rate during hypereuglycemic clamp, muscle (soleus and gastrocnemius) glucose uptake during the hypereuglycemic clamp. **g**, **h** Serum lactate levels and plasma glucose in WT (*n* = 8) and PITA TG mice (*n* = 8) mice in the fasted overnight states. **i** Glucose tolerance test on WT (opened circle, *n* = 7) and PITA TG mice (filled circle, *n* = 7) mice. **j** Left: Effect of walnut extract on rolling bar test time of WT (*n* = 7) and PITA (*n* = 7) mice. Right: Effect of walnut extract on locomotors time of WT and PITA mice. **k** Body weight of 3-month-old WT (*n* = 7) and PITA TG (*n* = 7) mice fed a normal chow. PITA TG mice have similar food intake (*n* = 7) in comparison with WT mice (*n* = 7). **l** Indirect calorimetry of WT (white triangle) and PITA TG mice (black triangle). Oxygen consumption (VO_2_), carbon dioxide release (VCO_2_), respiratory exchange ratio (RER; VCO_2_/VO_2_) and energy expenditure per kg of body weight were determined in WT and PITA TG mice in metabolic chambers (*n* = 8 per genotype). **m** Analysis of glucose consumption and lactate levels, PFK1 activity, NADPH level in CRISPR-Cas9 PITA MEF p53^+/+^ and MEF p53^−/−^cells. Data are means ± SD (*n*=3). Error bars represent mean ± SD for three independent experiments. (mean and s.e.m., *n* = 3. *P < 0.05: two-tailed unpaired *t*-test). Unprocessed original scans of blots are shown in Supplementary information, Figure S9. Data in **b–l** represent mean ± s.e.m. *P < 0.05: two-tailed unpaired *t*-test

To elucidate the physiological role of PITA in vivo, we generated transgenic (TG) mice expressing PITA from the CMV promoter (Fig. 4a). We detected increased PITA levels in all adult tissues of these mice as expected with TIGAR protein levels being downregulated in these tissues, but with no significant effects upon p53 expression (Supplementary information, Figure S4A). We compared PFK1 activity in various tissues from PITA WT and TG mice. Tissues from PITA TG mice, including muscle, exhibited substantially elevated PFK1 activity and F2,6BP compared with those in corresponding tissues from WT mice (Fig. 4b and Supplementary information, Figure S4B). By contrast, no significant difference in HK2 activity was observed between WT and PITA TG mouse muscle (Supplementary information, Figure S4C). PFK1 was also increased in the muscle of PITA TG mice (Supplementary information, Figure S4D). One of the central components in metabolic switch is the enzyme responsible for converting phosphoenol pyruvate into pyruvate, pyruvate kinase (PK). Decreased PK activity resulted in accumulation of glycolytic precursors for production in redox power and biomass.^15^ Increased PK activity, suggesting altered activity of this glycolytic regulatory node may contribute to the observed metabolic changes in PITA TG mice (Fig. 4c). In addition, we observed no significant difference in pyruvate dehydrogenase kinase (PDK) expression or activity between the muscle of WT and PITA TG mice (Supplementary information, Figure S4E and F). Furthermore, in tissues with sufficient NADPH levels for detection, PITA TG mice showed decreased NADPH levels in muscle, but no significant differences in other tissues (Fig. 4d and Supplementary information, Figure S4G). Consistent with the decreased NADPH, PITA TG mice displayed a 30% reduction in the serum GSH/GSSG ratio (Fig. 4e). One key characteristic of cancer cells is glucose utilization, which switches from energy-efficient oxidative phosphorylation to inefficient lactate production (the Warburg effect), allowing the accumulation of glycolytic intermediates and their redirection to biosynthetic pathways. To study whole-body and tissue glucose metabolism in detail, we performed hyperglycemic clamp analyses. Basal endogenous glucose production was comparable between PITA TG mice and WT mice. Glucose uptake rate in the whole body during clamp analysis was increased by 35% in TG mice relative to controls (Fig. 4f). In skeletal muscle, glucose uptake measured at the end of the clamp was elevated in TG mice (Fig. 4f). Next, we evaluated serum lactate levels as an indicator of glycolytic activity; we observed an induction in lactate levels of PITA TG mice compared to WT mice (Fig. 4g). Additionally, we found that glucose consumption and lactate production were increased in the MEF cells from PITA TG mice (Supplementary information, Figure S4H). Under fasting conditions, PITA TG mice displayed comparable plasma glucose levels to the control group (Fig. 4h). In a glucose tolerance test, the TG mice had similar fasting blood glucose levels and improved glycemic control compared to WT mice (Fig. 4i). These data show that PITA TG mice take up more glucose and direct a lower proportion of glycolytic products into mitochondrial oxidative phosphorylation. These features could indicate Warburg status, which is correlated with increased proliferation and a cancer-promoting state.

To evaluate the effect of PITA on exercise-induced adaptations, both WT and PITA TG mice were subjected to voluntary exercise training. WT and TG mice had no significant difference in spontaneous activity. However, in the rolling bar tests, aged (20-month-old) TG mice ran shorter distances than WT mice (Fig. 4j). The 20-month-old WT mice exhibited an increased heart weight-to-body weight ratio (data not shown), an expected consequence of endurance training. Next, we detected mitochondrial function and ATP in PITA mouse muscle. The average mitochondrial volume was decreased, whereas the mitochondrial number was unaffected in the TG muscle (Supplementary information, Figure S4I). The total ATP activity was similar between WT and TG mouse muscle (Supplementary information, Figure S4J). We also measured succinate deghydrogenase (SDH) activity, which is related to oxidation, and the enzyme activity of isocitrate dehydrogenase (IDH) and PHOSP, which are involved in fermentation and phosphorylation, in the muscle. No significant changes were observed (Supplementary information, Figure S4K). Our analysis of PITA TG mice revealed a modest increase in body weight and similar food intake (Fig. 4k). Despite a comparable food intake when normalized to body weight, magnetic resonance imaging (MRI) revealed that the PITA mice had increased fat mass and decreased lean content. When the total fat mass was normalized to the body weight for each mouse, PITA TG mice had a 20% induction in fat mass. In contrast, when normalized to the total body weight, no difference regarding the liver was observed between WT and PITA TG mice (Supplementary information, Table S3). Energy balance reflects energy intake, as measured by food intake, and energy expenditure. Compared to WT mice, PITA TG mice had lower oxygen consumption (VO_2_) and carbon dioxide production (VCO_2_) in both light and dark phases. Consistent with the lower O_2_ consumption, PITA TG mice generated slightly less heat than WT mice (Fig. 4l). Food intake was similar in PITA TG mice, indicating that the difference in energy expenditure observed between WT and PITA TG mice is not due to the changes in food intake, but due to a lowered metabolic state. We also used the CRISPR-Cas9 method to generate PITA-knockout mouse embryonic fibroblasts (MEFs) in both a p53^+/+^ and p53^−/−^ background. Endogenous PITA was depleted in MEF cells by gRNA with three independent target sites. We designed three sequence-specific single-guide RNA (sgRNA) target sites, which are 119 bp apart in the PITA sequence. They were ligated into three sgRNA expression cassettes of a Cas9 binary vector (Supplementary information, Figure S4L and M). We found that knockout of PITA led to decreased glucose consumption and lactate production in p53^+/+^ cells (Fig. 4m). We also found that knockout of PITA decreased the activity of PFK1 (Fig. 4m). However, this effect was not observed in MEF p53^−/−^ cells (Fig. 4m), suggesting that PITA regulates glucose metabolism dependent on the p53 status. Taken together, these results in mice fibroblast cells confirmed the conclusions obtained in human cells.

### PISA dampens mitochondrial respiration in vivo

**Fig. 5:**
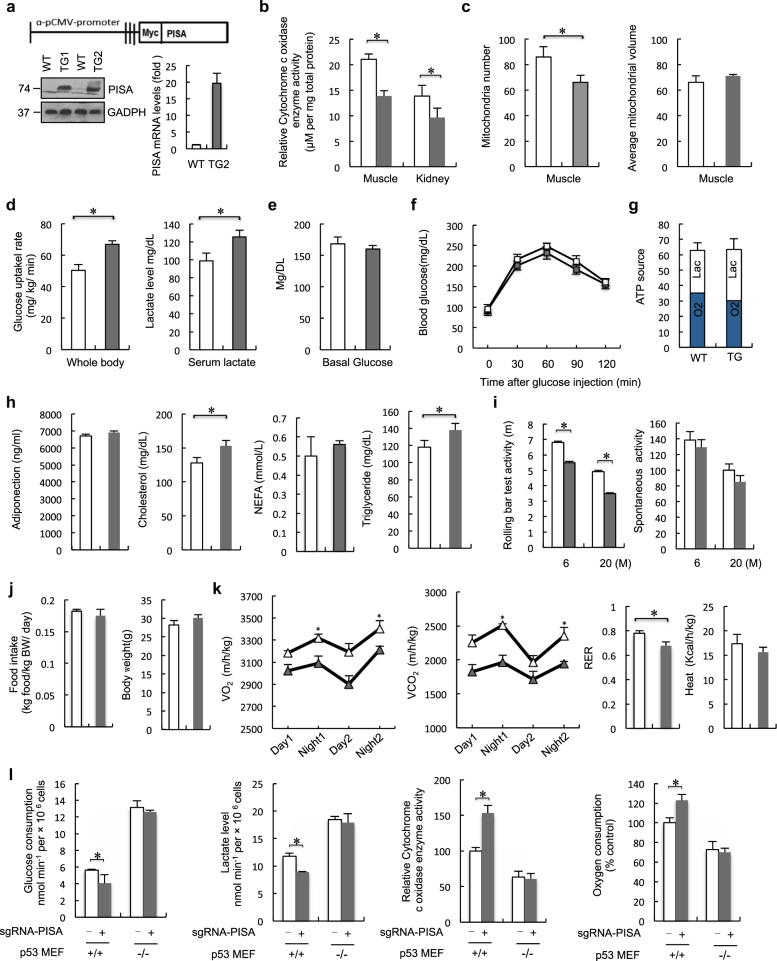
PISA regulates cytochrome c oxidase activity and dampens mitochondrial respiration in vivo. **a** Schematic representation of the transgenic construct used to generate PISA transgenic mouse lines. PISA protein levels in WT and two independent lines of PISA TG mice at 8 weeks were assessed with anti-Myc antibodies. **b** Cytochrome c oxidase (COX) activity in tissues from WT (*n* = 8) and PISA TG mice (*n* = 6) maintained on a normal diet. **c** Analysis of total and single mitochondrial volume and mitochondrial numbers (WT *n* = 8 and PISA TG mice *n* = 6). **d** Following an overnight fast, WT (*n* = 8) and PISA TG mice (*n* = 6) were subjected to a hypereuglycemic clamp. Whole-body glucose disposal rate during hypereuglycemic clamp. Serum lactate levels in the fasted overnight states. **e** Plasma glucose levels in WT (*n* = 8) and PISA TG mice (*n* = 6) mice in the fasted overnight states. **f** Glucose tolerance test on WT (opened circle, *n* = 7) and PISA TG mice (filled circle, *n* = 7) mice. **g** The amount of ATP (mean TSD, nmol min^−1^ mg^−1^ protein) produced by aerobic respiration (gray bars, O2) and glycolysis (light bars, Lac) was calculated by measuring oxygen consumption and lactate production in the mice muscle mitochondria preparations. **h** Plasma adiponectin, cholesterol, NEFA and triglyceride levels in WT and PISA TG mice in the fasted overnight states. **i** Left: Effect of walnut extract on rolling bar test time of WT and PISA TG mice. (*n* = 7 per group); Right: effect of walnut extract on locomotors time of WT and PISA TG mice (*n* = 7 per group). **j** Body weight of 3-month-old WT (*n* = 8) and PISA TG (*n* = 6) mice fed a normal chow. PISA TG mice have similar food intake in comparison with WT mice. **k** Indirect calorimetry of WT (white triangle) and PISA TG mice (gray triangle). Oxygen consumption (VO_2_), carbon dioxide release (VCO_2_), respiratory exchange ratio (RER; VCO_2_/VO_2_) and energy expenditure per kg of body weight were determined in WT and PISA TG mice in metabolic chambers (*n* = 8 per genotype). **l** Analysis of glucose consumption and lactate levels, COX activity, oxygen consumption in CRISPR-Cas9 PISA MEF p53^+/+^ and MEF p53^−/−^cells. Error bars represent mean ± SD for three independent experiments. (mean and s.e.m., *n* = 3. *P < 0.05: two-tailed unpaired *t*-test). Unprocessed original scans of blots are shown in Supplementary information, Figure S9. Data in **b–k** represent mean ± s.e.m. *P < 0.05: two-tailed unpaired *t*-test

We also generated mice expressing PISA from the CMV promoter (PISA TG mice) to investigate the physiological role of PISA (Fig. 5a). We found that SCO2 protein level was downregulated in TG mice compared with the WT mice, while the p53 expression level was comparable (Supplementary information, Figure S5A). Tissues from PISA TG mice, including muscle and kidney, exhibited decreased COX enzymatic activity compared with those in corresponding tissues from WT mice (Fig. 5b and Supplementary information, Figure S5B). The mitochondrial number in muscles of TG mice was also decreased, while the average mitochondrial volume was unaffected (Fig. 5c). The decrease in mitochondrial number may be related to the ability of PISA to reduce mitochondrial function. PISA TG mice exhibited increased glycolysis and lactate production (Fig. 5d and Supplementary information, Figure S5C). The TG mice displayed normal plasma glucose levels and glycemic excursions in a glucose tolerance test (Fig. 5e, f). The total amount of ATP was similar in WT mice and PISA TG mice, but the PISA TG mice produced higher levels of lactate, indicating a change in the mode of energy production to one that favors glycolysis (Fig. 5g). FAO (fatty acid oxidation) represents a crucial process in energy metabolism and fat storage. Fatty acids can either be used for lipid synthesis and protein modification or they can be degraded through mitochondrial β-oxidation, which produces substrates that maintains ATP generation through oxidative phosphorylation.^15^ To assess whether PISA mice use lipids as energetic substrates, we examined adiponectin, triglycerides, cholesterol and non-esterified fatty acid (NEFA) levels in both WT and TG mice. Both the plasma cholesterol and triglyceride levels were increased in PISA TG mice compared to WT mice, while the adiponectin and NEFA levels were comparable (Fig. 5h). We did not find significant differences in the rate of FAO between WT and PISA TG embryonic fibroblasts (MEFs) (Supplementary information, Figure S5D). We next examined the use of glucose as a precursor for lipid synthesis and found that PISA TG cells showed a modest increase in the contribution of (6–14C)-glucose to lipid synthesis compared to WT cells (Supplementary information, Figure S5E).

Since the mitochondrial function in the muscle of PISA TG mice differed from WT, we investigated whether there was also a difference in the level of physical activity. To examine this hypothesis, we assessed the rolling bar test activity as well as the spontaneous activity of animals. Both 6-month and 20-month PISA TG mice exhibited lower rolling bar test activity compared with WT mice, while no significant difference was observed in spontaneous activity (Fig. 5i). These mice showed a similar body weight and food intake (Fig. 5j). MRI revealed that the PISA mice have an increased fat mass and a decreased lean content. When the total fat mass was normalized to the body weight for each mouse, PISA TG mice had a 20% induction in fat mass. In contrast, when normalized to the total body weight, no difference regarding the liver was observed between WT and PISA TG mice (Supplementary information, Table S3). Furthermore, indirect calorimetric analysis revealed that PISA TG mice presented significantly lower energy expenditure than WT mice (Fig. 5k), indicating that the PISA TG mice have low rates of energy expenditure due to a reduction in mitochondria (oxidative phosphorylation). These data suggest that the decrease in energy expenditure observed in PISA TG mice could reflect decreased mitochondrial oxidative phosphorylation. We also generated PISA knockout p53^+/+^ and p53^−/−^ MEFs with CRISPR-Cas9 (Supplementary information, Figure S5F and G). Knockout of PISA increased COX activity and decreased glucose consumption and lactate production in MEF p53^+/+^ cells, but not in the p53-deficient MEF cells (Fig. 5l). Together, these analyses of mouse fibroblasts confirmed the conclusion obtained in human cell lines.

### PITA and PISA dissociate from p53 upon glucose starvation, which requires ATM-mediated phosphorylation

**Fig. 6:**
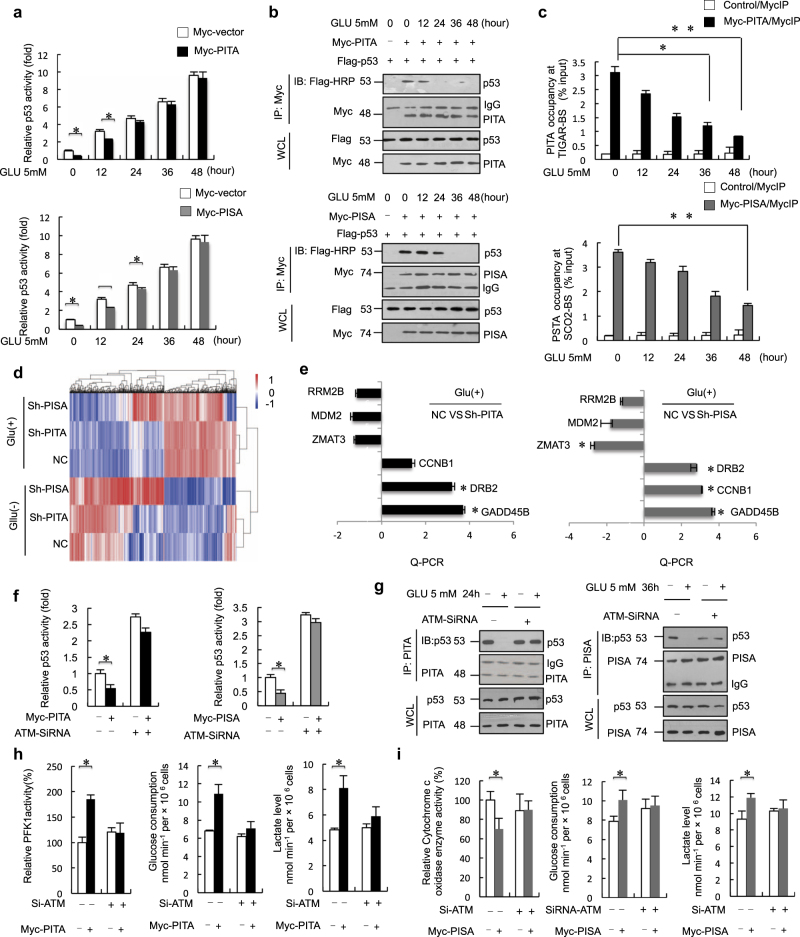
PITA and PISA dissociation from p53 upon glucose starvation requires ATM-mediated phosphorylation. **a** pG13-Luc was cotransfected into HCT116 p53^+/+^cells with p53 and PITA or PISA as indicated. After 24 h, glucose starvation (low dosage, 5 mM) was carried out for the indicated times followed by luciferase assay. **b** Co-immunoprecipitation of exogenous PITA with p53 or co-immunoprecipitation of exogenous PISA with p53 in HCT116 p53^−/−^ cells were treated with glucose starvation was carried out for indicated times. Exogenous PITA–p53 and PISA-p53 interactions were analyzed. **c** ChIP assays were performed to amplify the *TIGAR* or *SCO2* gene. HCT116 p53^+/+^ cells were transfected with Myc-PITA or Myc-PISA and treated with glucose starvation. **d** Sh-NC, Sh-PITA and Sh-PISA of HCT116 p53^+/+^ cells with or without glucose starvation were analyzed RNA sequencing, and differentially expressed genes are shown as a heatmap (ordered by *P* values in Supplementary information, Tables S5 and S6). **e** qRT-PCR analysis of selected genes associated p53 signaling in PITA or PISA knockdown of HCT116 p53^+/+^ cells on glucose starvation (5 mM). **f** Effect of ATM knockdown on PITA or PISA activity in ATM wild-type cells. **g** HCT116 p53^+/+^ cells were transfected with ATM siRNA or control siRNA and treated with GLU-5 mM. Endogenous PITA–p53 interactions or PISA-p53 were analyzed. **h** After knockdown of ATM, PFK1 activity, glucose consumption and lactate levels, NADPH levels and GSG/GSSH (ratio) levels in overexpression of PITA HCT116 p53^+/+^ cells. **i** COX activity, glucose consumption and lactate levels in knockdown of ATM HCT116 p53^+/+^ cells. Error bars represent mean ± SD for three independent experiments. (**c**, **e**, **f**, **h**; mean and s.e.m., *n* = 3. *P < 0.05: two-tailed unpaired *t*-test). Unprocessed original scans of blots are shown in Supplementary information, Figure S9

Because p53 is a key activator in transcriptional regulation of cellular metabolism and lies at a signaling node in response to diverse cellular stresses, we investigated the effect of PITA and PISA on transcriptional activity of p53 under metabolic stress conditions. Glucose starvation is known to induce p53 protein levels.^16^ As p53 downregulates glycolysis through TIGAR and upregulates oxidative phosphorylation through SCO2, p53 might fail to antagonistically regulate glycolysis and oxidative phosphorylation under identical conditions of cellular stress.^17^ Recently, p53 was shown to specifically regulate the *TIGAR* gene in cancer cells exposed to low doses of cellular stress and the *SCO2* gene in cancer cells exposed to high doses of cellular stress.^18, 19^ We then determined whether p53-mediated regulation of TIGAR and SCO2 is dependent upon the degree of cellular stress. The inhibitory activity of PITA was markedly decreased at 24–36 h, while that of PISA was observed for extended periods upon starvation treatment (36–48 h) of HCT116 p53^+/+^ cells (Fig. 6a). We also examined the inhibitory activity of PITA or PISA in HCT116 p53^+/+^ cells cultured in medium supplemented with different concentrations of glucose (1.5, 2.5, 5, 12.5 and 25 mM). HCT116 p53^+/+^ cells were maintained in different concentrations of glucose for 18 h after transfection of PITA or PISA. The inhibitory activity of PITA was markedly decreased at 1.5, 2.5 and 5mM (concentration indicated in culture medium), whereas that of PISA was observed at 1.5 and 2.5 mM concentrations of glucose with HCT116 p53^+/+^ cells (Supplementary information, Figure S6A). Protein-protein interaction assays showed that PITA dissociated from p53 at the corresponding time points (24–36 h), while PISA dissociated from p53 later than PITA (36–48 h) (Fig. 6b). We also showed that some DNA damage signals, including H_2_O_2_, etoposide and cisplatin treatment, resulted in dissociation of PITA from p53. Etoposide and cisplatin treatment resulted in dissociation of PISA from p53 (Supplementary information, Figure S6B). Then, we investigated the dynamic alterations of p53-interacting proteins upon glucose starvation. We purified p53 proteins in HCT116 p53^+/+^ cells through immunoprecipitation at different time points after glucose starvation (0, 24 and 48 h) and then performed mass spectrometry analysis. Screening was performed by bioinformatics analysis, and 412 total potential p53-interacting proteins were identified, including casein kinase 1,^20^ a known p53-binding protein and 4 KZNFs, including ZNF475/PITA, ZNF568/PISA, ZNF8 and ZNF195, in the unstressed cells (Supplementary information, Figure S6C and Table S4). At 24 h and 48 h after starvation, we found that the components interacting with the p53 complex changed. Interaction between PITA and p53 was significantly reduced at early time points (24 h), but PISA interactions were reduced at extended periods of starvation treatment (48 h) compared to unstressed cells. Interactions between p53 and ZNF195 or ZNF8 were not affected under glucose starvation conditions at 24 h and 48 h. Interestingly, ZNF248 was specifically identified at starvation for 24 h, and ZNF498 was identified at 48 h compared to unstressed cells.

We also measured the DNA-binding activity of p53 to introns of p53 target genes in response to glucose starvation. We investigated the cooperation of PITA with TIGAR and PISA with SCO2 in response to glucose starvation at different time points. ChIP assays confirmed that PITA dissociated from TIGAR at early time points under glucose starvation conditions (Fig. 6c, upper), and PISA dissociated from SCO2 at later time points (Fig. 6c, lower). Taken together, these data demonstrate that PITA and PISA successively dissociate from p53 to promote transactivation of its glycolytic target gene TIGAR at early time points of starvation and the mitochondrial respiration target gene *SCO2* at later time points of starvation. We also measured the DNA-binding activity of p53 to promoters/introns of p53 target genes in response to different concentrations of glucose. ChIP assays confirmed that PITA dissociated from TIGAR on glucose concentrations of 1.5, 2.5, and 5 mM, and PISA dissociated from SCO2 on glucose of 1.5 and 2.5 mM (Supplementary information, Figure S6D). After shRNA knockdown of PITA, we found that cells were much more sensitive to hypoxia and H_2_O_2_ (Supplementary information, Figure S6E and F). Collectively, the above data show that PITA impaired the ability of cells to respond to oxidative stress.

Furthermore, to determine the general effect of depleting PITA or PISA in HCT116 cells in response to glucose starvation, we performed RNA-seq in control, PITA-depleted and PISA-depleted HCT116 p53^+/+^ cells with or without glucose starvation. By comparing libraries from control and PITA-depleted, or control and PISA-depleted in response to glucose starvation, we identified a significant number of DEGs including 213 upregulated and 212 downregulated genes in PITA-depleted cells; 382 upregulated and 233 downregulated genes in PISA-depleted cells (Fig. 6d; Supplementary information, Figure S6G, Tables S5 and 6). Further analysis with Gene Ontology (GO) and KEGG pathway indicated that these DEGs were enriched for GO terms of multiple biological processes, molecular functions and signaling pathways in PITA-depleted and PISA-depleted cells (Supplementary information, Figure S6H), and KEGG pathways for protein processing in the endoplasmic reticulum, Alzheimer’s disease, MAPK signaling pathway in PITA-depleted cells; protein processing in endoplasmic reticulum, p53 signaling pathway and osteoclast differentiation in PISA-depleted cells (Supplementary information, Figure S6I). These results shed light on understand why PITA and PISA execute such diverse functions controlling cell fate. To understand the general effect of depletion PITA or PISA in HCT116 p53^+/+^ cells on the p53 signaling pathway, we selected and investigated the DEGs (including GADD45B, ZMAT3, DDB2, CCNB1, MDM2 and RRM2B) associated with p53-signaling pathway. These DEGs have been reported to play fundamental roles in p53-mediated cell cycle arrest, DNA repair and apoptosis. Consistent with the RNA-seq data, the results showed that depletion of PITA enhanced the expression of GADD45B and DDB2, but not that of ZMAT3, MDM2, RRM2B, and CCNB1. Depletion of PISA enhanced the expression of GADD45B, DDB2 and CCNB1, but not that of ZMAT3, MDM2, and RRM2B in HCT116 p53^+/+^ cells (Fig. 6e). By contrast, shRNA knockdown of PITA or PISA had no significant effect on these DEGs in HCT116 p53^−/−^ cells (Supplementary information, Figure S6J), confirming the dependence of p53.

Given that PITA and PISA contain potential ATM phosphorylation sites, similar to the previously identified Apak, we next explored whether PITA and PISA regulated p53 activity in ATM-defective ATS4 fibroblast cells. We observed that PITA and PISA had much lower activity in ATS4 cells (Supplementary information, Figure S6K). To confirm the requirement for ATM in PITA or PISA function, we re-introduced ectopic ATM into ATS4 cells; the inhibitory effect of PITA or PISA on p53 activity was restored (Supplementary information, Figure S6K). Knocking down ATM in HCT116 p53^+/+^ cells significantly suppressed PITA or PISA activity (Fig. 6f and Supplementary information, Figure S6L).

Because p53 lies at a signaling node in response to diverse cellular stresses and ATM is crucial to p53 activation, we investigated the cooperation of ATM with PITA and PISA in response to glucose starvation, which is known to induce ATM autophosphorylation on Ser 1981. The inhibitory activity of PITA or PISA was markedly decreased by treatment in ATM-WT HCT116 p53^+/+^ cells, whereas ATM knockdown resulted in complete insensitivity of p53 activity to either PITA or glucose starvation at 24 h and to either PISA or glucose starvation at 36 h (Supplementary information, Figure S6M). In contrast, in ATM-mutated (MT) ATS4 cells, PITA or PISA activity did not change significantly in response to glucose starvation, and reintroduction of Flag-ATM restored their sensitivities (Supplementary information, Figure S6N). PITA dissociated from p53 after glucose starvation treatments at 24 h, and PISA dissociated from p53 after glucose starvation treatments at 36 h in HCT116 p53^+/+^ cells. Knocking down ATM in HCT116 p53^+/+^ cells prevented dissociation of PITA-p53 or PISA-p53, indicating that dissociation was dependent on ATM (Fig. 6g). In contrast, PITA or PISA bound tightly to p53 in stressed ATS4 cells (Supplementary information, Figure S6O). Analysis of PITA and PISA with Scansite software (http://scansite.mit.edu) at a high stringency revealed that Ser 18 of PITA and Ser 29, Ser 35 and Ser 58 of PISA were potential ATM phosphorylation sites. Mutation analysis showed that the PITA S18A and PISA S58A mutations resulted in constitutive binding to p53 and repression of p53 activity even in the stressed cells. Conversely, the PITA S18D and PISA S58D mutations, mimicking constitutive phosphorylation, disrupted PITA-p53 or PISA-p53 interactions (Supplementary information, Figure S6P and Q). These results suggest that PITA and PISA dissociation from p53 upon glucose starvation requires ATM-mediated phosphorylation. Additionally, PITA promoted activity of PFK1 and modulated the glycolytic rate dependent on the presence of ATM (Fig. 6h). Correspondingly, PISA decreased COX activity and modulated glycolytic activity dependent on expression of ATM (Fig. 6i).

### PITA and PISA protein levels were elevated in colorectal cancer

**Fig. 7:**
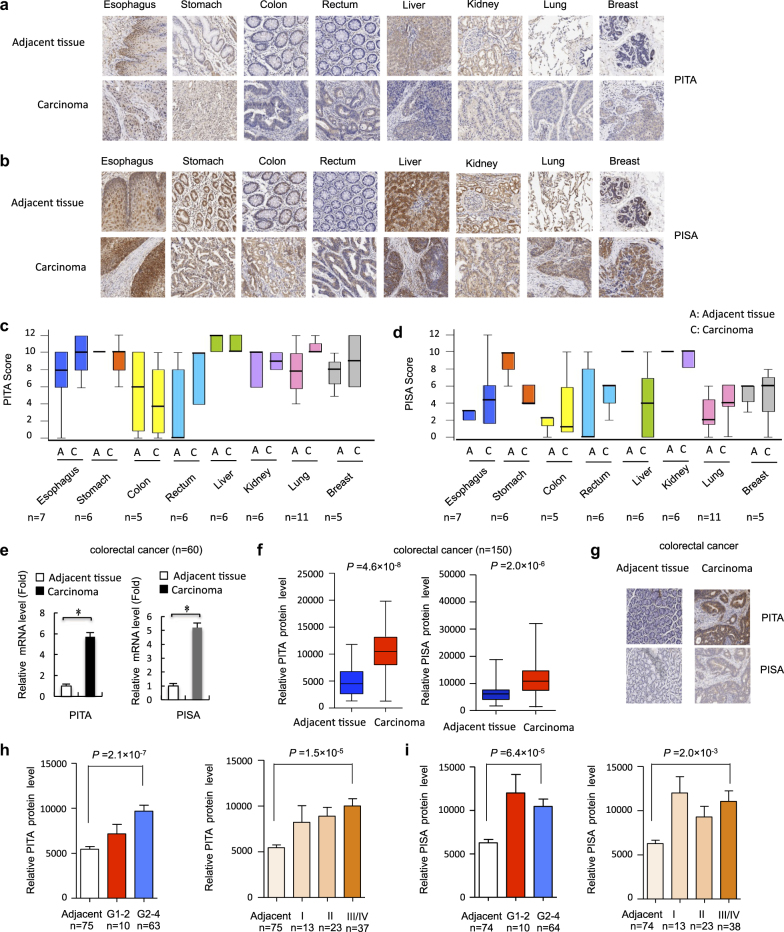
The expression of PITA and PISA is elevated in human tumor tissues. **a**, **b** Images of immunohistochemical staining for PITA and PISA in 8 types of paired cancers and normal tissues. Scale bar, 50 µm. **c**, **d** Box plot of PITA and PISA expression in 8 types of cancers and normal tissues. **e** Quantitative RT-PCR analysis of PITA and PISA mRNA level in rectal cancer tissue and matched adjacent normal control from 60 subjects. Error bars represent mean ± s.d. for three independent experiments. Error bars represent mean ± SD for three independent experiments. *P < 0.05: two-tailed unpaired *t*-test. **f** PITA and PISA protein levels in colorectal cancer tissue (*n* = 150). The positively stained nuclei (%) in grouped samples were analyzed by two-tailed unpaired *t*-test. **g** Representative images of immunohistochemical staining for PITA and PISA are shown. Scale bar, 50 µm. **h**, **i** The positively stained nuclei (%) in grouped samples were analyzed by two-tailed unpaired *t-*test. (histological grades I, II, III, and IV or tumor stages I–IV). The positively stained nuclei (%) in grouped samples were analyzed by two-tailed unpaired *t*-test

Many cancer cells shift their metabolism towards glycolysis – even under aerobic conditions – to rapidly produce energy and diverse metabolic intermediates for anabolic pathways. Alterations in metabolic pathways play a role in tumorigenesis, with mutations as well as changes in the expression of metabolic enzymes contributing to metabolic transformation.^21^ Because overexpression of PITA and PISA enhanced glycolysis rates, we hypothesized that PITA and PISA may play a role in human tumorigenesis. First, we screened the expression spectrum of PITA and PISA in the tumor and adjacent tissues from eight types of human cancers by IHC staining. In the adjacent normal tissues, PITA proteins were highly expressed in esophagus, stomach, colon, liver, kidney, lung and breast, but weakly detected in rectum (Fig. 7a). PISA proteins were highly expressed in stomach, liver, kidney, lung and breast, but weakly detected in esophagus, colon and rectum (Fig. 7b). Second, we screened tumor tissues and found that PITA and PISA proteins were significantly upregulated in rectal tumor tissues compared with matched adjacent normal tissues (Fig. 7c, d). Moreover, mRNA levels of PITA and PISA in colorectal tumor tissues were significantly higher than those in matched adjacent tissues (Fig. 7e). A higher frequency of positive PITA and PISA expression was observed in 150 colorectal cancer tissues (Fig. 7f, g) and the levels of their expression were positively correlated with the histological grades of the tumors (Fig. 7h, i).

It has been reported that p53 mutants have either low or no transactivation function compared with wild-type p53. The function of mutated p53 is dependent on mutant status and associated proteins. And in this case, the interaction between p53 and PITA/PISA might be the critical factor for p53 transcription activity and function. We first detected function of PITA and PISA in regulating p53 transcriptional activity in normal cells. We established stable normal colonic epithelial NCM460 cells transfectants ectopically expressing PITA and PISA, and found that overexpression PITA and PISA decreased the expression of TIGAR and SCO2, respectively (Supplementary information, Figure S7A). Overexpression of PITA or PISA also inhibited the transcription factor activity of p53 in NCM460 cells (Supplementary information, Figure S7B). Next, we explored whether tumor-derived p53 mutations had any effect on the interaction between PITA or PISA and p53. We selected several colon cell lines possessing p53 mutations to examine possible endogenous PITA-p53 or PISA-p53 interaction, including HCT116 p53^+/+^ (WT), HT29 (R273H) and SW480 (P309S) cell lines and normal colonic epithelial NCM460. Co-IP assays showed that endogenous P309S (SW480) and R273H (HT29) mutant p53 proteins had very-weak interaction with PITA or PISA, wild-type p53 in HCT116 cells had comparable binding affinity to PITA/PISA compared with p53 in NCM460 cells (Supplementary information, Figure S7C). Our previous study showed that certain mutants of p53 had either low (e.g., A266E and E285K) or almost undetectable transcriptional activity (e.g., R248Q, R273C, R273H and R280K) on the pG13L reporter compared with wild-type p53. Then, we detected whether PITA or PISA had any effect on the tumor-derived p53 mutants. Overexpression of PITA or PISA significantly inhibited the transcription factor activity of p53 in HCT116 (WT) cells. Similar results were obtained in NCM460 cells, but not in HT29 (R273H) and SW480 (P309S) cells (Supplementary information, Figure S7D). These data indicate that certain physiologically occurring mutations of p53 in human tumors weakened the interaction of p53 with PITA/PISA.

### PITA and PISA promote tumorigenesis in colorectal cancer

**Fig. 8:**
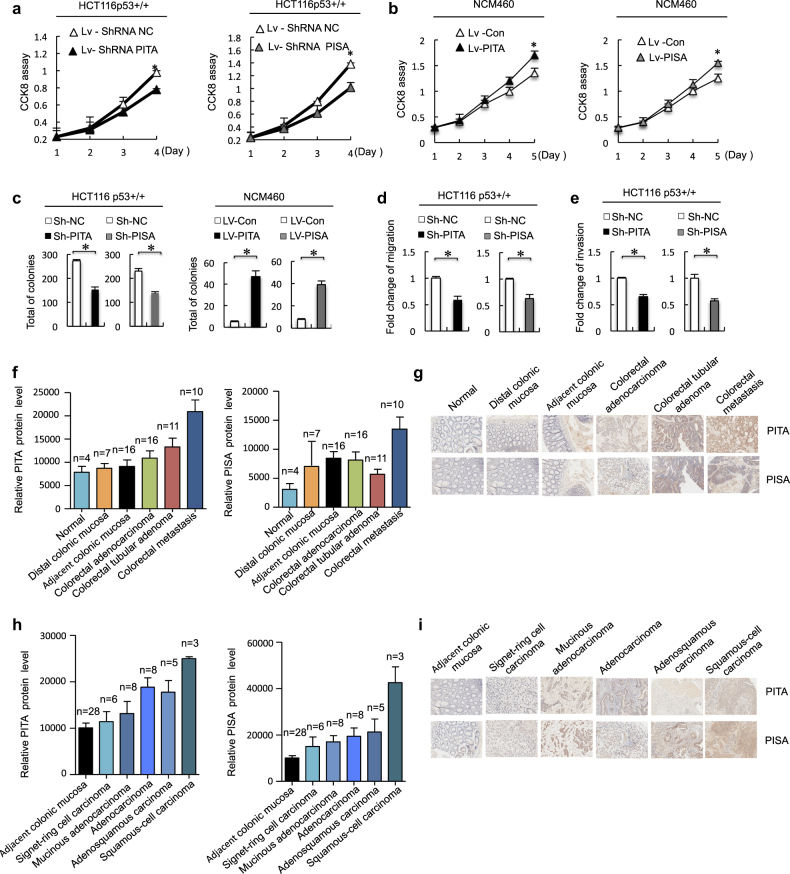
PITA and PISA promote tumorigenesis. **a** PITA or PISA shRNA or NC (negative control) shRNA lentivirus were used to infect HCT116 p53^+/+^ cells, and cell viability was measured at the indicated time points using CCK8 assays. **b** The human normal colonic epithelial NCM460 cells were infected with overexpression of PITA or PISA (or control) lentivirus, and the cell growth was measured by CCK8 assays. **c** Left, cells (2 × 10^4^) infected with lentivirus as in **a** were cultured in soft agar for 2–3 weeks. The colonies were counted and the relative number of colonies in experimental group was normalized to control group. Right, soft agar assays with PITA or PISA overexpression in NCM460cells were performed as described **b**. **d**, **e**Depletion of PITA or PISA reduced the migration **d** and invasion **e** of colon cancer HCT116 cells. The number of migrated cells was calculated. **f** PITA and PISA protein levels in progression of colorectal cancer tissue (*n* = 60). **g** Representative images of immunohistochemical staining for PITA and PISA are shown. Scale bar, 50 μm. **h** PITA and PISA protein levels in different type of colorectal cancer tissue (*n* = 80). **i** Representative images of immunohistochemical staining for PITA and PISA are shown. Scale bar, 50 µm. Error bars represent mean ± SD for three independent experiments. (**a**–**d**; mean and s.e.m., *n* = 3. *P < 0.05: two-tailed unpaired *t*-test)

**Fig. 9:**
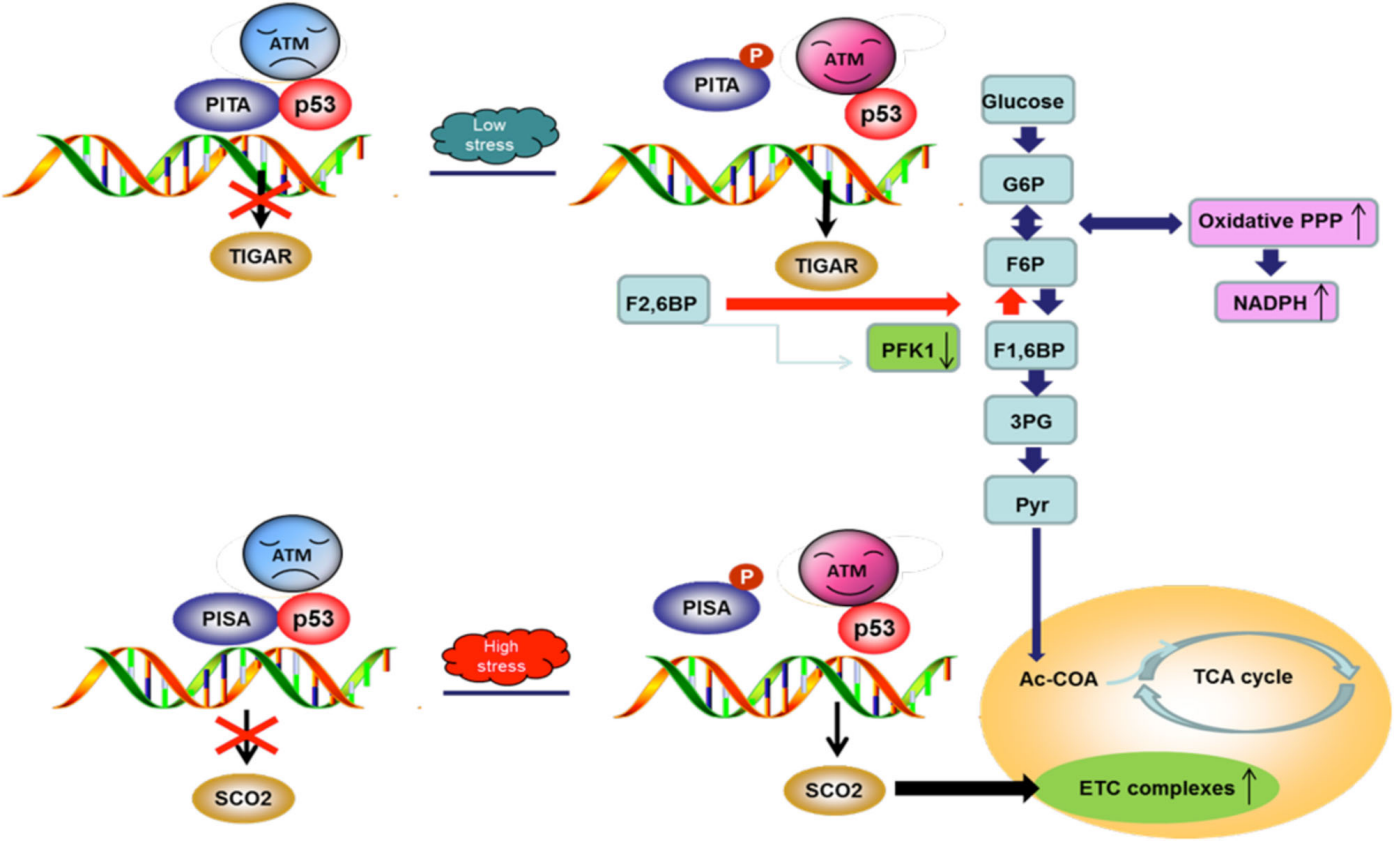
Proposed model for PITA and PISA function and regulation in unstressed and glucose starved cells. In unstressed cells, the KRAB-type zinc-finger protein PITA or PISA interacts with p53 and selectively inhibits p53-mediated transcription of metabolic gene *TIGAR* and *SCO2*, respectively. The expression of TIGAR and SCO2 is kept at relatively low levels. In response to glucose starvation, the ATM kinase is activated by autophosphorylation and promotes PITA and PISA phosphorylation, resulting in the dissociation of PITA or PISA from p53. Low stress stimulated the transcription of TIGAR and TIGAR protein in turn inhibits the PFK1 activity, decreases the lactate level, increases NADPH level and modulates the glycolytic rate. At the later stage upon glucose starvation or the cells are subjected with prolonged high stress, the upregulation of SCO2 occurred and the SCO2 protein promotes COX activity and modulates oxidative phosphorylation. PITA and PISA coordinate with ATM to tightly control the p53 downstream effects upon metabolic stress

Our results suggest a possible role of PITA and PISA in the development of human cancers. As support for this hypothesis, we set out to investigate cell growth, cell viability and tumorigenicity. PITA and PISA shRNA lentivirus-infected HCT116 p53^+/+^ cells showed decreased cell growth and increased sensitivity to apoptosis compared to the control cells. However, these effects were not observed in HCT116 p53^−/−^ cells (Fig. 8a and Supplementary information, Figure S8A and B). Conversely, PITA-overexpressing and PISA-overexpressing HCT116 p53^+/+^ cells exhibited induced cell viability, but these were not observed in HCT116 p53^−/−^ cells (Supplementary information, Figure S8C and D). To further assess the tumorigenic role of PITA and PISA, we sought to determine whether ectopic expression of PITA and PISA would transform colonic epithelial NCM460 cells. As shown, overexpression of PITA or PISA was able to enhance cell growth of NCM460 cells (Fig. 8b). The soft-agar colony-formation assays showed that ectopic expression of PITA or PISA in NCM460 cells resulted in the formation of transformed colonies (Fig. 8c). Correspondingly, depletion of PITA or PISA in HCT116 p53^+/+^ cells significantly inhibited the colony formation in the soft-agar medium (Fig. 8c). Interestingly, PITA or PISA knockdown had no significant effect on the formation of transformed colonies in HCT116 p53^−/−^ cells (Supplementary information, Figure S8E), indicating the PITA and PISA dependence on p53 in tumorigenesis. To further investigate the promotion of cell viability, we assayed PITA and PISA effects on colon cancer cell motility, with a cell migration and invasion assay. As shown in Fig. 8d, e, PITA and PISA konckdown inhibited cell migration and invasion in HCT116 p53^+/+^ cells, but not in HCT116 p53^−/−^cells (Supplementary information, Figure S8F and G). These results indicated that PITA or PISA upregulation might be candidate oncogenic factors in colon cancer development. To determine whether PITA and PISA play a possible role of development of colorectal cancer, we valuated different types of colorectal cancer tissue samples and colorectal cancer progression tissue samples. We screened 60 examples of different types of colorectal cancer tissue samples including normal, distal colonic mucosa, adjacent colonic mucosa, colorectal adenocarcinoma and colorectal metastasis, and found a higher expression of both PITA and PISA in colorectal metastasis (Fig. 8f, g). Furthermore, we screened 80 examples of colorectal cancer progression tissue samples and showed that PITA protein was upregulated in adenocarcinoma, adenosquamous carcinoma and squamous-cell carcinoma compared with matched adjacent normal tissues, and PISA protein was upregulated in squamous-cell carcinoma compared with matched adjacent normal tissues (Fig. 8h, i). The levels of PITA and PISA expression were positively correlated with the metastasis and histological grades of the tumors (Supplementary information, Figure S8H and I). In addition, expression of PITA and PISA were also upregulated during the colorectal tumor progression in mice induced by azoxymethane/dextran sodium sulfate (AOM-DSS) treatment (Supplementary information, Figure S8J). Taken together, these results suggest that PITA and PISA act as tumor-promoting proteins in colorectal cancer (Fig. 9).

## Discussion

Recent findings have indicated that p53 plays an important role in regulation of cellular metabolism, specifically glycolysis and mitochondrial respiration in cancerous cells. However, how p53 regulates metabolism and the relationship of this newly discovered role of p53 with its universal role as a tumor suppressor is not fully understood. We screened the whole KZNF family and identified two members, PITA (ZNF475) and PISA (ZNF568), as important negative regulators of p53-mediated glycolysis and mitochondrial respiration. This suggested a pathophysiological framework for PITA and PISA in energy homeostasis. Indeed, we found that PITA and PISA are highly expressed in rectal cancer tissues.

Known members of the KZNF family have a role in regulating embryonic development, cell differentiation, cell proliferation, apoptosis, neoplastic transformation and cell cycle regulation. Recently, a subset of KZNF family members have shown to be involved in p53 regulation via different molecular mechanisms. Apak (ZNF420) was identified as a specific inhibitor of p53-mediated apoptosis. In unstressed cells, Apak interacts with p53 in the nucleoplasm and inhibits p53 acetylation through recruitment of HDAC1, which selectively prevents p53-mediated transcriptional activation of proapoptotic target genes.^10^ Additionally, Apak binds directly to the proapoptotic *p53AIP1* gene, thus competitively hindering the binding of p53 to that p53RE and preventing p53AIP1 transactivation.^12^ ZNF307, another KZNF protein involved in p53 regulation, reduced p53 protein levels. This activates expression of MDM2 and EP300, resulting in p53 degradation.^22^ The major molecular mechanism of metabolism, which requires more time to achieve an increase (or decrease) in rate and efficiency of a metabolic pathway, is transcriptional control. In this study, we found that PITA selectively inhibits p53 binding to the *TIGAR* gene, and PISA selectively inhibits p53 binding to the *SCO2* gene. Based on our findings, we speculate that there may be two mechanisms by which PITA and PISA coordinate with p53 to modulate downstream outcomes. First, PITA or PISA binds to different sites adjacent to p53 responsive elements to selectively regulate specific response genes. Second, PITA or PISA binds to p53 and may influence the ability of p53 itself to preferentially bind to particular DNA target sequences. Our data showed that the SCO2 intron 1 contains a specific PISA-binding site. PISA directly competes with p53 binding to the *SCO2* gene and then inhibits SCO2 transcription. These findings explain why PISA selectively inhibits p53-mediated mitochondrial respiration. Our data also suggest that upon PITA binding to p53, p53 is preferentially inhibited from being recruited to introns or exons of TIGAR, although the interactions of PITA and TIGAR DNA sequences are still unclear.

Metabolic pathways can be modulated through allosteric mechanisms that control the activity of a regulatory enzyme. Key enzymes also regulate the rate of a metabolic pathway. As shown in Fig. 4b, PITA transgenic expression enhances PFK1 activity compared with WT mice. TIGAR may inhibit apoptosis via regulation of cellular NADPH levels.^4^ We observed that PITA decreased intracellular NADPH (Figs. 3a and 4d). This provides the first demonstration that PITA could significantly reduce the level of NADPH, the key reducing power in mammalian cells. This novel finding was consistent with the demonstration that specific downregulation of TIGAR by siRNA induced cancer cell death by altering the pentose phosphate pathway,^4^ which is the major pathway regulating production of cellular NADPH. Inhibition of glycolytic enzymes via other mechanisms led to re-routing the glucose flux toward anabolic processes.^23–25^ Our results suggest that PITA promotion of PFK1 might function upstream of glycolytic enzymes and is a major classical regulatory point for central carbon flow, exerting profound control over cancer cell metabolism.

We also found that PISA reduced SCO2 expression and decreased COX activity (Figs. 3f and 5b). Consistent with the inhibition of COX activity, PISA contributed to inhibition of basal oxygen consumption (Fig. 3f). Thus, PISA reduced mitochondrial capacity through inhibition of SCO2 and lowered COX activity in mitochondrial metabolism. Furthermore, PISA TG mice appeared to downregulate energy dissipation. These findings suggest that PISA decreased COX activity in association with decreased oxygen consumption, further suppressing downregulation of mitochondrial biogenesis. COX is believed to divert energy derived from the mitochondrial electron transport that normally generates ATP into heat production. PISA TG mouse models should be useful for future studies of SCO2 function, as well as in testing therapeutic approaches to treat human disorders.

Several lines of evidence support the concept that PITA and PISA play a crucial role in colorectal cancer development. First, IHC analysis of 150 rectal cancer specimens revealed that PITA and PISA are significantly upregulated in cancer tissues compared with adjacent tissues, although the mechanisms underlying this upregulation remain unknown. Second, PITA and PISA function as promoters of cell growth and tumor metastasis. Overexpression of PITA or PISA resulted in the formation of transformed colonies in colonic epithelial NCM460 cells. Depletion of PITA or PISA in HCT116 p53^+/+^ cells significantly inhibited the colony formation in the soft-agar medium. Finally, we found a higher expression of PITA and PISA both in different type of colorectal tumor tissues and colorectal metastasis. It suggested that PITA and PISA might play a possible role of development of colorectal cancer. Given the importance of metabolic reprogramming in tumor development, many oncogenes and tumor suppressor genes have been shown to help control these pathways.^26–28^ Cancer cells shift their metabolism towards glycolysis to support the biosynthetic demands necessary to sustain cell proliferation and growth. There are several possible reasons why PITA- or PISA-enhanced glucose uptake in glycolytic ATP generation or anabolic reactions constitute an advantage for tumor growth. PITA or PISA generates bicarbonic lactic acids and lactate, which is the principal end product of aerobic glycolysis. These compounds can alter their environment,^29^ favor tumor invasion^30^ and suppress anticancer immune effectors.^31^

p53 regulates energy metabolism, oxidative stress and amino acid metabolism by balancing glycolysis and mitochondrial respiration. KZNF-mediated transcriptional repression controls the expression of target genes in different tissues during development and differentiation, as well as in response to environmental changes, such as nutrition, fasting and hormone stimulation. We have shown that in response to glucose starvation, the dissociation of PITA from p53 at an early time point (24 h) allows p53-induced glycolysis to occur and causes dissociation of PISA from p53 at later times (36 and 48 h), promoting p53-induced mitochondrial respiration. Thus, PITA and PISA regulation of p53-mediated transcription of TIGAR and SCO2 is another tool in the armory of p53 to protect cells by repairing cells or effectively driving non-repairable cells toward cellular apoptosis (Fig. 9). This regulation can be achieved by posttranslational modifications or through p53-binding proteins capable of modulating p53 transcriptional activity. Elucidation of their highly specific tissue distribution and physiological function may contribute to knowledge of the spatial and temporal complexity of p53 regulation.

In conclusion, this study examined the effects of PITA and PISA to highlight the roles of KZNF proteins in glycolysis and mitochondrial metabolism. The analysis of PITA and PISA revealed metabolic-specific TIGAR and SCO2 regulation as a tipping point for metabolic dysfunction. KZNF proteins may be an important and understudied determinant of individual traits, including susceptibility to complex diseases. However, these proteins have not been assessed in genome-wide association studies due to their repetitive nature and poor annotation. Given the broad regulatory effects of PITA and PISA elucidated in metabolic pathways, the task of identifying functions of the hundreds of KZNF proteins extant in the human genome is likely to be important for promoting human health.

## Materials and methods

### Luciferase reporter assay

Luciferase reporter plasmids pG13-Luc (containing 13 tandem p53-binding elements) and pRL-CMV (internal control; from Promega) were co-transfected as indicated in figure legends. The TIGAR intron sequence and mutated sequences were each cloned into pGL3 luciferase plasmid.^4^ Luciferase reporter plasmids SCO2 reporter were obtained from Paul Laboratories.^5^ After transfection for 48 h, the cells were lysed in lysis buffer (Promega). Luciferase activity was measured with the Dual Luciferase Assay System (Promega) in accordance with the manufacturer’s protocol as previously described.^10^

### Chromatin immunoprecipitation (ChIP) assay

Cells were treated with 1% formaldehyde at 37 °C for 10 min. After being washed with ice cold PBS, the cells were suspended with 2 ml SDS lysis buffer (1% SDS, 10 mM EDTA and 50 mMTris-HCl, pH 8.1) on ice for 10 min. Lysates were sonicated and insoluble materials were removed by centrifugation. Supernatants were then precleared with 80 μl protein-A beads for 1 h at 4 °C. The precleared chromatin solutions were immunoprecipitated with normal mouse IgG, anti-p53 antibody, normal Rabbit IgG, anti-PITA and PISA antibody, respectively, at 4 °C overnight, followed by incubation with 60 μl protein-A beads for 6 h at 4 °C. The beads were washed with low-salt wash buffer (50 mM HEPES pH 7.5, 140 mM NaCl, 1% Triton X100, 0.1% deoxycholate sodium, protease inhibitors), and high-salt wash buffer (50 mM HEPES pH 7.5, 500 mM NaCl, 1% Triton X100, 0.1% deoxycholate sodium, protease inhibitors), then with LiCl wash buffer (10 mM Tris pH 8.0, 250 mMLiCl, 0.5% NP-40, 0.5% deoxycholate sodium, 1 mM EDTA) and twice with 1 ml of TE buffer. The samples were eluted with 2 ml elution buffer (1% SDS and 0.1 M NaHCO_3_). Crosslinks were reversed with heat at 65 °C for 6 h. Chromatin-associated proteins were digested with proteinase K at 45 °C for 1 h. Immunoprecipitated DNA was extracted with Phenol-chloroform followed by ethanol precipitation. Purified DNA was analyzed by real-time PCR, using SYBR Green mix (Bio-Rad) with MyIQ machine (Bio-Rad). Primers used for real-time PCR are given in Supplementary information, Table S8.

### Glucose consumption and lactate production

Cells were seeded in culture plates and cultured for 6 h, changed with fresh growth medium and incubated for an additional 15 h. Glucose levels in culture medium were measured using Glucose (GO) Assay Kit (Sigma). Lactate levels in culture medium were determined using Lactate Assay Kit (Sigma).

### PFK1 enzymatic assay

PFK1 activity was measured according to the GTX assay kit protocol. Absorbance was recorded at 340 nm at room temperature every 15 s for 10 min using a Beckman DU 640 spectrophotometer. One unit of PFK1 activity is defined as the amount of enzyme that catalyzes conversion of 1 μm fructose-6-phosphate to fructose-1, 6-bisphosphate per min.

### Cytochrome c oxidase (COX) activity assay

COX activity (reduced cytochrome c + DAB ⇒ oxidized cytochrome c + reduced DAB) was determined spectrophotometrically by increase in absorbance as reduced DAB accumulated (EmM 450 nm). The assay was generally performed as described^33^, and COX activity was determined as the azideinhibi table (1 mM NaN_3_) portion of the increase in absorbance at 450 nm for assay reagent containing 100 mM potassium phosphate, pH 7.0, 4 mM DAB, 5 μM reduced cytochrome c, 2 μg/ml catalase and 0.0033% saponin (GENMED assay kit).

### NADPH level assay

NADPH levels were determined as described previously.^32^ Cell lysates were prepared in buffer containing 0.1 M Tris-HCl, pH 8.0, 10 mM EDTA and 0.05% (v/v) Triton X-100, sonicated and centrifuged at 2,400×*g* at 4 °C. Total levels of NADPH plus reduced nicotinamide adenine dinucleotide (NADH) in lysates were assayed by spectrometry (Beckman DU 640 spectrophotometer) at a wavelength of 340 nm. The levels of NADPH in the lysates were determined on the basis of decrease in absorbance at 341 nm after NADH was converted to nicotinamide adenine dinucleotide (NAD) by glutathione reductase.

### Glucose tolerance test and hyperglycemic clamp

The glucose tolerance test (GTT) was carried out on 8–10-week-old male mice unless otherwise indicated. Prior to studies, mice were fasted overnight (GTT). In GTT studies, the mice were injected intraperitoneally (IP) with d-glucose solution (2 g/kg body weight). The tail blood glucose concentration was measured at 0, 15, 30, 60 and 120 min with a commercially available ONETOUCH Ultra blood glucose meter and test strips (Life Scan). A hyperglycemicclamp was performed, and calculated as described previously.^33^

### Generation of PITA and PISA transgenic mice

Microinjection was used to produce PITA and PISA transgenic mice, C57BL/6J female mice were induced to give superovulation by injecting hormone. Fertilized eggs were collected and microinjected with genomic DNA fragments containing PITA and PISA DNA into pronuclei. cDNA encoding Myc-tagged PITA or PISA was cloned from human cDNA. After microinjection, the fertilized eggs were implanted into the oviducts of pseudo pregnant female mice. The presence of the transgene in F0 and F1 mice was determined by PCR on genomic DNA with a forward primer in the pCMV-Myc promoter sequence and a reverse primer in the human PITA or PISA sequence. Results from PCR analysis were confirmed by Q-PCR analysis. C57BL/6J mice were obtained from Animal Care and Use Committee of Academy of Military Medical Sciences. Littermates of appropriate genotypes were used at 9–20 weeks of age. For this study, we used PITA, PISA transgenic mice and age matched wild-type (WT) controls.

### Analysis of PITA and PISA transgenic mice

Three-month-old PITA and PISA transgenic mice (C57BL/6) were maintained on a regular diet. PITA MEF cells were generated for measurement of glucose consumption and lactate levels. PITA transgenic tissues were excised for measurement of PFK1 activity, F2,6BP activity, PDK activity and NADPH levels. PITA transgenic mouse serum was collected for measurement of GSH/GSSG (ratio) levels. PISA transgenic mouse MEF cells were generated for measurement of glucose consumption, lactate levels. PISA tissues were excised for measurement of COX activity. PISA transgenic mouse serum was excised for measurement of glucose, adiponectin, cholesterol, NEFA and triglyceride levels and ATP (ratio) levels. PISA transgenic mouse muscle mitochondria were excised for measurement of mitochondrial volume and mitochondrial numbers. PITA and PISA were excised for measurement of walnut extract on rolling bar and indirect calorimetry. All animal experiments were carried out in accordance with the local Animal Care and Use Committee.

### Energy expenditure for mice

Energy expenditure was measured using the Columbus Instruments Oxymax System, which monitors O_2_ and CO_2_ gas fractions at both the inlet and output ports of up to 16 chambers through which a known flow of air is passing.

### MRI analysis for mice

MRI analysis of body fat content was carried out using an EchoMRITM whole body composition analyzer (Echo Medical Systems).

### Electrophoretic mobility shift assay (EMSA)

Double-stranded oligonucleotides used for EMSA were end-labeled with biotin. The labeled probes were incubated with the protein(s) for 30 min in binding buffer (10 mM Tris-HCl (pH 7.5), 5 mM KCl, 5 mM MgCl_2_, 10 mM ZnSO_4_, 50 mg/ml of poly [dI–dC], 5 mg/ml bovine serum albumin, 0.67 mM dithiothreitol, 0.67 mM phenylmethylsulphonyl fluoride, 2.5% glycerol) in the presence or absence of unlabeled probes. If an antibody was added to detect the supershift, the antibody and protein were pre-incubated for 20 min before the labeled probes were added. Protein/DNA-binding samples were loaded onto a native 6–10% polyacrylamide gel in TBE (Tris/borate/EDTA) buffer and then transferred to a Biodyne membrane. The membrane was blocked and incubated with HRP-conjugated streptavidin for 15 min and then washed for three times, treated with Super Signal (Pierce Biotechnology) detection reagents and exposed to Kodak Light films. The probe sequences of p53 targets are listed in the Supplementary information, Table S9.

### Immunoprecipitation and immunoblotting

Cells were harvested at 48 h post-transfection and lysed in HEPES lysis buffer (20 mM HEPES, pH 7.2, 50 mM NaCl, 0.5% Triton X-100, 1 mM NaF and 1 mM DTT) supplemented with protease inhibitor cocktail (Roche). Immunoprecipitations were performed using indicated primary antibody and protein A/G-agarose beads (Santa Cruz) at 4 °C. Lysates and immunoprecipitates were examined with indicated primary antibodies followed by detection with related secondary antibody and the Super Signal chemiluminescence kit (Pierce).

### GST pull-down assay

*Escherichia coli* BL21-expressed GST, GST-p53 bound to glutathione-Sepharose 4B beads (from GE) was incubated with bacterial-expressed His-PITA or His-PISA protein (purified by Ni-sepharose-agarose beads) for 2 h at 4 °C. Then the beads were washed four times with GST-binding buffer (150 mM NaCl, 10 mM Tris, 50 mM NaF, 2 mM EDTA, 0.5 mM Na_3_VO_4_ and 1% Nonidet P40) and proteins were eluted, followed by western blotting.^34^ The experimental condition was optimized and the concentration of NaCl in GST-binding buffer was 150 mM.

### RNA-sequencing and gene expression analysis

The RNA-seq library was prepared for sequencing using standard Illumina protocols. Sh-NC, Sh-PITA and Sh-PISA of total RNAs from HCT116 p53^+/+^ cells with or without glucose starvation (5 mM) and isolated using TRIzol reagent (Invitrogen) and treated with RNase-free DNase I (New England Biolabs, MA, USA), to remove any contaminating genomic DNA. Library construction and sequencing were performed on a BGISEQ-500. Clean-tags were mapped to the reference genome and genes available with a perfect match or one mismatch. For gene expression analysis, the matched reads were calculated and then normalized to RPKM using RESM software. The significance of the differential expression of genes was defined by the bioinformatics service according to the combination of the absolute value of log2-Ratio ≥1 and FDR ≤ 0.01. KOG functional classification, Gene Ontology (GO) and pathway annotation and enrichment analyses were based on the NCBI COG (https://www.ncbi.nlm.nih.gov/COG/), Gene Ontology Database (http://www.geneontology.org/) and KEGG pathway database (http://www.genome.jp/kegg/), respectively. The software Cluster and Java Treeview were used for hierarchical cluster analysis of gene expression patterns. The original sequence data have been submitted to the database of the NCBI Sequence Read Archive (http://trace.ncbi.nlm.nih.gov/Traces/sra) under the accession number SUB3220387).

### Cell migration and invasion

Boyden Chambers (24-well, 8 mm; Corning, Corning, NY, USA) were used to measure cancer cell migration and invasion. Cells (5 × 10^4^) in 100 ml serum-free RPMI 1640 medium were placed in the upper chamber, and 500 ml RPMI 1640 containing 10% fetal bovine serum was added to the lower compartment as a chemoattractant. After incubation for 36 h at 37 °C, cells on the top surface of the membrane were removed by wiping with a cotton swab. The migrated cells on the bottom surface of the membrane were fixed and stained with crystal violet. Cells were counted in four randomly selected fields. The invasion assay was performed in chambers coated with matrigel basement membrane matrix.

### Soft agar colony formation assay

NCM460 cells were infected with lentivirus as indicated. Six-well plates with 0.6% agar in Dulbecco’s modified Eagle’s medium as the bottom layer were used for the soft agar colony-formation assay. For each well, 2 × 10^4^ cells suspended in Dulbecco’s modified Eagle’s medium with 0.3% agarose were plated as the top layer and incubated at 37 °C, for 2–3 weeks. Colonies were counted under microscope, and the relative number of colonies in experimental group was normalized to the control group.

### Immunohistochemical (IHC) staining

We performed IHC staining on the same paraffin-embedded tissue blocks that were used for clinical diagnosis. Immunohistochemistry was performed with the avidin-biotin complex method (Vector Laboratories), including heat-induced antigen-retrieval procedures. Incubation with polyclonal antibodies against PITA (1:500 dilution; Abcam) and PISA (1:500; Abcam) was performed at 4 °C for 18 h. Quality assessment was performed on each batch of slides including a negative control in which the primary antibody was replaced by 10% normal goat serum to preclude non-specific signals. Staining was assessed by pathologists who were blinded to sample origins and patient outcomes. The widely accepted German semi-quantitative scoring system was used to score staining intensity and extent in different areas. Each specimen was assigned a score according to the intensity of the nucleic, cytoplasmic and membrane staining (no staining = 0; weak staining = 1, moderate staining = 2, strong staining = 3) and the extent of stained cells (0–5% = 0, 5–25% = 1, 26–50% = 2, 51–75% = 3, 76–100% = 4). Image Pro plus 6.0 (Media Cybernetics Inc., Silver Spring, MD) was used to analyze optical densitometry.

### Engineered CRISPR-Cas9 MEF

The PITA and PISA sgRNA purchased from Obio Technology (Shanghai) Corp. Ltd. The sgRNA sequence of PITA 1: 5′-ACCGCATGTATAGGATCCTCTGAG-3′; 2: 5′-ACCGAATTGCCTCGCCTGCTCGTC-3′; and 3: 5′-ACCGAGATCGCCTCTTTCCTCAAT-3′. The sgRNA sequence PISA 1: 5′-ACCGTTTGAGCCACATGATGGAA-3′; 2: 5′-ACCGTATCCTCTTCTTCCTCGGAA-3′; and 3: 5′-ACCGCTCCCACTCCTCCTGGGTA-3′. CRISPR-Cas9 was performed as previously described.^34, 35^

### Tissue microarray

After screening hematoxylin and eosin-stained slides for normal tissues or optimal tumor content, we constructed tissue microarray slides (Shanghai Biochip Company, Ltd., Shanghai, China). Two cores of tissue were collected from non-necrotic areas of tumor foci and from peritumoral tissue adjacent to the tumor. To assess the possibility that positive expression reflected a direct effect of the tumors, we took peritumoral tissue within 10 mm. Punch cores with a longest dimension of 1.0 mm were used. TMA arrays contain a multiple-tumor tissue microarray and a rectal tumor tissue microarray. Sections (4 μm) of the resulting TMA blocks were prepared using standard techniques.

### Statistical analysis

All results are expressed as mean ± SD derived from three independent experiments, unless stated otherwise. A Student's *t*-test or a Mann-Whitney test (for two group comparisons) was used in statistical analyses. All statistical analyses were performed with GraphPad Prism 5 and SPSS 19.0 software. The *χ*^2^ test was used to compare qualitative variables. Protein expression in rectal cancer tumors and matched adjacent tissue was compared with the Wilcoxon test. Score comparisons between groups were assessed using the Kruskal-Wallis test. All statistical tests were two-sided, and *P* < 0.05 were considered to be statistically significant.

## Electronic supplementary material

Supplementary Information

Supplementary information, Figures S1-S8

Supplementary information, Figure S9

Supplementary information, Table S1

Supplementary information, Table S2

Supplementary information, Table S3

Supplementary information, Table S4

Supplementary information, Table S5

Supplementary information, Table S6
