# Supplementary material for: KRAB-type zinc-finger proteins PITA and PISA specifically regulate p53-dependent glycolysis and mitochondrial respiration
Source: Cell Res. 2018 Feb 21;28(5):572–92. doi: 10.1038/s41422-018-0008-8 (PMC5951852; doi:10.1038/s41422-018-0008-8)
Supplement: Supplementary file 1 — Supplementary Information [file 41422_2018_8_MOESM1_ESM.doc]

**Supplementary Information**

“KRAB-type zinc-finger proteins PITA and PISA specifically regulate p53-dependent glycolysis and mitochondrial respiration" by Shan Wang *et al*.

**Supplementary Methods**

**Plasmid constructs, antibodies and reagents**

ZNF475, the human gene encoding PITA, and ZNF568, the human gene encoding PISA, were amplified from a human fetal liver complementary DNA library (Clontech, BD Biosciences). Plasmids of human PITA, PISA and p53 including deletion truncations and point mutants were constructed by PCR and recombinant PCR, followed by subcloning into various vectors. A series of p53-truncated plasmids were gifts from Dr. Shengcai Lin (Xiamen University, Fujian Province, China) and have been described previously ^1^. Antibodies used were anti-Flag, anti-Myc-HRP and anti-Flag-HRP (Sigma), anti-Myc, anti-GST (MBL), anti-His (CST), anti-p53 (Proteintech), anti-PITA, anti-PISA，anti-SCO2, anti-TIGAR, anti-sestrin1, anti-GLS2, anti-GAMT and anti-G6DP (abcam), anti-PGM1 (Cell Signaling), anti-CPT1 (Proteintech), anti-14-3-3σ (Cell Signaling), anti- p53AIP1 (Bethyl Laboratories, Inc).

**Cell culture, cell transfection and cell treatment**

HCT116 p53^+/+^ and HCT116 p53^−/−^ colon cancer cells were a gift from Dr. Qimin Zhan (Chinese Academy of Medical Sciences and Peking Union Medical College, Beijing, China). The SW480, HT29 colon cancer cell lines and human normal colonic epithelial NCM460 cells were purchased from ATCC (Rockefeller, MD, USA) and authenticated by STR profiling and tested for mycoplasma contamination by GENEWIZ. All cell lines cultured in RPMI 1640 or Dulbecco’s modified Eagle’s medium media (Hyclone, Logan, UT, USA) containing 10% fetal bovine serum. Cells were transfected with various plasmids using or Lipofectamine 2000 (Invitrogen) reagent according to the manufacturer's protocol. Glucose-free medium was prepared with DMEM base (GIBCO, #11966) and supplemented with glucose (Sigma, G7528) as indicated.

**Lentivirus infection**

The PITA shRNA sequence (5´-ACTTCTCTACCTGAAGCAA-3´), the PISA shRNA sequence (5´-ACTTCTCTACCTGAAGCAA-3´), the control sequence (5´-agaggtcaactcttagatt-3´) were purchased from Genepharma. The lentiviral expression vector for PITA or PISA was constructed by inserting full-length complementary DNA into pWPT vector. Mammalian cells were transfected with Lipofectamine 2000 (Invitrogen) in accordance with the manufacturer’s instructions. The viruses were used to infect HCT116 p53^+/+^, HCT116 p53^−/−^ and NCM460 cells in the presence of polybrene. Forty-eight hours later, HCT116 p53^+/+^, HCT116 p53^−/−^ and NCM460 cells were cultured in medium containing puromycin for the selection of stable clones. The clones stably knocking down PITA or PISA were identified and verified by western blotting using the indicated primary antibodies.

**Fluorescence microscopy**

For detection of subcellular localization by immunofluorescence, after fixed with 4% paraformaldehyde and permeabilized in 0.2% Triton X-100 (PBS), cells were incubated with the indicated PITA or PISA and p53 antibodies (dilution 1:50; Abcam) for 8 h at 4 °C, followed by incubation with TRITC-conjugated or FITC-conjugated secondary antibody (dilution 1:200; Cwbio) for 1 h at 25 °C. F-actin was detected withActin-stain™ 555 phalloidin (Cytoskeleton). The nuclei were stained with DAPI (Sigma), and images werevisualized with a Zeiss LSM 510 Meta inverted confocal microscope.

**Cell fractionation**

Cytoplasmic and nuclear fractions were separated by using NE-PER Nuclear and Cytoplasmic Extraction Reagents Kit (Thermo).

**Apoptosis assays**

Cells stably knockdown PITA or PISA were treated with cisplatin (10 μM, 24 h, Sigma) or DMSO. The apoptotic cells were then washed with PBS and stained with fluorescein isothiocyanate-Annexin V and propidium iodide according to the manufacture's protocol (Beijing Biosea biotechnology Annexin V Kit). Apoptotic cells (Annexin V positive, propidium iodide negative) were then determined by flow cytometry.

**ChIP-seq**

After ChIP-seq library was constructed, HiSeq2000sequencer (Illumina) was used to generate 101-base sequences. Sequencing reads were aligned to reference sequence (GRCm38/mm10) using MACS software [1]. Results generatedby MACS were loaded into IGV for visualization [2]. MultipleEm for Motif Elicitation (MEME) was used to search for PITA and PISA motifs [3]. PeakAnnotator was used to annotate information of each peak generated by MACS [4]. Average ChIP enrichment signals around TSS were displayed using Cis-regulatory Element Annotation System (CEAS).

**Real-time quantitative PCR**

Total RNA was isolated by Trizol kit (TianGen). RNA was treated with DNase. Complementary DNA was synthesized using the cDNA synthesis kit (ToYoBo) according to the manufacturer’s instructions. Fluorescence real-time RT-PCR was performed with the double-stranded DNA dye SYBR Green PCR Core Reagents (ToYoBo) using the ABI PRISM 7300 system (Perkin-Elmer, Torrance, CA). PCR was done in triplicate and standard deviations representing experimental errors were calculated. All data were analyzed by ABI PRISM SDS 2.0 software (Perkin-Elmer). Detailed information of the pairs of PCR primers used to amplify the target genes is given in the Supplementary Table 7.

**Redox-state analysis**

Cell lysates were prepared using a lysis buffer freshly supplemented with protease inhibitor cocktail for mammalian tissues (Biyuntian). Immediately following centrifugation to clear the lysates, aliquots of the supernatant were assayed for GSH in 25 mM KH_2_PO_4_ (pH 7.2) containing 20 μM ThioGlo-1, and for total glutathione after adding 100 μM NADPH and 3.5 U/ml glutathione reductase. Fluorescence intensities were monitored using an excitation wavelength of 405 nm and emission wavelength of 485 nm, and were converted to GSH concentrations by comparison with standards.

**Measurement of ROS**

HCT116 p53^+/+^ cells were stably transfected with PITA, PISA or the empty vector pcDNA3.1. A total of 10^5^ cells were seeded in 6 cm dishes, the cells were collected and lysed for ROS assay using Reactive Oxygen Species ELISA kit from GEMEND. ROS level were performed as previously described [2].

**Assays with MEFs**

Mouse embryonic fibroblasts (MEFs) were isolated from E13.5 embryos and cultured in DMEM medium supplemented with 10% FBS, penicillin/streptomycin and 2 mM L-glutamine using standard techniques. For serial 3T3 cultivation, 10^6^ cells were passage into 10-cm dishes every 3 days. Growth curves were generated by seeding 0.25╳10^5^ cells per well in 12-well plates, in triplicate. Plates were stained with crystal violet at each indicated time point. The dye was extracted with 10% acetic acid followed by plate reading at 590 nm. The values were normalized to the absorbance at day 0. For galactose experiments, cells were cultured in glucose-free DMEM with 4.5 g/L galactose (Sigma) in addition to the above additives. For colony-formation assays, 10^4^ cells were plated in triplicate in 10-cm dishes. Two weeks later, plates were stained with Giemsa and the number of visible colonies was scored.

**Oxygen consumption assay**

A total of 1╳10^6^ viable cells were plated in each well of an Oxygen Biosensor Plate (BD Biosciences) in media in the presence or absence of 2 mM FCCP (Sigma). Fluorescence was measured with an Envision Multilabel Plate Reader, with an excitation wavelength of 485 nm and emission wavelength of 633 nm every 2 min for at least 90 min. Fluorescence increases as oxygen is depleted from the media. The OCR (Drfu/min) was calculated as the rate of increase in fluorescence per minute from the linear part of the curve. Oxygen consumption recordings allowed the calculation of the respiratory control ratio (RCR) corresponding to the ratio between the state 3 (ADP-stimulated) respiratory rates and the state 4 (resting) respiration rate.

**ATP assay**

Cells were lysed in ATP releasing reagent (Sigma). ATP concentrations were determined by luciferase assay with the CLS II kit. Serum ATP was measured using the ATP Assay Kit (abcam), reader with an excitation wavelength of 485 nm.

**Measurement of pyruvate kinase activity**

Pyruvate kinase activity was measured by a continuous assay coupled to lactate dehydrogenase (LDH). The change in absorbance at 340nm owing to oxidation of NADH was measured using a Victor3 1420 Multilabel Counter spectrophotometer (PerkinElmer). Kinetic assays for activity determinations (triplicate samples) contained cell lysate (1–2 mg), Tris pH 7.5 (50 mM), KCl (100 mM), MgCl_2_ (5 mM), ADP (0.6 mM), PEP (0.5 mM), NADH (180 mM), FBP (10 mM) and LDH (8 units).

**Tissues extraction and mitochondrial isolation**

Fresh tissue was weighted for mitochondrial isolation after its muscle removed, rinsed and dried. Mitochondria were prepared with differential centrifugation as described in GENMED. For mitochondrial isolation, fresh tissues were rinsed with PBS and put into ice-cold isolation buffer (75 mM sucrose, 225 mM sorbitol, 1 mM EGTA, 0.1% fattyacid-free bovine serum albumin (BSA), and 10 mMTris–HCl, pH 7.4). Tissues were sheared carefully to mince, rinsed to get rid of residual, and then homogenized in 1 ml isolation buffer per 100 mg tissue. Homogenate was centrifuged at 1000 g for 5 min at 4 °C using a Beckman centrifuge (Avanti J-26XP); supernatant was decanted and saved. Pellet was washed with isolation buffer. Supernatant were combined and centrifuged at 9,000 g for 10 min at 4°C. The mitochondrial pellet was washed and centrifuged twice at 15,000 g for 2 min at 4°C with isolation buffer. Mitochondrial protein content was assayed using BSA as a standard according to Bradford.

**Mitochondrial enzyme activity assays**

Mitochondrial SDH activity was assessed spectrophotometrically with IBL assay Kit by decrease in absorbance related to reduction of DCIP (EmM 600 nm) generally as GENMED assay Kit. IDH activity was monitored spectrophotometrically with Abnova assay Kit by the rate of reduced NADH (EmM 340 nm). KDH activity was assayed by fluorometric monitoring with IBL assay Kit (excitation 360/40 nm, emission 460/40 nm) of the increase in fluorescence as NADH accumulated. Mitochondrial complex III activity was assessed in a spectrophotometric assay with GENMED assay Kit

**Analysis of mitochondrial volume and number**

Mitochondrial volume and number were assessed in a spectrophotometric assay with GENMED and AAT Bioquestassay Kit. To obtain a volumetric measurement z axis were acquired in 11 planes with 0.6 µm for step. Collected images were analyzes by the ImageJ software. IMorphometric measurements were obtained on the maximum intensity projection analyzed morphometric plugins. For each object revealed we obtained the “roundness” and “aspect ratio” parameters, useful to quantify circularity or elongation of an object respectively. We created a ratio of these parameters in order to obtain a "fragmentation index" that could be equally sensitive for the circular or elongated shape of a mitochondrion.

**Fatty acid oxidation**

Cells were incubated overnight in culture medium containing 100 M palmitate (C16:0) and 1 mMcarnitine. In the final 2 hours of incubation, cells were pulsed with 1.7 Ci [9,10 (n)-^3^H] palmitic acid (GE Healthcare), and the medium was collected to analyze the released 3H2O, formed during cellular oxidation of [^3^H] palmitate^3^. In brief, medium was TCA precipitated, supernatants were neutralized with NaOH and loaded onto ion exchange columns packed with DOWEX 1X2-400 resin (Sigma). The radioactive product was eluted with water and quantitated by liquid scintillation counting. Oxidation of [^3^H] palmitate was normalized to protein content using Bio-Rad DC protein assay.

**Lipid synthesis assay**

For measurement of glucose-dependent lipid synthesis^4^, 10^5^ cells per well were seeded in 12-well plates at t=-24 hours. At t=-1 hour the medium was changed to fresh growth medium. At t=0 the medium was replaced with fresh growth medium containing 4 µCi/ml [6-14C]glucose (final concentration=72 µM). Cells were then incubated for 2 hours at 37^o^C, 5% CO_2_. The labelling medium was then removed, cells were washed once with PBS at room temperature and lipids were extracted twice with 500 µl hexane:isopropanol (3:2 v/v), extracts were pooled and dried under nitrogen. Dried lipids were resuspended in 100µl chloroform and radioactivity was measured in a scintillation counter.

**Exercise training protocols**

Locomotor activity was recorded for 1 h in open field test. Total distance, central distance, central distance ratio, periphery distance, periphery distance ratio, central time and periphery time were used to evaluate the behavior characteristics of locomotor activity. 30 male C57BL/6 mice were assigned to a exercise training. Mice were housed 4–6 per cage at 21±1°C in standard cages with free access to food and tap-water. Rolling bar strength training was performed by the following experimental set up. A 8×5 cm hole was cut in an aluminium (AE) plate and covered with a metal wire mesh. The wire had a maximum diameter of 1 mm to provide mice a strong grip. The mice gripped with their front and their back paws on horizontal wires of the metal mesh and the plate was placed in a vertical position. Meanwhile the orientation of the mice was holding head up. The plain surface of the AE plate prevented a further movement away. Strength training was performed for 5 times for 3 minutes and 3 series. Running speed was 0.27±0.05 m/s corresponding to about 80% of VO_2_max. Break between each series was 1 minute. In order to determine isometric strength we used the same experimental set up like in the training protocol (maximum holding time  =  MHT). Briefly, the mice gripped with their paws on wires of the metal mesh and the plate was placed in a vertical position. Time was measured until mice released both back paws from the wire.

**SDS-PAGE and mass spectrometry**

Immunocomplexes were eluted in 13Laemmli buffer and resolved on pre-cast 4%–20% NovexTris-Glycine or 4%–12% NuPage gels (Invitrogen, CA, USA) to half-length. Gels were minimally stained with Coomassie brilliant blue and cut into 6 molecular weight ranges (heavy chain IgG band,) digested with trypsin. Immunocomplexes were identified on a Thermo Fisher LTQ (majority) or Velos-Orbitrap mass spectrometers. Spectral data were then searched against human protein RefSeq database in Bio Works or Proteome Discoverer Suites with either SeQuest (for LTQ data) or Mascot (Orbitrap data) software. Multi-consensus result of protein GI identifiers were compiled with strict filters and often manually inspected.

**Data processing**

IP/MS results were transferred into an in-house built FileMaker-based relational database where protein identification numbers (protein GIs) were converted to the Gene ID identifiers according to NCBI ‘‘gene-accession’’ table.

**Colitis-associated cancer induction**

(1) Set aside cages of sex and age-matched 6-8 week old mice to be used for experimental and control groups. Mice can be individually labeled with tail markings or ear clips. (2) On day 0, record baseline weights and inject each mouse intraperitoneally (IP) with 10 mg/kg of AOM working solution (1 mg/ml in isotonic saline, diluted from 10 mg/ml stock solution in dH_2_O kept at -20℃). Based on experience, this dose can be adjusted between 7-14 mg/kg and/or repeated early in the experiment. (3) Make a 2.5% (2.5 g/100 ml) DSS solution in distilled water and pass through a 0.22 μm cellulose acetate filter by vacuum. This dose can be adjusted between 1-3.5% depending on mouse strain and environment. Once prepared, DSS solution can be kept refrigerated for up to 1 week. (4) On day 7, supply DSS solution to mice as their drinking water. Approximately 250 ml/cage will be needed every time new DSS is provided for a maximum of 5 mice/cage; however, these are only estimations and will vary depending on the type of water bottles used in your animal facility. (5) To provide a continuous supply of DSS for seven days, DSS solution should be replaced in clean bottles three times (every 2-3 days) during this period. Some investigators measure the amount of DSS consumed prior to replacing with new solution as a measure of exposure. (6) On day 14, switch cages back to standard drinking water for two weeks. (7) Repeat steps (4) - (6) on days 28 and 49 to provide a second and third cycle of DSS. A DSS "cycle" consists of one week of DSS in thedrinking water followed by 2 weeks of regular (autoclaved) water. (8) On the day 60, mice were killed. Macroscopic tumors were counted. The clinical course of disease was followed daily by measurement of body weight and monitoring for signs of rectal bleeding or diarrhea. Mice colorectal tissues were a gift from Dr. Weiguo Hu (Fudan University Shanghai Cancer Center and Institutes of Biomedical Sciences, Collaborative Innovation Center of Cancer Medicine, Shanghai Medical College, Fudan University, Shanghai, China).

**Supplementary Table Legends**

**Supplementary Table 1** The distribution of all human PITA peaks across the genome

**Supplementary Table 2** Human PITA GO enrichment results

**Supplementary** Table 3 MRI analysis for WT mice and PITA or PISA TG mice

**Supplementary Table 4** RNA-seq analysis: DEGs of PITA-depleted cells and control cells under the condition of glucose starvation

**Supplementary Table 5** RNA-seq analysis: DEGs of PISA-depleted cells and control cells under the condition of glucose starvation

**Supplementary Table 6** List of potential p53 interacting proteins under the condition of glucose starvation

**Supplementary Table 7 Sequence of primers used in the real-time quantitative PCR**

| No | Name | F/R | Sequence |
| --- | --- | --- | --- |
| 1 | TIGAR | F | 5’- CGGAATTCAGAACAGTTTTCCCAAG-3’ |
| 2 | TIGAR | R | 5’- CGGAATTCAACCTTAGCGAGTTTC-3’ |
| 3 | SCO2 | F | 5’- GCAGCCTGTCTTCATCACTGTGGACC- 3’ |
| 4 | SCO2 | R | 5’- CCGCACACTGTCTGAGATCTGCTC- 3’ |
| 5 | Coxvb | F | 5’- CTATGCCAGGAACCCATCTC - 3’ |
| 6 | Coxvb | R | 5’- GAAGGCGACCATCAAACAGA - 3’ |
| 7 | Cycs | F | 5’-CCCTGATGGAGTATTTGGAG - 3’ |
| 8 | Cycs | R | 5’- GCTATTAGGTCTGCCCTTTCT - 3’ |
| 9 | Atp5g1, | F | 5’- CTGGATCAGGAGCTGGCATTG--3’ |
| 10 | Atp5g1, | R | 5’- AGGCGACCATCAAACAGAAG -3’ |
| 11 | Ucp2 | F | 5’- GCTGGTGGTGGTCGGAGATA-3’ |
| 12 | Ucp2 | R | 5’- ATTACGGGCAACATTGGGAG -3’ |
| 13 | mtTFA | F | 5’- CCCTCGTCTATCAGTCTTGT-3’ |
| 14 | mtTFA | R | 5’- ATTACGGGCAACATTGGGAG -3’ |
| 15 | Idh3a | F | 5’- GCTGCCAAAGCACCTATTCA-3’ |
| 16 | Idh3a | R | 5’- AGCCCATCTTGTTCTTATCC -3’ |
| 17 | Mfn-2 | F | 5’- F: CCTGTACGTCTATGAGCGACTG-3’ |
| 18 | Mfn-2 | R | 5’- AATTCCTGCTGGACTTGGTG -3’ |
| 19 | Drp-1 | F | 5’- TAAATGTCTTAGCTGCTCGGTAT |
| 20 | Drp-1 | R | 5’- TGCGGTTCCTTCAATCGTGT -3’ |
| 21 | NRF1 | F | 5’- AGCAAGTCCAGCAGGTCCAT- 3’ |
| 22 | NRF1 | R | 5’- GTCCGAGTCATCATAAGAAGTGTC - 3’ |
| 23 | NRF2b | F | 5’- TGGGAACTTCTCCACTTCATCT- 3’ |
| 24 | NRF2b | R | 5’- TCCACTTTGGTCCTGGCATCTC - 3’ |
| 25 | CD36 | F | 5’- ACACCCGAATCCTCACTGGAA-3’ |
| 26 | CD36 | R | 5’- GGACCCGTTGGCAAACAAAG-3’ |
| 27 | Ucp3 | F | 5’- AGGGAAGAAGGAGTCAGGG-3’ |
| 28 | Ucp3 | R | 5’- GGGAGCGTTCATGTATCG -3’ |
| 29 | PDK4 | F | 5’- CGGATGCTGATGAACCAACAC |
| 30 | PDK4 | R | 5’- GGACCACTGCTGCCACAT-3’ |
| 31 | PGC1α | F | 5’-GGACAGTCTCCCCGTGAT-3’ |
| 32 | PGC1α | R | 5’-TCCATCTGTCAGTGCATC-3’ |
| 33 | TFAM | F | 5’- GCTTCCAGGAGGCAAAGGAT-3’ |
| 34 | TFAM | R | 5’- CCAAGCCTCATTTACAAGC -3’ |
| 35 | Glut4 | F | 5’- TTCTGGATGATGTAGAGGTAGCGG- 3’ |
| 36 | Glut4 | R | 5’- GAAGGCGACCATCAAACAGA - 3’ |
| 37 | HK1 | F | 5’-GTGGTGCTTTTGACCCGTT - 3’ |
| 38 | HK1 | R | 5’- TTCGATGAAGGTGATTTCGCA |
| 39 | LDH-A | F | 5’- CTGGATCAGGAGCTGGCATTG--3’ |
| 40 | LDH-A | R | 5’- AGGCGACCATCAAACAGAAG -3’ |
| 41 | GOT1 | F | 5’- GCTGGTGGTGGTCGGAGATA-3’ |
| 42 | GOT1 | R | 5’- ATTACGGGCAACATTGGGAG -3’ |
| 43 | ChREBP | F | 5’- AAAGGCCTCAAGTTGCTATG-3’ |
| 44 | ChREBP | R | 5’- AGACAACAGCCTCAGGTTTC-3’ |
| 45 | aP2 | F | 5’- GCAAGTTTATCCTGGGTGG-3’ |
| 46 | aP2 | R | 5’- TGAAATTGATGTCTGCCTCC-3’ |
| 47 | GCNN5 | F | 5’- F: CCTGTACGTCTATGAGCGACTG-3’ |
| 48 | GCNN5 | R | 5’- AATTCCTGCTGGACTTGGTG-3’ |

**Supplementary T**able 8 Sequence of primers used in the ChIP-qPCR

| 1 | TIGAR | F | 5’-CGGCAGGTCTTAGATAGCTT-3’ |
| --- | --- | --- | --- |
| 2 | TIGAR | R | 5’-GGCAGCCGGCATCAAAAACA-3’ |
| 3 | SCO2 | F | 5’-GCCTGTCTTCATCACTGT-3’ |
| 4 | SCO2 | R | 5’-GCACACTGTCTGAGATCT-3’ |
| 5 | Puma | F | 5’-GCGAGACTGTGGCCTTGTGT-3’ |
| 6 | Puma | R | 5’-CGTTCCAGGGTCCACAAAGT-3’ |
| 7 | p21 | F | 5’-GGCTGGTGGCTATTTTGTCC-3’ |
| 8 | p21 | R | 5’-TCCCCTTCCTCCCTGAAAAC-3’ |

**Supplementary Table 9 The EMSA probe sequences of p53 targets**

| 1 | TIGAR | BS1 | 5’- CCCGGGCCTGTGGACTAGTCCACAAAGCAAGTCTCTGTAGAGCTC -3’ |
| --- | --- | --- | --- |
| 2 | TIGAR | BS2 | 5’-CTGTGGACTAGTCCACAAAGCAAGTCTCTGTAGAGCTCAGGAA-3’ |
| 3 | TIGAR | BS3 | 5’- CCCGGGGTGCCAGACATGTCCACAGACTTGTCTGGGTAGAGCTC -3’ |
| 4 | TIGAR | BS4 | 5’- AGCAGCTCCCCGGGGTGCCAGACATGTCCACAGACTTGTCT -3’ |
| 5 | SCO2 | BS | 5’-GTGCCAGGCAAGGCATGCACTTGGAAAGC -3’ |
| 6 | Puma | BS | 5’- CGCGCCTGCAAGTCCTGACTTGTCCGCGGC -3’ |
| 7 | p21 | BS | 5’-GTCAGGAACATGTCCCAACATGTTGAGCTC-3’ |

**Supplementary Figure Legends**

**Figure S1 Identification of PITA and PISA as selective regulators of p53 towards TIGAR and SCO2, respectively.**

**(A)** Overexpression of KZNF members regulates the level of p53 activity. p53 activity in HCT116 p53^+/+^ cells was measured by pG13L luciferase reporter gene assay. **(B)** Related to Figure 1A. The expression of KZNF members was analyzed by immunoblotting with the indicated antibodies. **(C, D)** PITA and PISA overexpression inhibits the expression of the p53 target genes TIGAR and SCO2, respectively. Lysates from HCT116 p53^+/+^, MCF-7 and U2OS cells transfected with PITA or PISA were analyzed with the antibodies indicated in the figure. **(E)** Distribution of endogenous PITA or PISA in HCT116 p53^+/+^cells was determined by cell fractionation. The fractions were subjected to western blot with the indicated antibodies. **(F)** Subcellular localization of endogenous PITA or PISA in HCT116 p53^+/+^cells. Immunofluorescence analysis with anti-PITA or anti-PISA rabbit polyclonal antibody was performed. Nuclei were stained with DAPI. Images were captured by confocal microscope and merged. Scale bar, 10 μm. **(G)** Images of immunohistochemical staining for PITA or PISA in 20types of tissues. Scale bar, 50μm. **(H)** Columnar plot of PITA or PISA expression in 18 kinds of tissues. **(I)** Expression of PITA or PISA in 27 kinds of tissues form NCBI gene database. **(J, K)** PITA and PISA shRNA or NC (negative control) lentivirus were used to infect HCT116 p53^+/+^ cells. Lentivirus-mediated knockdown of PITA and PISA was detected by immunoblotting and qRT–PCR. **(L)** Overexpression of PITA or PISA inhibits p53 activity. p53 activity in HCT116 p53^+/+^cells was measured by pG13L luciferase reporter gene assay. **(M)** ShRNA-mediated knockdown of endogenous PITA or PISA augments p53 activity in MCF-7 and U2OS cells. p53 activity in MCF-7 and U2OS cells was measured by pG13L luciferase reporter gene assay. **(N, left)** The mRNA levels of glycolysis genes in HCT116 p53^+/+^ cells depleted of PITA were measured by real-time PCR. **(N, right)** The mRNA levels of FAO, OXPHOS, nuclear mitochondrial genes in HCT116 p53^+/+^ cells depleted of PISA were measured by real-time PCR. **(O)** The mRNA levels of TIGAR in PITA-depleted and SCO2 in PISA-depleted MCF-7 and U2OS cells were measured by real-time PCR. **(P)** Coimmunoprecipitation of endogenous PITA or PISA and endogenous p53 from MCF7 and U2OS cells. Western blot analysis of whole-cell lysate (WCL) and immunoprecipitation (IP) with preimmune serum, PITA or PISA-specific antibody, p53 antibody or control mouse IgG are shown. **(Q)** Co-localization of PITA and p53 or PISA and p53 in the nucleus of HCT116 p53^+/+^cells. Immunofluorescence analysis with anti-PITA, anti-PISAand anti-p53 antibody was performed.Nuclei were stained with DAPI. Images were captured by confocal microscope and merged. Scale bar, 10 μm.

Error bars represent mean ± SD for three independent experiments. (**A, H, K-O**; mean and s.e.m., n = 3. **p* < 0.05: two-tailed unpaired *t* test). Unprocessed original scans of blots are shown in Supplementary information, Figure 9.

**Figure S2 PITA and PISA preferentially inhibit the binding of p53 to the target genes TIGAR and SCO2, respectively.**

**(A)** Related to Figure 2A.Expression of PUMA, p21 and GAPDH were detected as indicate antibody with Western blot analysis. **(B)** ChIP assays in HCT116p53^+/+^ cells overexpression of PITA or PISA or in control cells. Twenty-four hours after transfection, ChIP was carried out using a control mouse IgG or an anti-p53 antibody, and RT-PCR was performed for the indicated promoters. **(C)** Related to Supplementary information, Figure 2B. Expression of PUMA, p21 and GAPDH were detected by Western blot analysis. **(D)** ChIP assays in PITA-knocking down and PISA-knocking down or control MCF-7 and U2OS cells. **(E)** The p53-binding sequence in the TIGAR gene is shown. **(F)** *In vitro* EMSA assays. The DNA-binding activity of p53 to oligonucleotides containing the p53 binding sites in the first intron of TIGAR was determined. p53^-/-^ HCT116 cells were transfected with p53 or PITA alone, or cotransfected with p53 and an increasing amount of PITA. After 24 h, the nuclear extracts were purified and used in the EMSA assays. Supershift EMSA using nuclear extracts derived from p53^-/-^ HCT116 cells harbouringMyc-PITA. The PISA antibody and an unlabelled competitor probe were added as indicated. **(G)** EMSA was performed with 100 ng of His-PITA and biotin-labelled PITA/p53-BS1 oligonucleotides. EMSA was performed with 100 ng of His-PITA and biotin-labelled PITA/p53-BS1, PITA/p53-BS2, PITA/p53-BS3, PITA/p53-BS4 oligonucleotides.**(H)** ChIP assays were performed in MCF-7 and U2OS cells using control IgG or anti-PITA or anti-PISA antibody, and PCR was performed. **(I)** MCF-7 and U2OS cells were co-transfected with TIGAR/p53-BS-Luc and PITA or SCO2/p53-BS-Luc and PISA, and the luciferase activity was measured. **(J)** p53^-/-^ and p53^+/+^ HCT116 cells were co-transfected with the mutant TIGAR/p53-BS-mut-Luc and PITA or the mutant SCO2/p53-BS-mut-Luc and PISA, and the luciferase activity was measured. **(K)** Circular maps of genomic PITA data using Circos. **(L)** Gene Ontology (GO) enrichment analysis for biological process ofover-represented in genes. The *p* value of enrichment is shown on Supplementary information, Table 2; the numbers represent the number of associated proteins for each term. **(M)** KEGG analysis of the over-representedgenes in indicated groups. **(N)** Potential of PITA binding motifanalyzed using MEME suite.

Error bars represent mean ± SD for three independent experiments. (**B, D, H-J**; mean and s.e.m., n = 3. **p* < 0.05: two-tailed unpaired *t* test). Unprocessed original scans of blots are shown in Supplementary information, Figure 9.

**Figure S3 PITA and PISA regulate aerobic glycolysis and mitochondrial respiration.**

**(A)** Analysis of HK2 activity in HCT116 p53^+/+^ cells depleted of PITA. **(B)** PFK1 activity, glucose consumption and lactate levels in MCF-7 and U2OS cells depleted of PITA. **(C)** Cytochrome c oxidase (COX) activity, glucose consumption and lactate levels in MCF-7 and U2OS cells depleted of PISA. **(D)** Analysis of concentration of mitochondrial extracted in HCT116 p53^+/+^ cells overexpressing PISA. **(E)** SDH, IDH, KDH, complex Ⅲ activity in HCT116 p53^+/+^ cells overexpressing PISA. **(F)** The mRNA levels of DNA damage repair-related genes (p53R2 and DDB2), drug resistant related gene (MDR), angiogenesis-related gene (Tsp1), self-regulation-related gene (HDM2) in PITA-depleted or PISA-depleted HCT116 p53^+/+^ cells were measured by real-time PCR. **(G)** After knockdown of TIGAR PFK1 activity, Glucose consumption and lactate levels, NADPH levels and GSG/GSSH (ratio) levels in overexpression of PITA HCT116 p53^+/+^ cell lines. **(H)** Cytochrome c oxidase (COX) activity, Glucose consumption, lactate levels, mithchondrial number, and oxygen consumption in knockdown of SCO2 HCT116 p53^+/+^ cell line. **(I)** Upper, The endogenous PITA or PISA protein expression in cancer cell lines was detected by immunoblotting. Lower, Analysis of lactate levels in human cancer cell lines.

Error bars represent mean ± SD for three independent experiments. (**A-H**; mean and s.e.m., n = 3. **p* < 0.05: two-tailed unpaired *t* test). Unprocessed original scans of blots are shown in Supplementary information, Figure 9.

**Figure S4 PITA regulates PFK1 activity and promotes aerobic glycolysis *in vivo*.**

**(A)** Immunoblotting analysis of TIGAR, PITA, p53 and GAPDH in seven representative tissues from adult PITA WT mice and PITA TG mice. Data represent three independent experiments as above. **(B)** PFK1 activity in tissues from WT (n=8) and PITA TG mice (n=6) maintained on a normal diet. **(C)** HK2 activity in muscle from WT (n=8) and PITA TG (n=6) mice maintained on a normal diet. **(D,E)** The mRNA levels of PFK1 or PDK in muscle from WT and PITA TG mice were measured by real-time PCR. **(F)** PDK activity in muscle from WT (n=8) and PITA TG (n=6) mice maintained on a normal diet. (n=3). **(G)** NADPH levels in brain, heart, liver and kidney from WT (n=8) and PITA TG mice (n=6) maintained on a normal diet. **(H)** Glucose consumption and lactate levels in WT and PITA TG MEF cells. **(I)** Analysis of total and single mitochondrial volume and mitochondrial numbers in WT and PITA TG mice. **(J)** ATP level of muscle in in WT and PITA TG mice. **(K)** SDH, IDH and PHOSP activity in WT and PITA TG mice. **(L)** Endogenous PITA was depleted in MEF p53^+/+^ cells by gRNA with three independent target sites. Then they were ligated into three sgRNA expression cassettes of a Cas9 binary vector. CRISPR-mediated knockdown of PITA was detected by immunoblotting and qRT–PCR. **(M)** CRISPR-mediated knockdown of PITA was detected by immunoblotting and qRT–PCR in MEF p53^-/-^ cells.

Error bars represent mean ± SD for three independent experiments. (**D, E, L, M**; mean and s.e.m, n = 3. **p* < 0.05: two-tailed unpaired *t* test). Unprocessed original scans of blots are shown in Supplementary information, Figure S9

Data in (B), (C) and (G-J) represent mean ± SEM **p* < 0.05.

**Figure S5 PISA regulates cytochrome c oxidase activity and dampens mitochondrial respiration *in vivo*.**

(A) Immunoblotting analysis of TIGAR, PISA, p53 and GAPDH in seven representative tissues from adult PISA WT mice and PITA TG mice. Data represent three independent experiments as above. **(B)** COX activity in tissues from WT (n=8) and PISA TG mice (n=6) maintained on a normal diet. **(C)** Glucose consumption and lactate levels in WT and PISA TG MEF cells. **(D)** Fatty acid oxidation was determined by measuring ^3^H_2_O produced by WT and PISA TG MEFs incubated with [9,10(n)-^3^H] palmitate for 2 hours. **(E)** Contribution of glucose to lipid synthesis in WT and PISA TG MEFs. Lipid synthesis was assayed by measuring the amount of ^14^C incorporated into lipids after 2 hours incubation with [6-^14^C] glucose. **(F)** Endogenous PISA was depleted in MEF p53^+/+^ cells by gRNA with three independent target sites. Then they were ligated into three sgRNA expression cassettes of a Cas9 binary vector. CRISPR-mediated knockdown of PITA was detected by immunoblotting and qRT–PCR. **(G)** CRISPR-mediated knockdown of PISA was detected by immunoblotting and qRT–PCRin MEF p53^-/-^ cells.

Error bars represent mean ± SD for three independent experiments. (**F, G**; mean and s.e.m, n = 3. **p* < 0.05: two-tailed unpaired *t* test). Unprocessed original scans of blots are shown in Supplementary information, Figure S9

Data in (B-E) represent mean ± SEM **p* < 0.05.

**Figure S6 PITA and PISA dissociation from p53 upon glucose starvation requires ATM-mediated phosphorylation.**

**(A)** pG13-Luc was cotransfected into HCT116 p53^+/+^ cells with p53 and PITA or PISA as indicated. Twenty-four hours later, cells were cultured in the medium containing glucose with the indicated concentration for 18 h. **(B)** Co-immunoprecipitation of exogenous PITA with p53 or exogenous PISA with p53 in HCT116 p53^-/-^ cells. The cells were treated with GLU- 5mM, H_2_O_2_, etoposide or cisplatin for the indicated times. **(C)** Mass spectrometry of p53 interacting proteins in HCT116 p53^+/+^ cells. The interactions between KRAB-ZFP and p53 were summaried and shown to indicate the dynamic alterations after glucose starvation treatment. (+): LFQ intensity 10^8^, (+++): LFQ intensity 10^10^, (++++): LFQ intensity 10^11^. Arrow means interaction of p53 up, invariant and down. **(D)** ChIP assays were performed to amplify the TIGAR or SCO2 gene. HCT116 p53^+/+^ cells were transfected with Myc-PITA or Myc-PISA and treated withglucose indicated concentration for 18 h. **(E)** Depletion of PITA promotes cell survival under hypoxia. Viability of HCT116 p53^+/+^ cells treated with normoxia or hypoxia was determined via CCK8 assay. **(F)** Stable depletion of PITAHCT116 p53^+/+^cells were exposed to different concentrations of H_2_O_2_ for 24 hours, and the viability of cells was measured byCCK8 assay. **(G)** Sh-NC vs Sh-PITA treated with GLU 5mM 24h, or Sh-NC vs Sh-PISA treated with GLU 5mM 36h in HCT116 p53^+/+^ cells. The total RNA was analyzed using RNA-seq. A total of DEGs showing differential expression were identified in the APN-treatment group (*P*< 0.01). **(H)** Gene Ontology (GO) enrichmentanalysis for biological process, cell component and molecular function of up-regulated proteins. The -log10 *p* value of enrichment is shownon x axis; the numbers represent the number of associated proteins for each term. **(I)** KEGG analysis of the DEGs in indicated groups. **(J)** The p53-signaling associated genes were selected as the target genes in HCT116 p53^-/-^. **(K)** Related to Figure 6F. Expression of Myc-PITA or Myc-PISA, ATM and GAPDH were detected as indicate antibody with Western blot analysis. **(L)** Activity detection of PITA or PISA on p53 in ATM-mutated cells. ATS4 cells were transfected with PITA, PISA together with or without ectopic ATM, and p53 activity was measured by reporter gene assays at 36 hr after transfection. **(M)** HCT116 p53^+/+^ cells were transfected with wild-type ATM siRNA or control siRNA together with PITA or PISA, then treated with glucose starvation for 24-36 hr. p53 activity was measured by reporter gene assays. **(N)** ATS4 cells were transfected with ectopic ATM together with PITA or PISA. Treatment with glucose starvation and measurement of p53 activity were performed as in M. **(O)** HCT116 p53^+/+^ and ATS4 cells were treated with GLU-5mM, and endogenous PITA–p53 and PITA–ATM interactions or PISA–p53 and PISA–ATM interactions were analysed by reciprocal co-immunoprecipitation assays. IP, immunoprecipitation. **(P)** Repression of PITA-S18A and PITA-S18D mutants on p53 activity or PISA-S58A and PITA-S58D mutants on p53 activity were measured in unstressed or stressed cells and compared with wild-type PITA or wild-type PISA by using pG13L luciferase reporter assays. Protein expression was confirmed by western blot analysis. **(Q)** HCT116 p53^+/+^ cells were transfected with ATM siRNA or control siRNA and treated with GLU-5mM. Endogenous PITA–p53 and PITA–ATM interactions or PISA–p53 and PISA–ATM interactions were analyzed by co-IP assays.

Error bars represent mean ± SD for three independent experiments. (**A, D-F, J, L-N,Q**; mean and s.e.m, n = 3. **p* < 0.05: two-tailed unpaired *t* test). Unprocessed original scans of blots are shown in Supplementary information, Figure S9

**Figure S7 PITA and PISA promotes the proliferation of human tumor cells.**

**(A)** PITA and PISA overexpression inhibits the expression of the p53 target genes TIGAR and SCO2, respectively. Lysates from NCM460 cells transfected with PITA or PISA were analyzed with the antibodies indicated in the figure. **(B)** Overexpression of PITA or PISA inhibits p53 activity. p53 activity in NCM460 cells was measured by pG13L luciferase reporter gene assay. Representative results of three independent experiments are shown. **(C)** Co-IP of endogenous PITA or PISA and p53 in different p53 mutation cell lines as indicated. (D) Overexpression of PITA or PISA effect on p53 activityin different p53 mutation cell lines as indicated.p53 activity was measured by pG13L luciferase reporter gene assay.

Error bars represent mean ± SD for three independent experiments. (B, **D**; mean and s.e.m, n = 3. **p* < 0.05: two-tailed unpaired *t* test). Unprocessed original scans of blots are shown in Supplementary information, Figure S9

**Figure S8 PITA and PISA promote tumorigenesis**

**(A)** PITA or PISA shRNA or NC (negative control) shRNAlentivirus were used to infectHCT116 p53^−/−^cells, and cell viability was measured at the indicated time points using CCK8 assays. **(B)** The indicated HCT116 cells were treated with cisplatin or DMSO to induce apoptosis. The percentage of apoptotic cells was measured by Annexin-V staining. **(C)** PITA or PISA overexpressing or control lentivirus were used to infect HCT116 p53^+/+^ cells, and cell viability was measured at the indicated time points using CCK8 assays. **(D)** PITA or PISA overexpressing or control lentivirus were used to infect HCT116 p53^−/−^cells, and cell viability was measured at the indicated time points using CCK8 assays. **(E)** Soft-agar assays with PITA or PISA overexpression in HCT116 p53^−/−^cellswere performed as described. **(F,G)** Analyzing ofmigration and invasion in depletion of PITA or PISAHCT116 p53^−/−^cellswere performed as described.**(H)** Columnar plot of PITA or PISA expression in tumors with different M stages in 80 subjects progression of colorectalcancer . **(I)** Expression of PITA or PITA was detectedinAOM-DSS mice. Modeling colitis-associated cancer with azoxymethane (AOM) and Dextran sulfate sodium (DSS). PITA or PISA was measured at the indicated time points using antibody.

Error bars represent mean ± SD for three independent experiments. (**A-H**; mean and s.e.m, n = 3. **p* < 0.05: two-tailed unpaired *t* test). Unprocessed original scans of blots are shown in Supplementary information, Figure S9

**Figure S9 Unprocessed scans of key western blots.**

**Supplementary References**

1. Rui, Y. *et al.* Axin stimulates p53 functions by activation of HIPK2 kinase through multimeric complex formation. *EMBO J* **23**, 4583-4594 (2004).

2. Bensaad, K. *et al.* TIGAR, a p53-inducible regulator of glycolysis and apoptosis. *Cell* **126**, 107-120 (2006).

3. Finck, B.N. Effects of PPARalpha on cardiac glucose metabolism: a transcriptional equivalent of the glucose-fatty acid cycle? *Expert Rev Cardiovasc Ther* **4**, 161-171 (2006).

4. Hatzivassiliou, G. *et al.* ATP citrate lyase inhibition can suppress tumor cell growth. *Cancer Cell* **8**, 311-321 (2005).
