# Supplementary material for: KRAB-type zinc-finger proteins PITA and PISA specifically regulate p53-dependent glycolysis and mitochondrial respiration
Source: Cell Res. 2018 Feb 21;28(5):572–92. doi: 10.1038/s41422-018-0008-8 (PMC5951852; doi:10.1038/s41422-018-0008-8)

Supplementary Figure 1

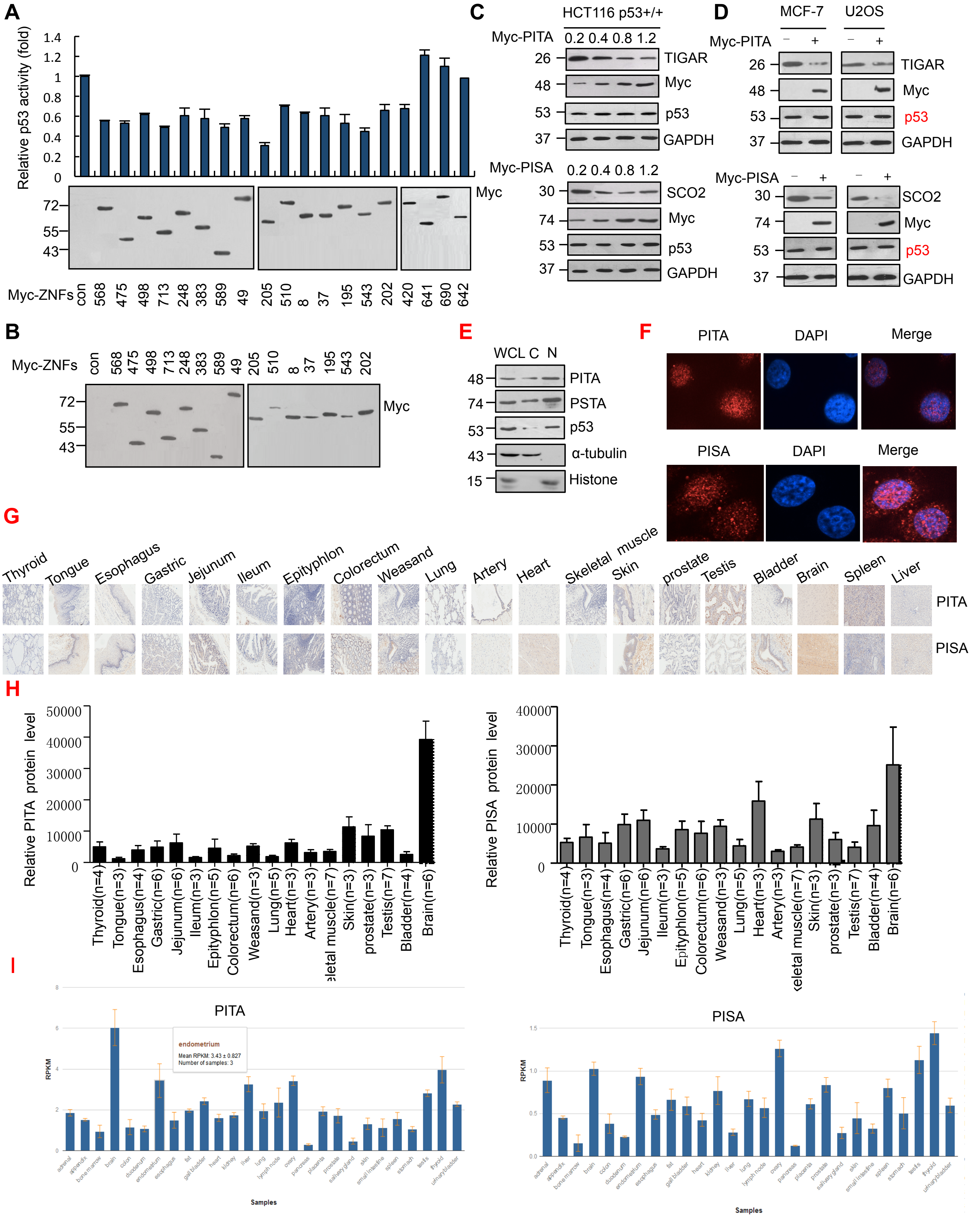

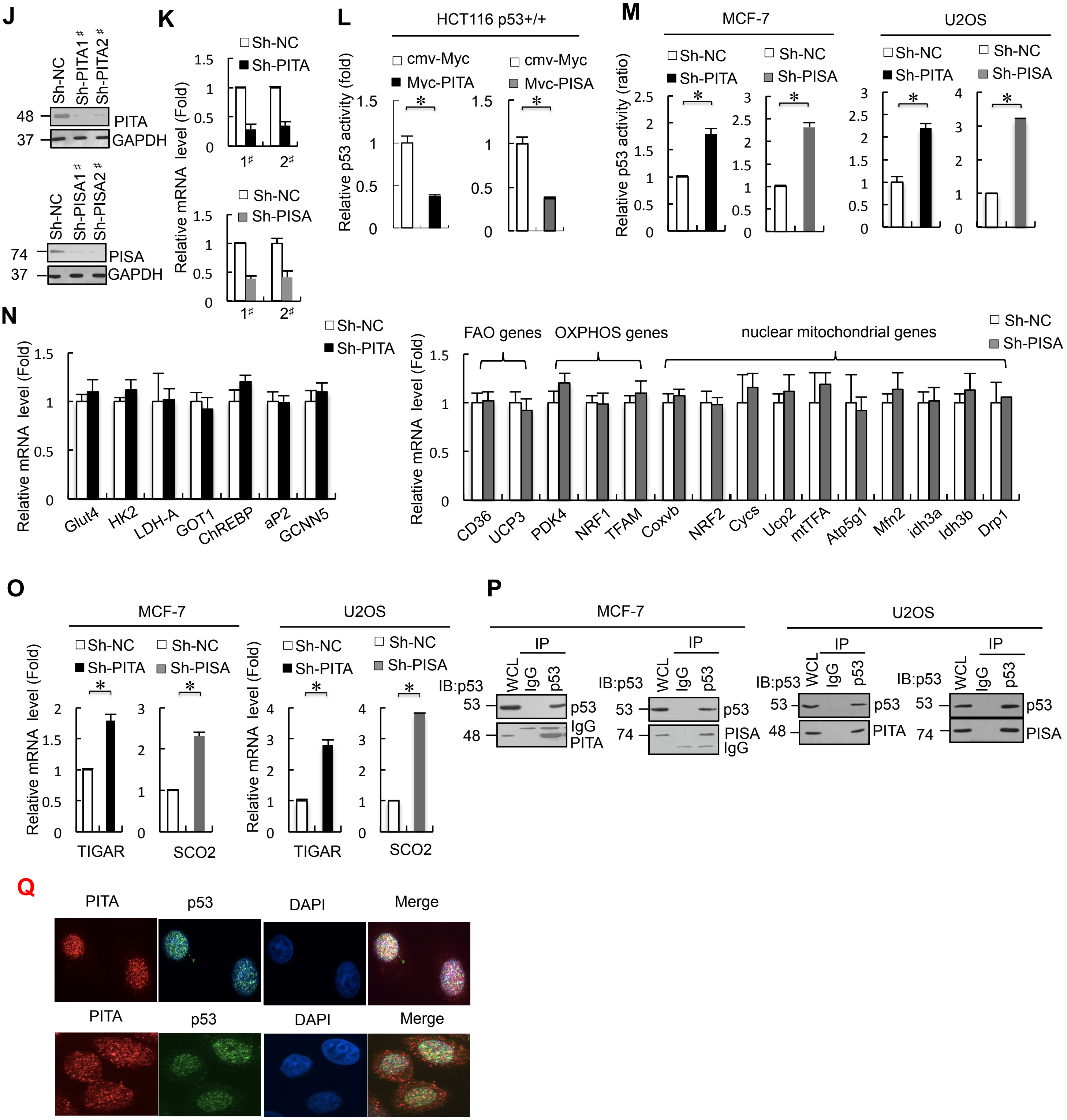

J
Sh-NC
Sh-PITA1 ♯
Sh-PITA2 ♯
48
37
PITA
GAPDH
Sh-NC
Sh-PISA1 ♯
Sh-PISA2 ♯
74
37
PISA
GAPDH
K
Sh-NC
Sh-PITA
Sh-PISA
Relative mRNA level (Fold)
1♯
2♯
L
HCT116 p53+/+
cmv-Myc
Mvc-PITA
Mvc-PISA
Relative p53 activity (fold)
M
MCF-7
U2OS
Sh-NC
Sh-PITA
Sh-PISA
Relative p53 activity (ratio)
N
Sh-NC
Sh-PITA
Sh-PISA
Relative mRNA level (Fold)
Glut4
HK2
LDH-A
GOT1
ChREBP
aP2
GCNN5
FAO genes
OXPHOS genes
nuclear mitochondrial genes
CD36
UCP3
PDK4
NRF1
TFAM
Coxvb
NRF2
Cycs
Ucp2
mtTFA
Atp5g1
Mfn2
idh3a
Idh3b
Drp1
O
MCF-7
U2OS
Sh-NC
Sh-PITA
Sh-PISA
Relative mRNA level (Fold)
TIGAR
SCO2
P
MCF-7
U2OS
IP
IB:p53
WCL
IgG
p53
53
48
74
p53
IgG
PITA
PISA
Q
PITA
p53
DAPI
Merge

Supplementary Figure 2

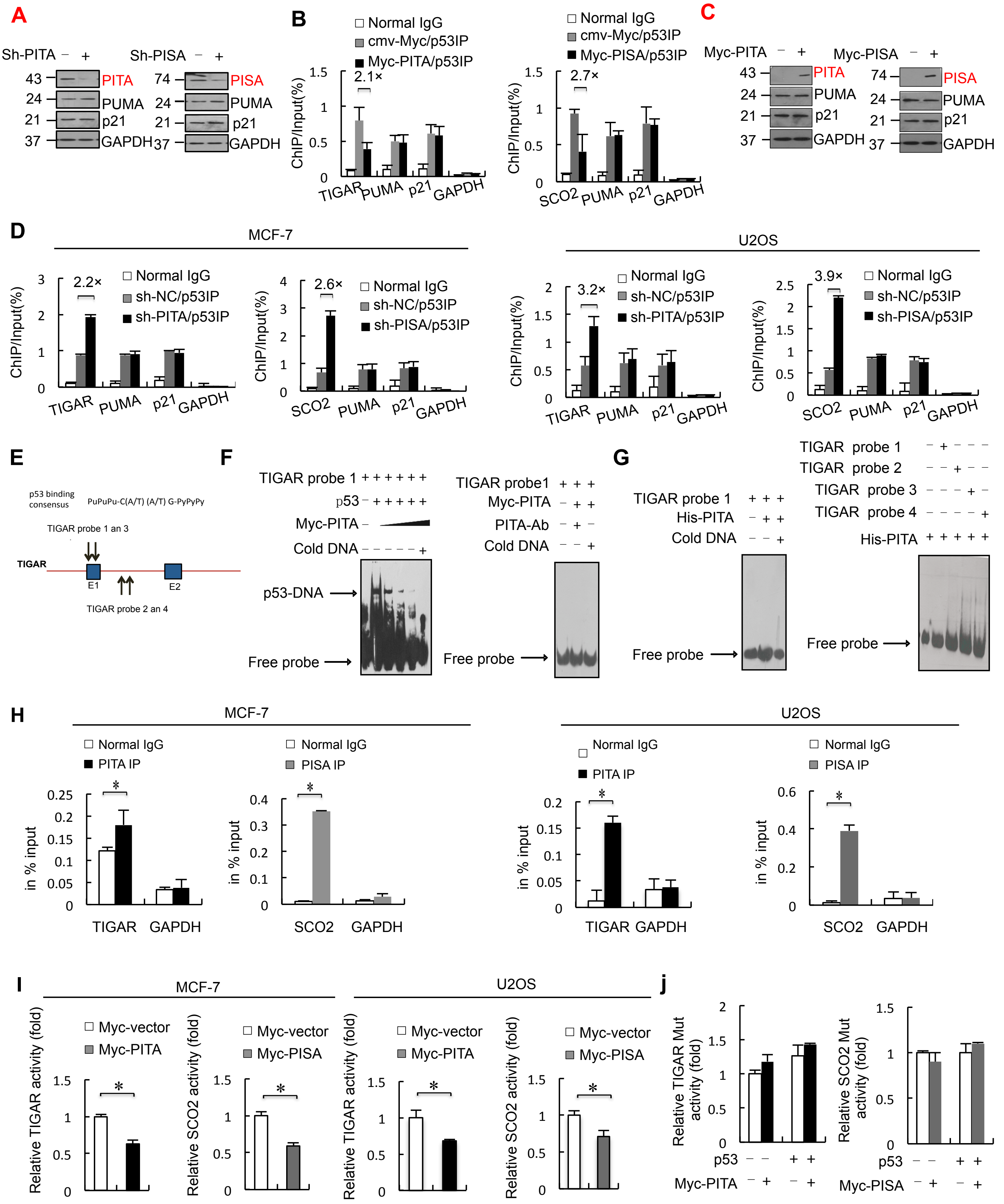

K

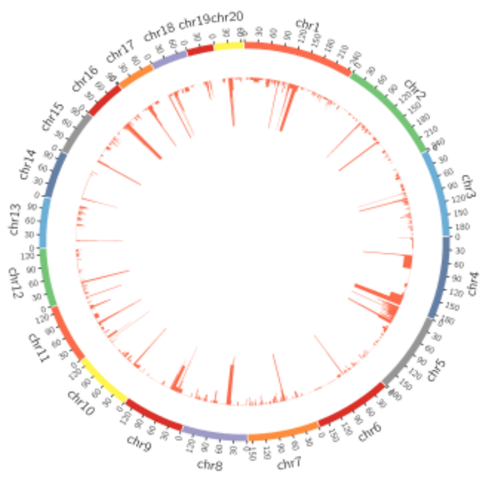
chr1
chr2
chr3
chr4
chr5
chr6
chr7
chr8
chr9
chr10
chr11
chr12
chr13
chr14
chr15
chr16
chr17
chr18
chr19
chr20

L

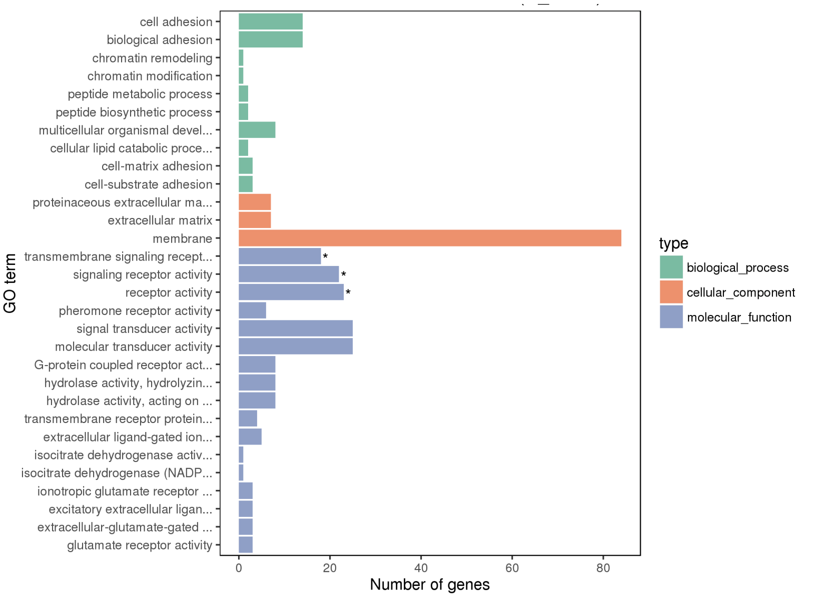
cell adhesion
biological adhesion
chromatin remodeling
chromatin modification
peptide metabolic process
peptide biosynthetic process
multicellular organismal devel...
cellular lipid catabolic proce...
cell-matrix adhesion
cell-substrate adhesion
proteinaceous extracellular ma...
extracellular matrix
membrane
transmembrane signaling recept...
signaling receptor activity
receptor activity
pheromone receptor activity
signal transducer activity
molecular transducer activity
G-protein coupled receptor act...
hydrolase activity, hydrolyzin...
hydrolase activity, acting on ...
transmembrane receptor protein...
extracellular ligand-gated ion...
isocitrate dehydrogenase activ...
isocitrate dehydrogenase (NADP...
ionotropic glutamate receptor ...
excitatory extracellular ligan...
extracellular-glutamate-gated ...
glutamate receptor activity
GO term
0
20
40
60
80
Number of genes
type
biological_process
cellular_component
molecular_function

M

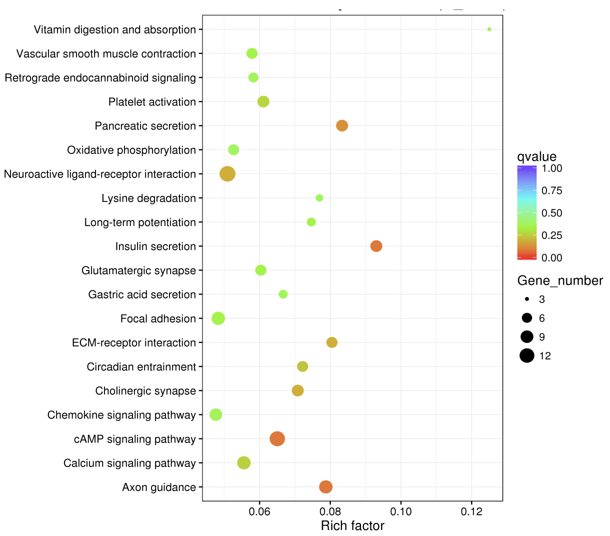
Vitamin digestion and absorption
Vascular smooth muscle contraction
Retrograde endocannabinoid signaling
Platelet activation
Pancreatic secretion
Oxidative phosphorylation
Neuroactive ligand-receptor interaction
Lysine degradation
Long-term potentiation
Insulin secretion
Glutamatergic synapse
Gastric acid secretion
Focal adhesion
ECM-receptor interaction
Circadian entrainment
Cholinergic synapse
Chemokine signaling pathway
cAMP signaling pathway
Calcium signaling pathway
Axon guidance
0.06
0.08
0.10
0.12
Rich factor
qvalue
1.00
0.75
0.50
0.25
0.00
Gene_number
3
6
9
12

N

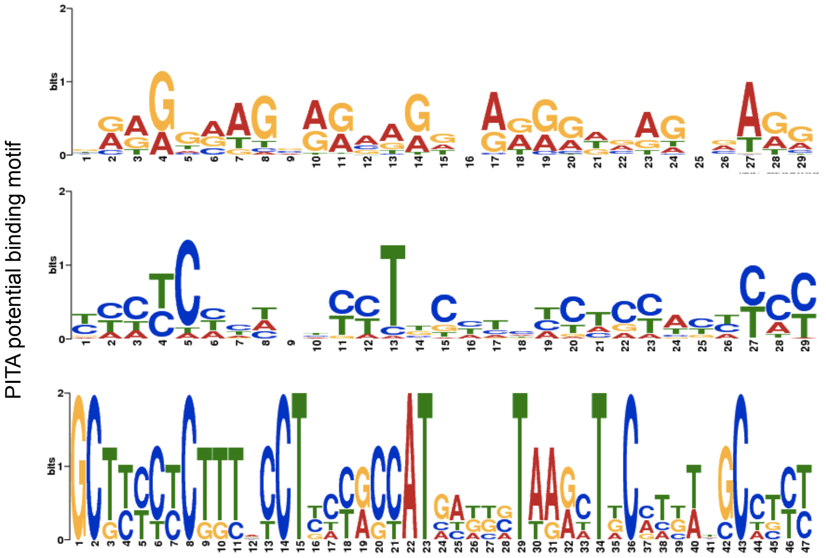
PITA potential binding motif
bits

Supplementary Figure 3

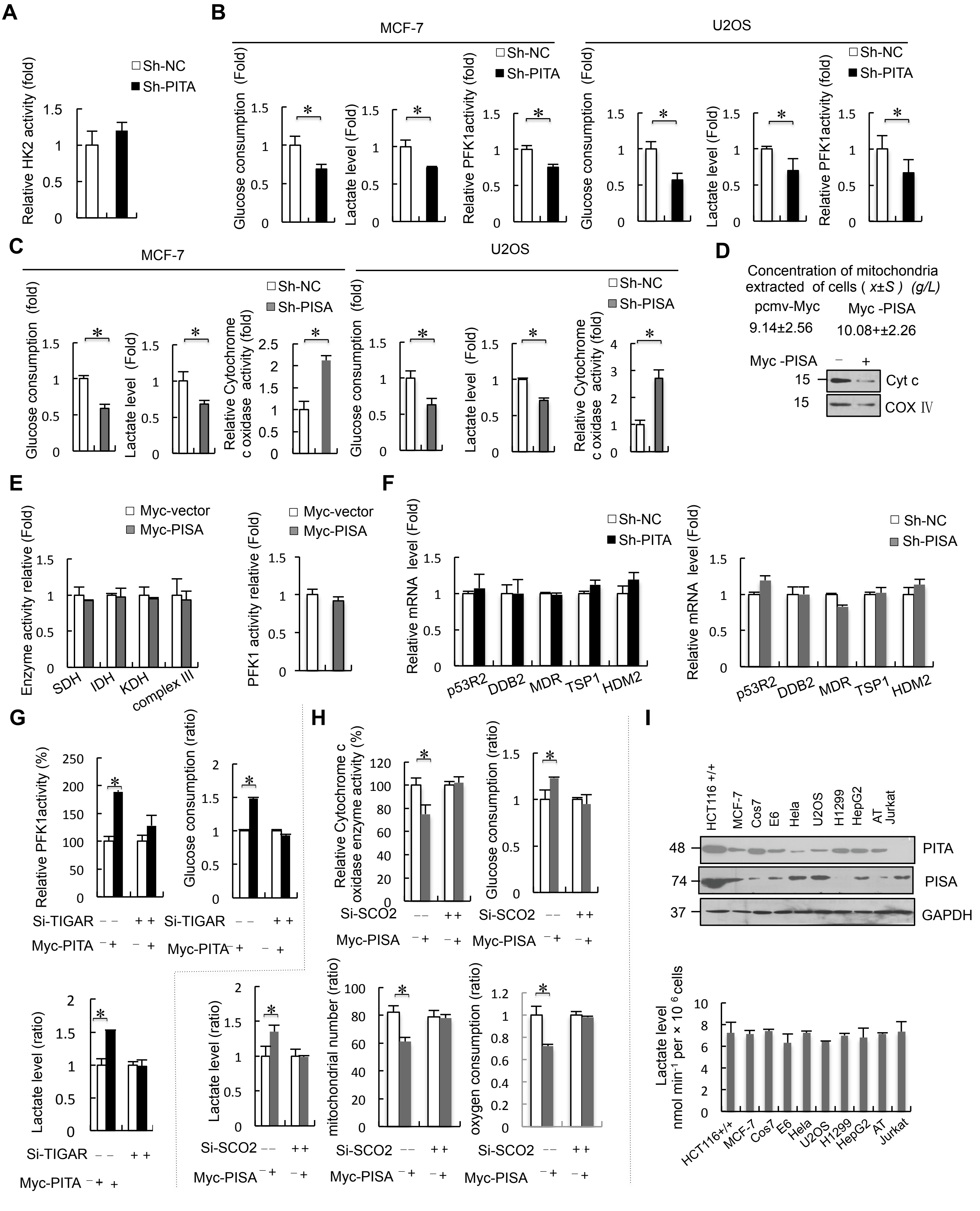

Supplementary Figure 4

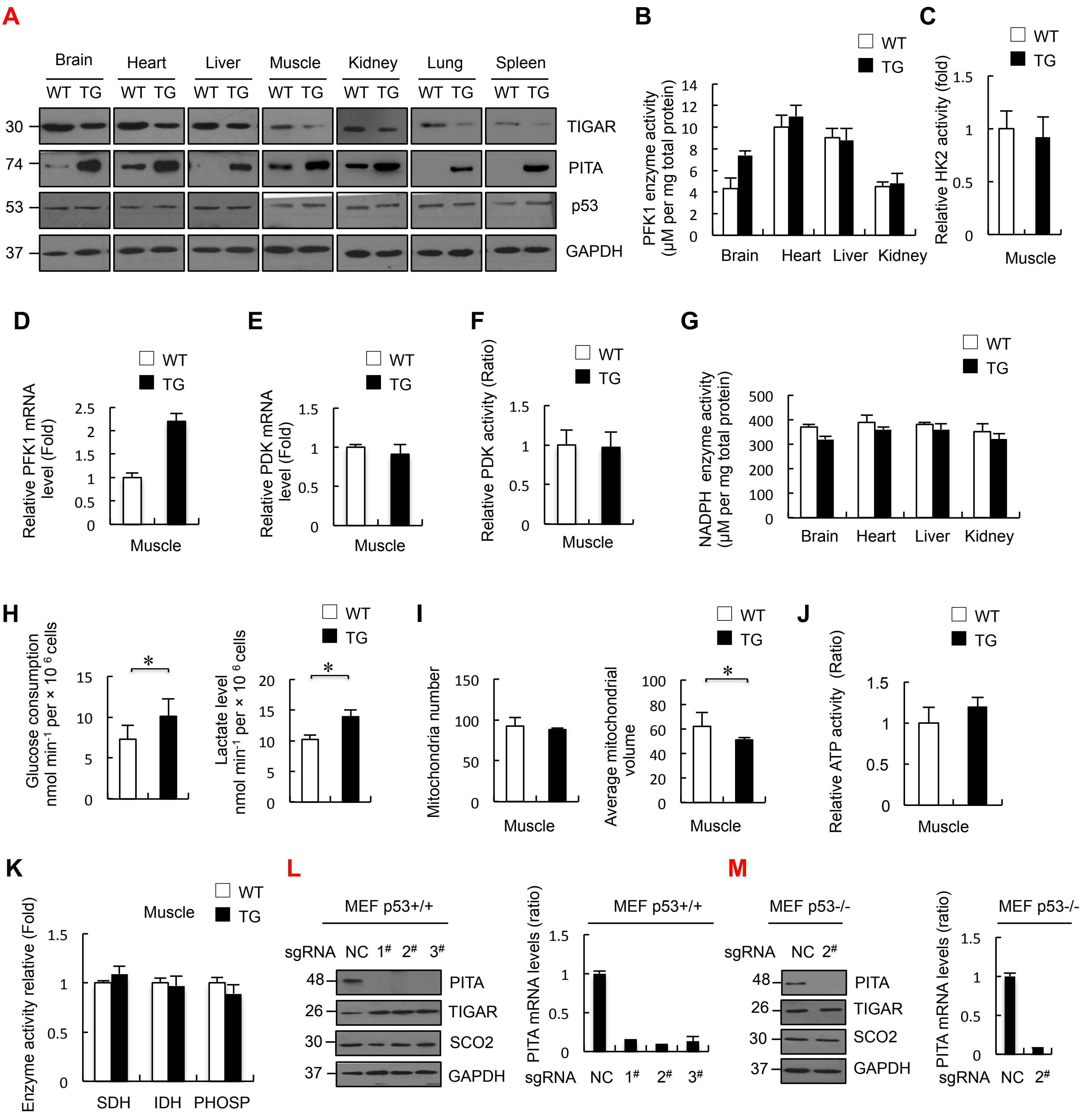

Supplementary Figure 5

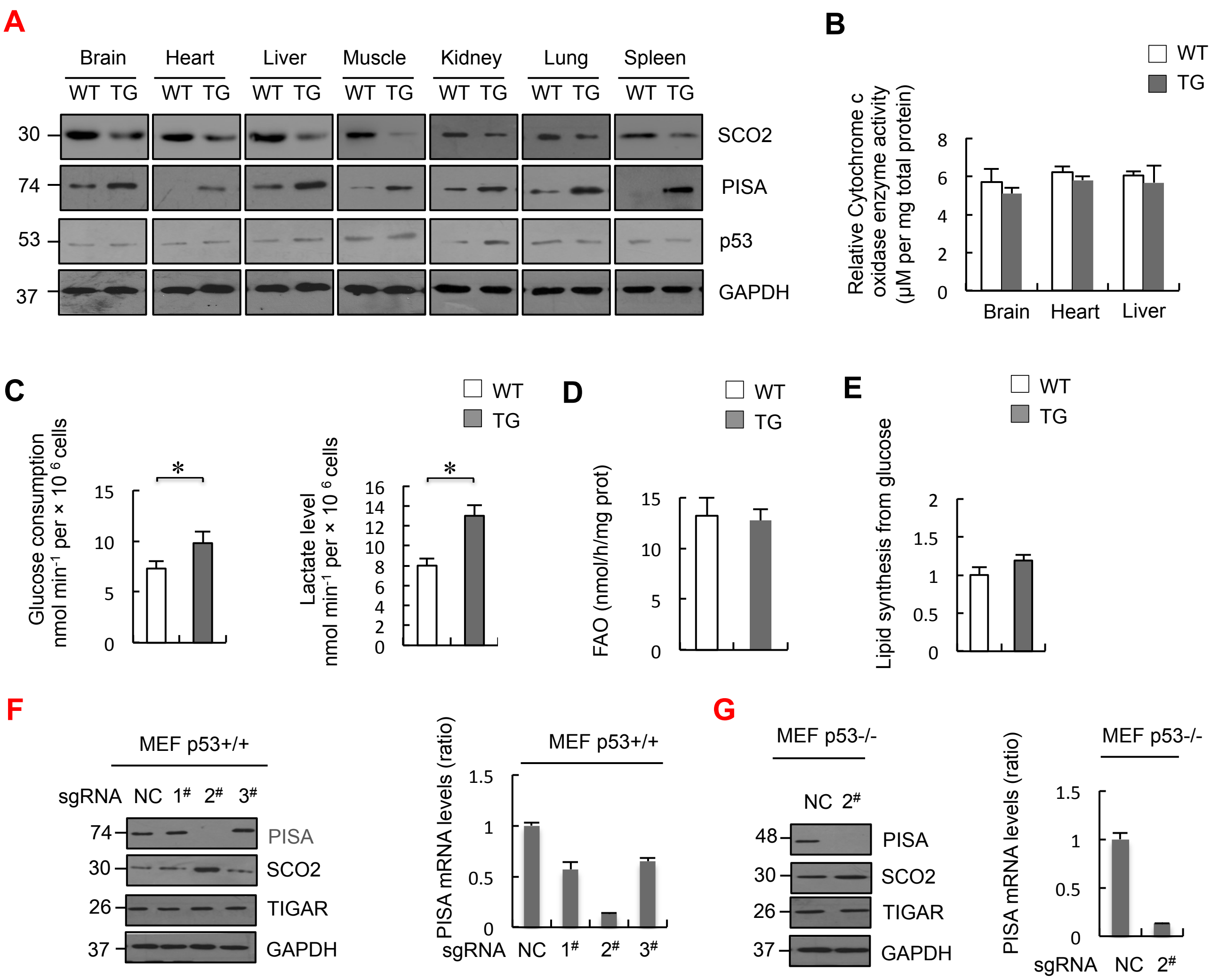

Supplementary Figure 6

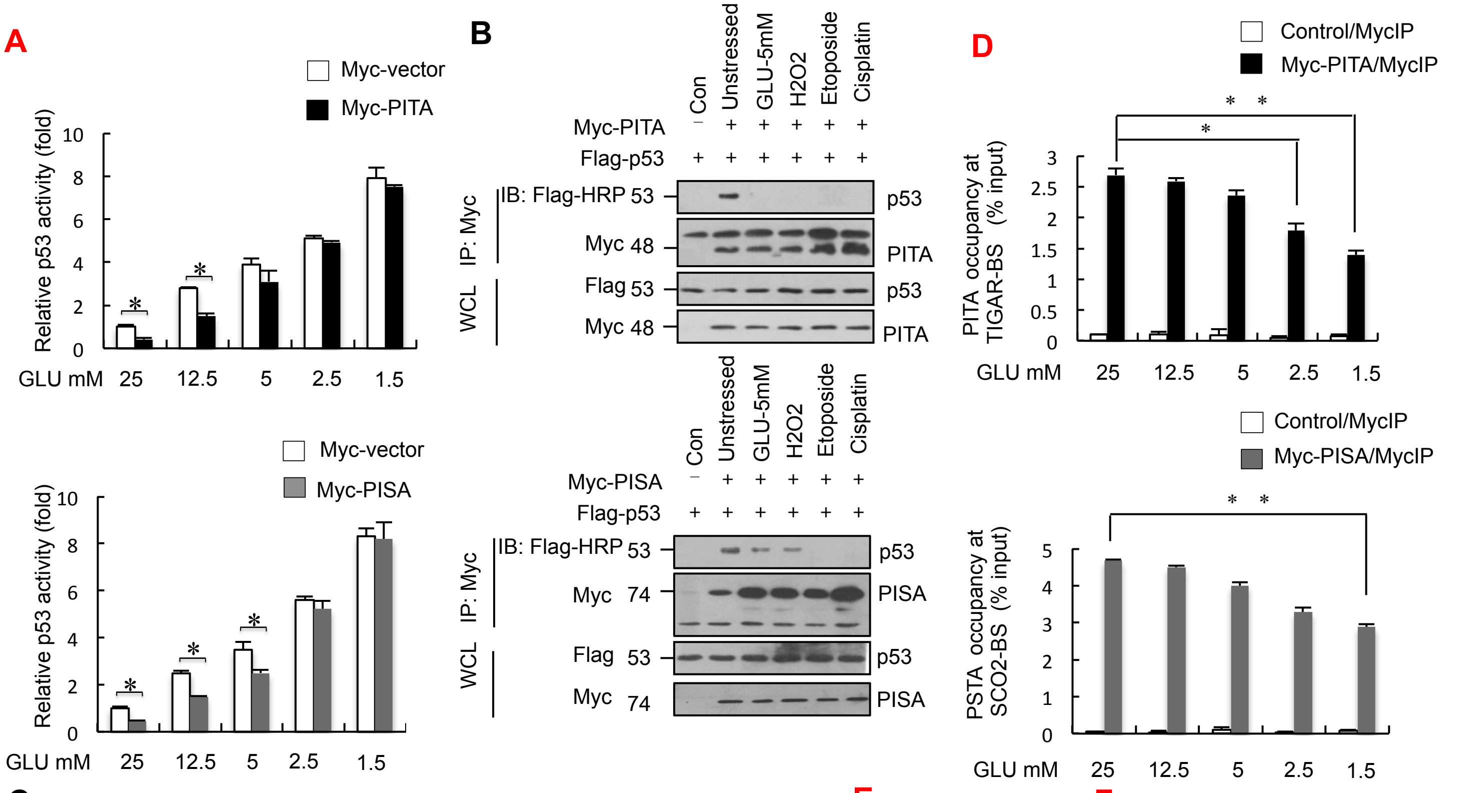

C

Protein ID	Protein name	Con Interaction with p53	24h (GLU-5mM) Interaction with p53	48h(GLU-5mM) Interaction with p53
Q8NDW4	ZNF248	-	+ ⬆	- ⬇
Q6NSZ9	ZNF498	-	- ➡	+ ⬆
O14628	ZNF195	+++	+++ ➡	+++ ➡
P17089	ZNF8	+++	+++ ➡	+++ ➡
Q8NA64	ZNF568(PISA)	+++	+++ ➡	++ ⬇
Q6P2D0	ZNF475(PITA)	++++	+++ ⬇	+++ ⬇

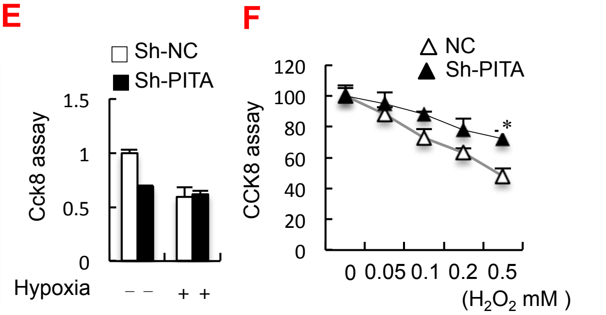

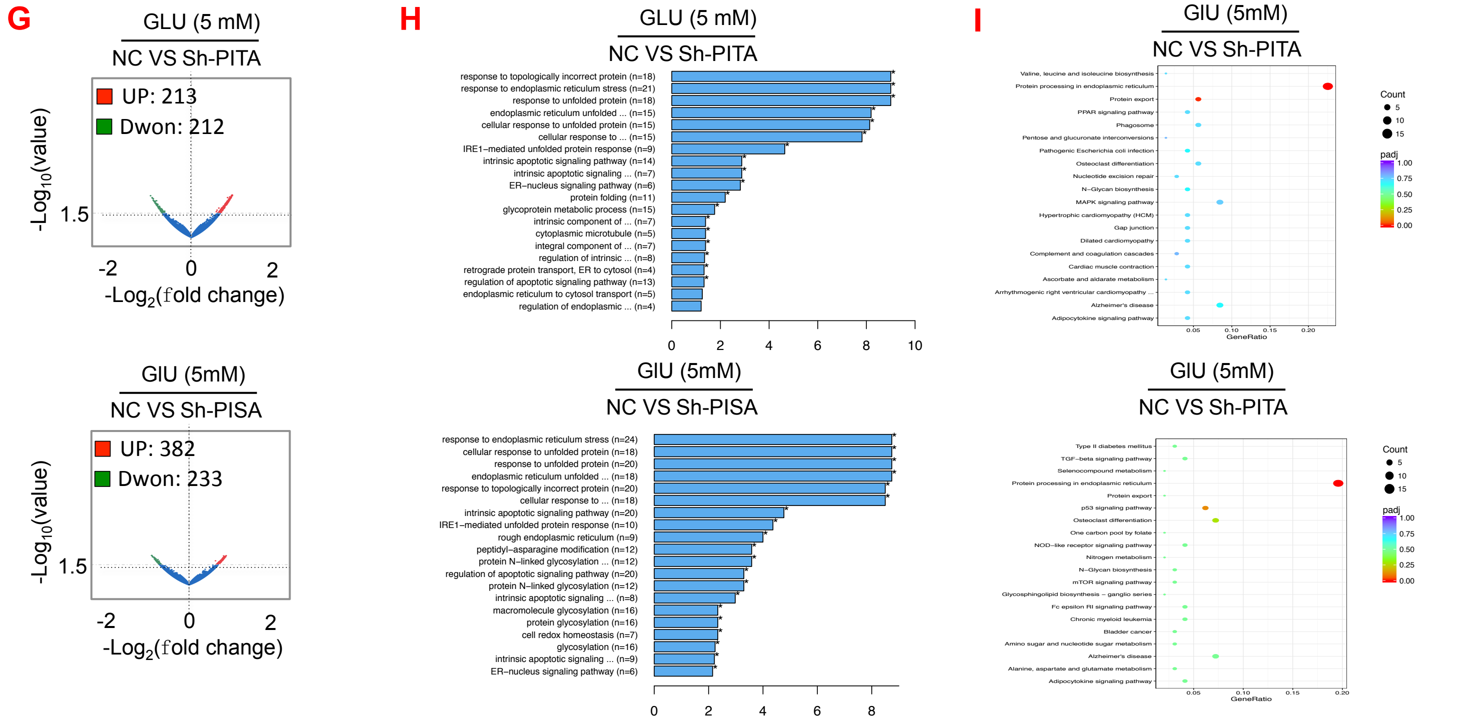

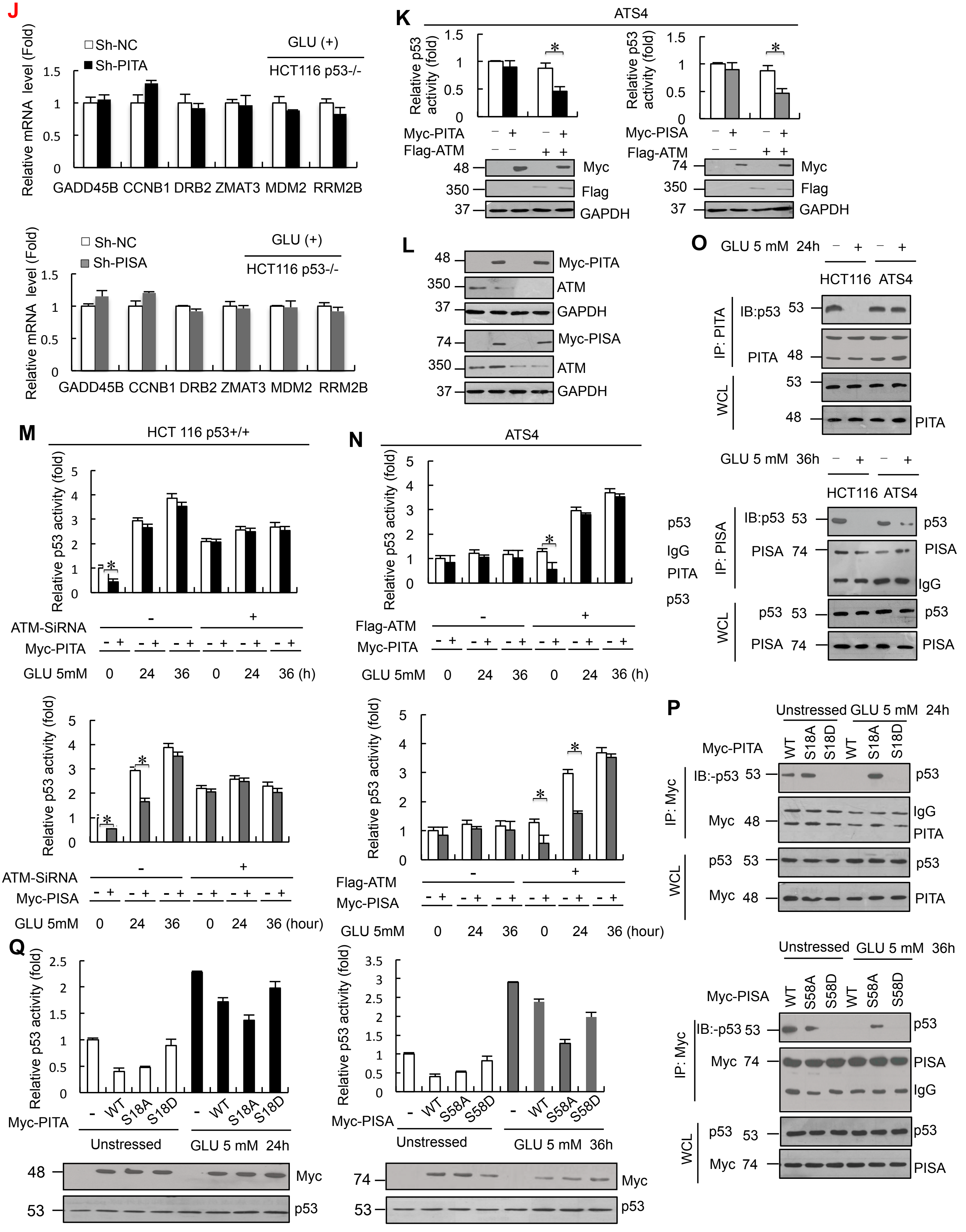

J
K
L
M
N
O
P
Q
ATS4
HCT 116 p53+/+
HCT116 p53-/-
GLU (+)
Sh-NC
Sh-PITA
Sh-PISA
Relative mRNA level (Fold)
GADD45B CCNB1 DRB2 ZMAT3 MDM2 RRM2B
Relative p53 activity (fold)
Myc-PITA
Myc-PISA
Flag-ATM
ATM-SiRNA
GLU 5mM
IP: PITA
IP: PISA
IP: Myc
WCL
IB:p53
Unstressed
GLU 5 mM 24h
GLU 5 mM 36h

Supplementary Figure 7

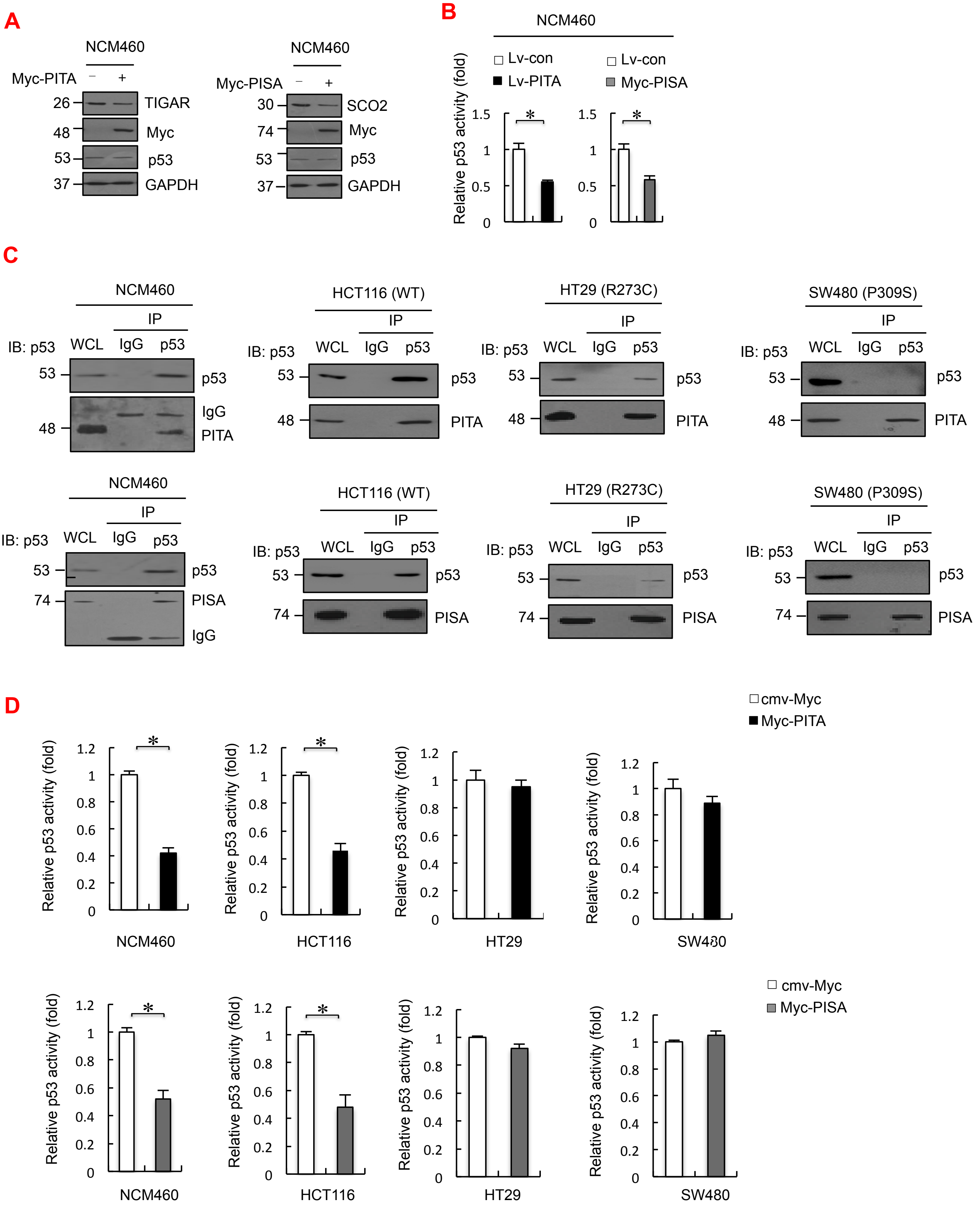

Supplementary Figure 8

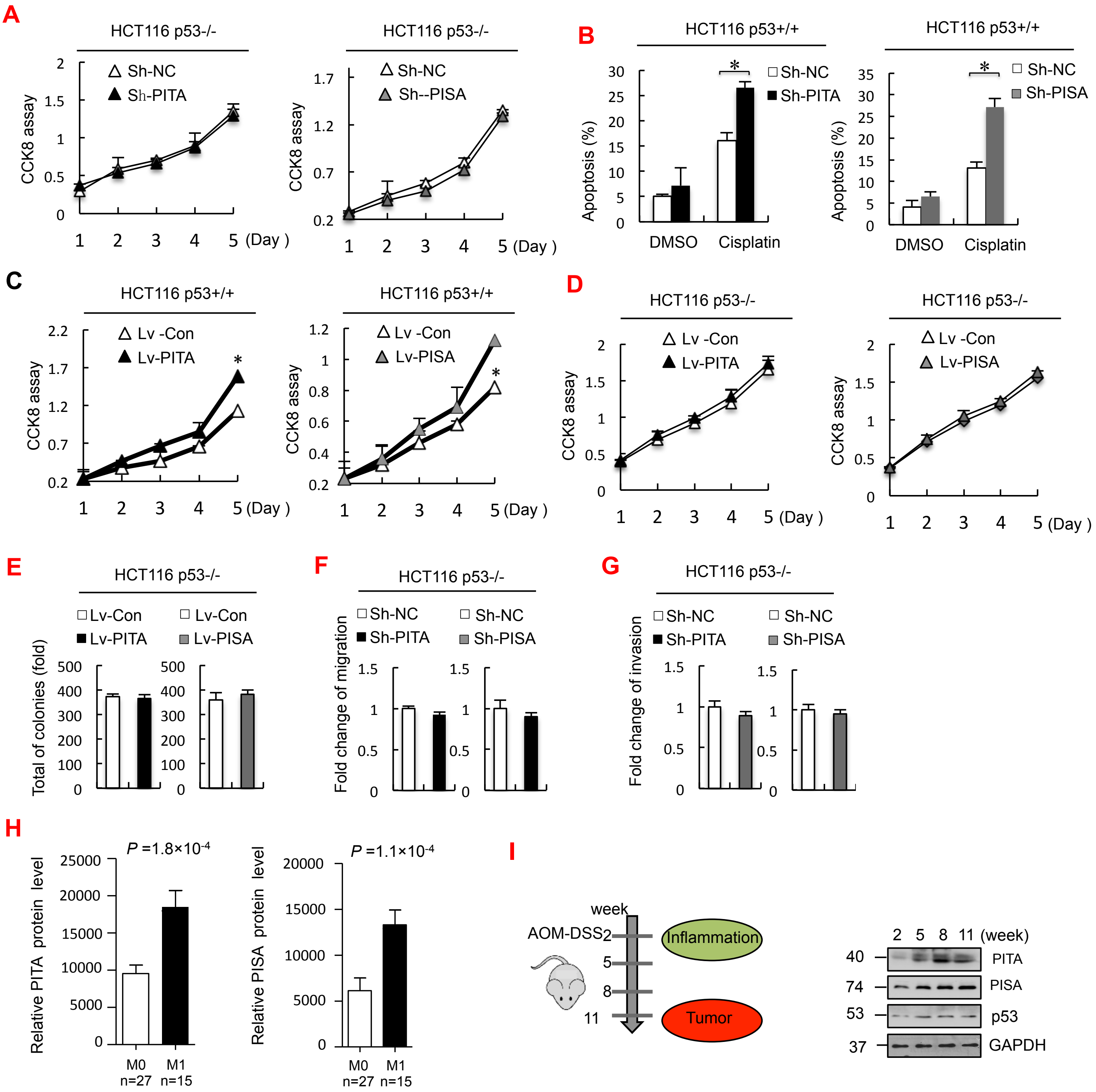

Supplement: Supplementary file 2 — Supplementary information, Figures S1-S8 [file 41422_2018_8_MOESM2_ESM.pdf]
