# Supplementary material for: KRAB-type zinc-finger proteins PITA and PISA specifically regulate p53-dependent glycolysis and mitochondrial respiration
Source: Cell Res. 2018 Feb 21;28(5):572–92. doi: 10.1038/s41422-018-0008-8 (PMC5951852; doi:10.1038/s41422-018-0008-8)

Supplementary Figure 9

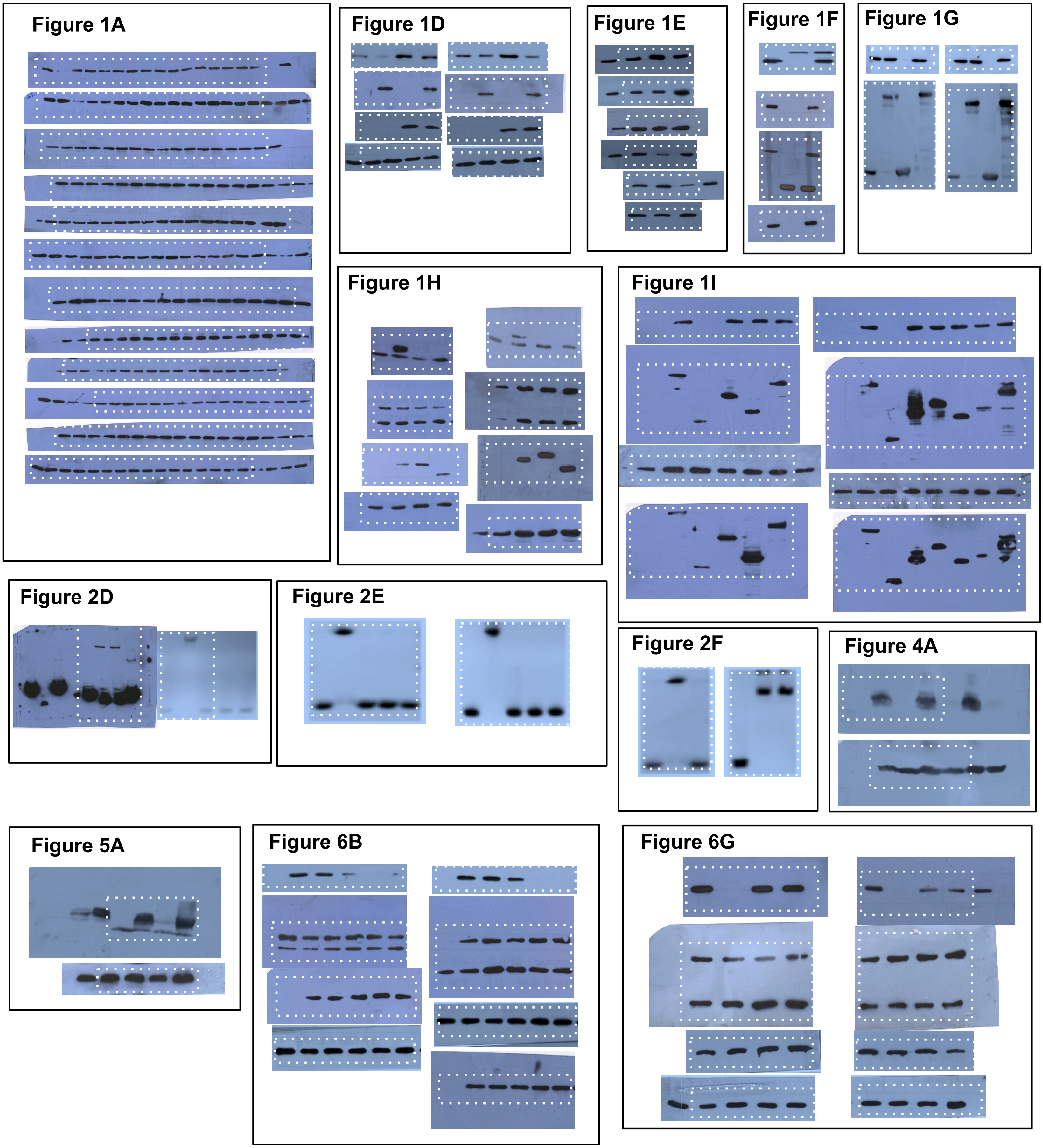

Supp Figure 1A

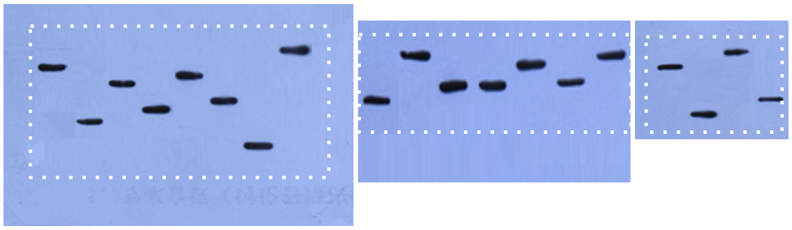

Supp Figure 1B

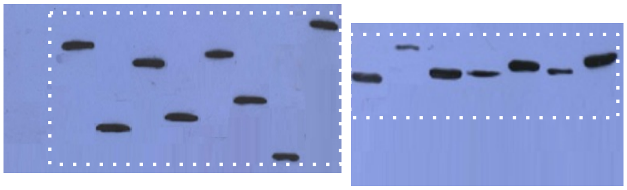

Supp Figure1c

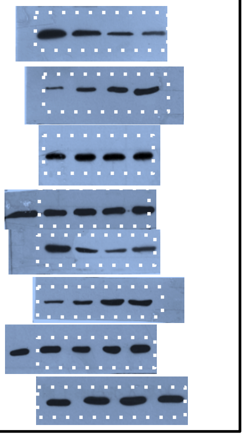

Supp Figure 1D

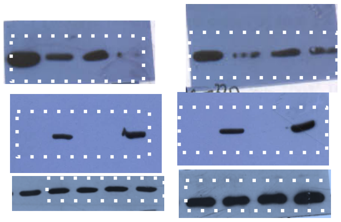

Supp Figure 1F

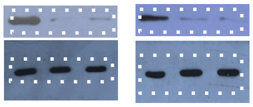

Supp Figure 1E

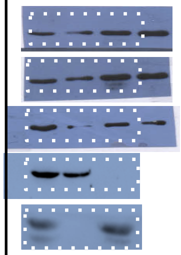

Supp Figure 1P

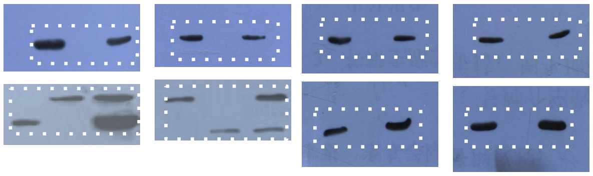

Supp Figure 2A

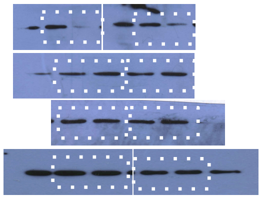

Supp Figure 2C

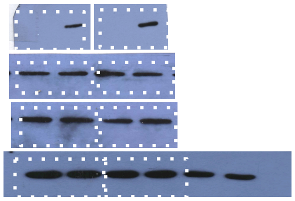

Supp Figure 2F

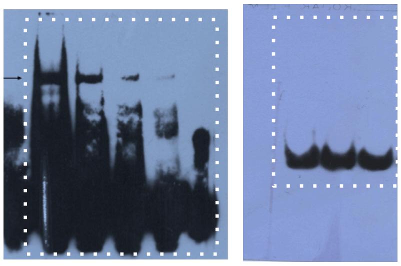

Supp Figure 2G

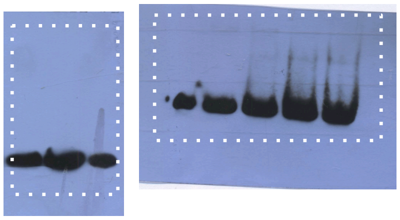

Supp Figure 3D

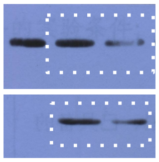

Supp Figure 3I

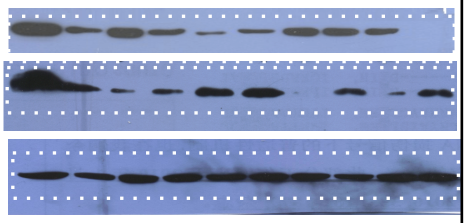

Supp Figure 4A

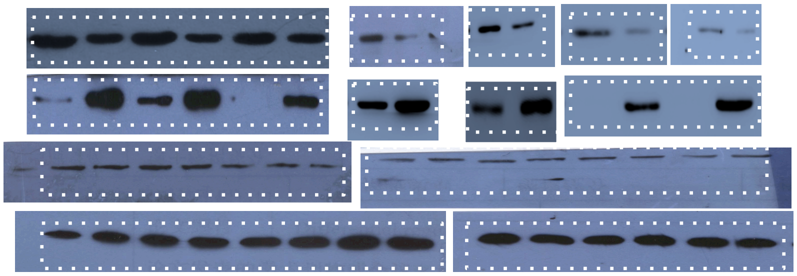

Supp Figure 4L and 4M

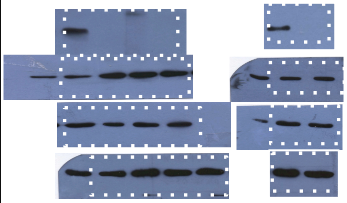

Supp Figure 5A

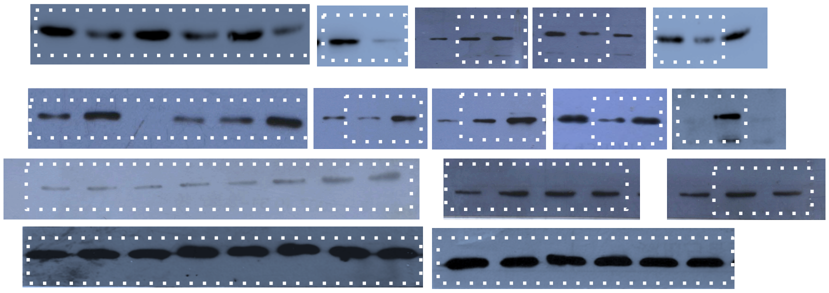

Supp Figure 5F and 5G

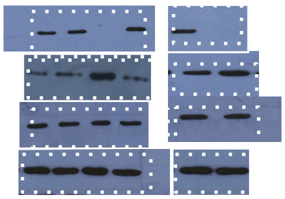

Supp Figure 6A

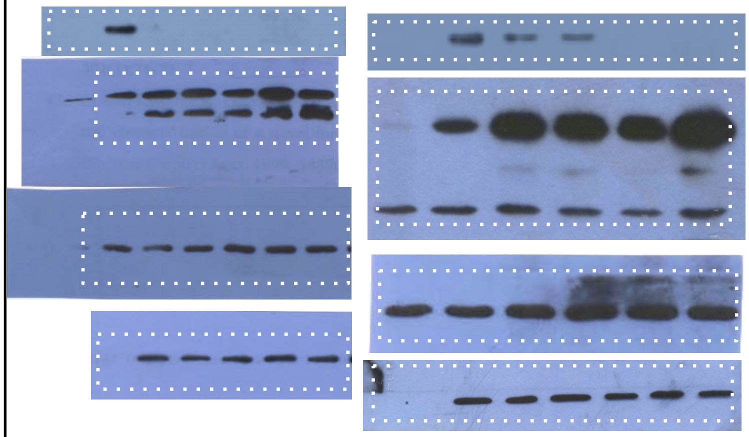

Supp Figure 6K

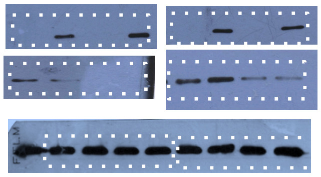

Supp Figure 6L

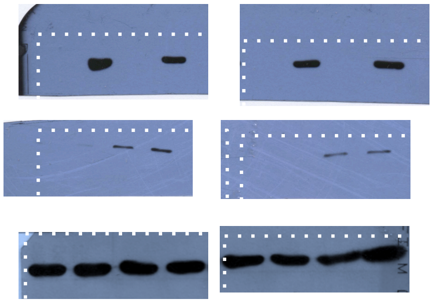

Supp Figure 6O

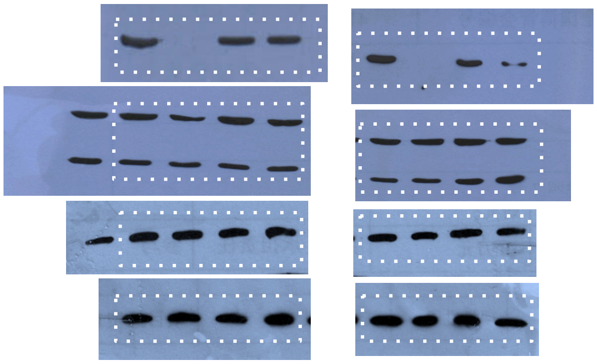

Supp Figure 6P

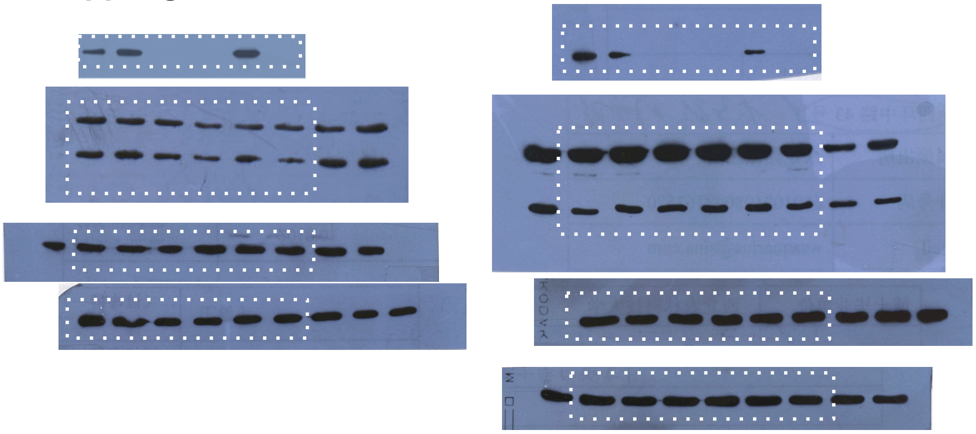

Supp Figure 6Q

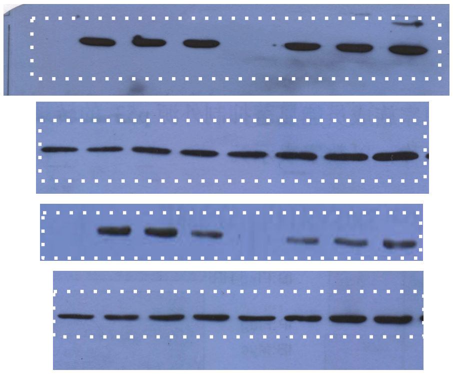

Supp Figure 7A

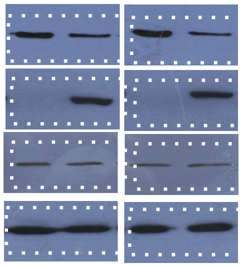

Supp Figure 7C

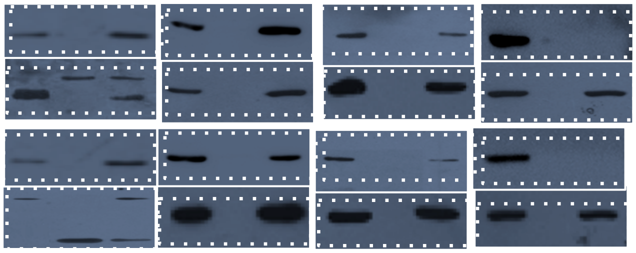

Supplement: Supplementary file 3 — Supplementary information, Figure S9 [file 41422_2018_8_MOESM3_ESM.pdf]
